# Synthesis and Electrolyte Study of Lithium Bis(perfluorinated pinacolato)Borate for Lithium‐Ion Batteries

**DOI:** 10.1002/chem.202502653

**Published:** 2025-11-19

**Authors:** Darren M. C. Ould, Zachary Ruff, Megan E. Penrod, Pravin N. Didwal, Timothy Weiss, Kieran Mylrea, Holly E. Smith, Andrew D. Bond, Robert S. Weatherup, Clare P. Grey, Dominic S. Wright

**Affiliations:** ^1^ Yusuf Hamied Department of Chemistry University of Cambridge Lensfield Road Cambridge CB2 1EW UK; ^2^ Department of Materials University of Oxford Oxford OX1 3PH UK; ^3^ The Faraday Institution Quad One Harwell Science and Innovation Campus Didcot OX11 0RA UK

**Keywords:** batteries, degradation, electrolytes, lithium‐ion batteries, main‐group synthesis

## Abstract

Lithium‐ion batteries are the leading rechargeable battery technology. 1 M lithium hexafluorophosphate (LiPF_6_) dissolved in carbonate solvents is commonly used as the electrolyte; however, LiPF_6_ is hygroscopic and forms toxic HF in the presence of water. This work presents the synthesis of lithium bis(perfluorinated pinacolato)borate salts Li[B(pp)_2_]·*x*DME (*x* = 0 or 1, pp = perfluorinated pinacolato, O_2_C_2_(CF_3_)_4_), which exhibit superior air tolerance and have higher thermal stability than LiPF_6_. Lithium‐ion cycling using 1 M Li[B(pp)_2_]·DME and 0.2 M Li[B(pp)_2_] electrolyte salts was performed, where 0.2 M Li[B(pp)_2_] cycled with higher capacity than 1 M Li[B(pp)_2_]·DME. X‐ray photoelectron spectroscopy on the post‐cycled NMC cathode found a thicker cathode electrolyte interphase with less LiF when using Li[B(pp)_2_]·DME than for LiPF_6_ based electrolyte.

## Graphical abstract

This work showcases the synthesis of lithium bis(perfluorinated pinacolato)borate and investigates its application as an electrolyte salt for lithium‐ion batteries. The synthesized salts give improved air and thermal stability compared to conventionally used lithium hexafluorophosphate. Long‐term battery cycling followed by an electrolyte degradation study has been performed.

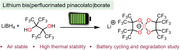

## 1 Introduction

Lithium‐ion batteries (LIBs) are currently the leading rechargeable battery technology, due in part to their high energy density and long cycle life.^[^ ^1^, ^2^, ^3^ ^]^ This technology now sees widespread use in daily life, including in consumer electronics, transportation, and grid energy storage. Moreover, LIBs will help with the transition away from the dependency on fossil fuels. Since the early pioneering work from Goodenough's group on oxide‐based cathodes for LIBs in the 1980s,^[^ ^4^, ^5^, ^6^ ^]^ there has been significant research focus on the development of new cathode materials that give greater capacity and energy density.^[^ ^7^, ^8^ ^]^ To date, there are now many oxide‐based cathode materials that are used in both research and commercial settings. However, while the cathode is critical, the electrolyte plays a crucial and often overlooked role in the battery, as it is largely responsible for the overall lifetime, accessible capacity, and safety.^[^ ^9^, ^10^, ^11^, ^12^ ^]^

The benchmark electrolyte salt for LIBs is LiPF_6_, which is typically dissolved in carbonate solvents.^[^ ^13^ ^]^ LiPF_6_ is widely regarded as being the best compromise of ionic conductivity, thermal stability, chemical stability, and cost compared to other common weakly coordinating salts.^[^ ^14^ ^]^ Moreover, LiPF_6_ dissolved in carbonate solvents was found to form a stable solid‐electrolyte interphase (SEI) on graphite, which is critical for LIB operation. However, problematically, LiPF_6_ is known to have a very low tolerance to moisture, decomposing to give LiF, POF_3_ and HF during both storage and battery cycling.^[^ ^15^ ^]^ The toxicity of HF causes severe safety concerns and can detrimentally affect battery performance.^[^ ^10^ ^]^ Additionally, LiPF_6_ has a low thermal decomposition temperature and is only thermally stable up to 107°C in a dry inert atmosphere, the decomposition temperature then dropping to 87°C in the presence of 300 ppm water.^[^ ^16^ ^]^

**Figure 1:**
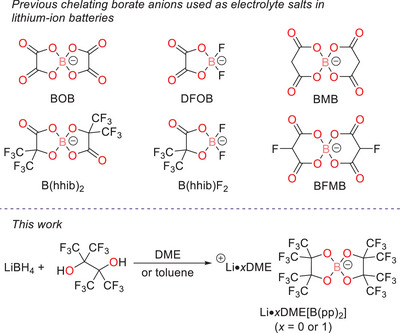
Top: Previously reported chelating borate anions used as electrolyte salts and/or additives for lithium‐ion batteries. Bottom: Overall reaction scheme of the synthesis of electrolyte salts Li[B(pp)_2_]⋅*x*DME (*x* = 0 or 1) studied in this work.

Borate anions with chelating ligands are an attractive class of weakly coordinating anions (Figure 1). The strong B–O bond offers good chemical stability, and both the steric and electronic properties can be tailored through ligand design. Arguably, the most well‐known chelating borate anion for LIB use is bis(oxalate)borate (BOB), and the lithium salt (LiBOB) has been studied extensively both as an electrolyte salt and additive.^[^ ^17^, ^18^, ^19^, ^20^, ^21^, ^22^ ^]^ The low solubility of LiBOB limits its use as an electrolyte salt, but the favorable interphase it forms on the cathode during lithium‐ion battery cycling makes it a popular additive choice. The BOB anion has since been modified to tailor its electrochemical and SEI‐forming properties. For example, the ring size of the cyclic backbone has been expanded to give a six‐membered ring unit in the bis(malonato)borate (BMB) anion.^[^ ^23^, ^24^ ^]^ The addition of a fluorine atom to the BMB anion has also been reported, giving bis(fluoromalonato)borate (LiBFMB). This increases the oxidation potential and facilitates ion dissociation.^[^ ^25^ ^]^ Another alternative to LiBOB is lithium difluoro(oxalato)borate (LiDFOB), where one of the chelating oxalate ligands is replaced with two fluorine atoms.^[^ ^26^, ^27^, ^28^ ^]^

Recently, our group has investigated the bis(perfluorinated pinacolato)borate anion, [B(pp)_2_]^−^ (pp = perfluorinated pinacolato, O_2_C_2_(CF_3_)_4_), for use in sodium‐ion batteries (SIBs).^[^ ^29^ ^]^ Na[B(pp)_2_] has proven to be a promising electrolyte salt for SIBs due to its high tolerance to air and water, stable electrode‐electrolyte interphase, and good capacity retention during cycling. In addition, the magnesium counterpart, Mg[B(pp)_2_]_2_, has been used in magnesium‐ion batteries, where reversible plating and stripping were exhibited.^[^ ^30^ ^]^

The lithium analogue of Na[B(pp)_2_] has previously been reported in a study investigating its fundamental electrochemical properties. It was shown that Li[B(pp)_2_] can be synthesized from perfluoropinacol with lithium hydroxide and boric acid in distilled water. The ionic conductivities in both the molten state and in organic solvents were then evaluated, where an ionic conductivity of 11.1 mS cm^−1^ using a 0.6 M solution in 1,2‐dimethoxyethane solvent (DME) was recorded.^[^ ^31^ ^]^ A further detailed report used pulsed‐field gradient nuclear magnetic resonance (NMR) spectroscopy to measure the diffusivities of the lithium cation and borate anion in Li[B(pp)_2_] and found that the diffusivity of the lithium cation is higher than the borate anion. Moreover, the study found that the salt appeared to fully dissociate in propylene carbonate (PC).^[^ ^32^ ^]^ A separate report examined the physical properties and ionic conductivities of Li[B(pp)_2_] and compared them to LiBOB and LiBMB. Using DMSO as the solvent, the ionic conductivity of 0.5 M Li[B(pp)_2_] was slightly lower than 0.5 M LiBMB (4.86 and 4.99 mS/cm, respectively). Both these values were lower than 0.5 M LiBOB (5.24 mS/cm). The conductivity of these salts was also found to be almost independent of concentration in the range of 0.5–1 M.^[^ ^23^ ^]^

More recently, Li[B(pp)_2_] and seven other lithium borate salts have been investigated for their fundamental physical and electrochemical properties.^[^ ^33^ ^]^ These salts were synthesized from the reaction of LiBF_4_ with an alcohol in carbonate solvents, with trimethylchlorosilane being used as a defluorination reagent. In this study, Li[B(pp)_2_] showed high resistance to hydrolysis, good ionic conductivity, and a wide electrochemical stability window (ESW). However, lithium‐ion cycling was not performed using a Li[B(pp)_2_]‐containing electrolyte, and instead lithium bis(2‐hydroxy‐3,3,3,3’,3’,3’‐hexafluoroisobutirato)borate (Li[B(hhib)_2_], hhib = O_2_C_2_(CF_3_)_2_O) and lithium difluoro(2‐hydroxy‐3,3,3,3’,3’,3’‐hexafluoroisobutirato)borate, Li[B(hhib)F_2_] were investigated (Figure 1). The two borate‐containing electrolytes were cycled at 60°C at a 3C rate and showed superior cycling to using LiBF_4_.^[^ ^33^ ^]^ Lastly, Li[B(pp)_2_] has been used as an electrolyte salt for lithium metal batteries using LiCoO_2_ (LCO) and lithium metal electrodes. This study showed Li[B(pp)_2_] enabled long‐term cycling at both high voltage (4.45 V) and high temperature (60°C) conditions, with 80% capacity retention after 260 cycles.^[^ ^34^ ^]^

In this work, we report a one‐pot synthesis of the lithium bis(perfluorinated pinacolato)borates, Li[B(pp)_2_]⋅*x*DME [*x* = 1 (**1a**) or *x* = 0 (**1b**)], from the addition of inexpensive and readily available lithium borohydride with perfluoropinacol. The salt exists either as a DME‐solvated adduct or nonsolvated salt, depending on the choice of DME or toluene as the reaction solvent. The synthesized salts Li[B(pp)_2_]⋅DME (**1a**) and Li[B(pp)_2_] (**1b**) exhibit superior air stability and higher thermal decomposition temperatures than LiPF_6_. This facilitates more convenient handling, transport, and storage of the salts. Moreover, lithium‐ion cycling using 0.2 M Li[B(pp)_2_] in ethylene carbonate: ethyl methyl carbonate (EC:EMC 3:7 v/v) as the electrolyte showed stable cycling in LiNi_0.8_Mn_0.1_Co_0.1_O_2_ (NMC811) versus graphite cells. A degradation study using 1 M **1a** in EC:EMC found a thicker cathode electrolyte interphase (CEI) with less LiF is formed than with a conventional LiPF_6_‐based electrolyte, as determined by X‐ray photoelectron spectroscopy (XPS).

## 2 Results and Discussion

**Scheme 1:**
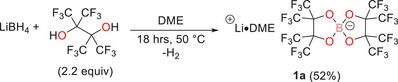
Reaction scheme to synthesize Li[B(pp)_2_]⋅DME (**1a**). DME = 1,2‐dimethoxyethane.

The synthesis of lithium bis(perfluorinated pinacolato)borate, Li[B(pp)_2_]⋅DME (**1a**), followed an analogous procedure to that used for the sodium and magnesium counterparts.^[29],[30]^ Pure lithium borohydride (1.0 equiv) was suspended in 1,2‐dimethoxyethane (DME) solvent and perfluoropinacol (2.2 equiv, caution: alcohol is toxic, see experimental) was added dropwise, with strong effervescence observed. The reaction was left to stir at 50°C for 18 hours, after which it was cooled to room temperature, and the product salt was precipitated by the addition of n‐pentane. Removal of the solvent, and drying under vacuum (1 × 10^−2^ mbar) at 90°C for approximately six hours gave the salt **1a** as a white powder in moderate yield (52%, Scheme 1). Appropriate personal protective equipment should be worn during the synthesis and when handling the synthesized salt.

**Figure 2:**
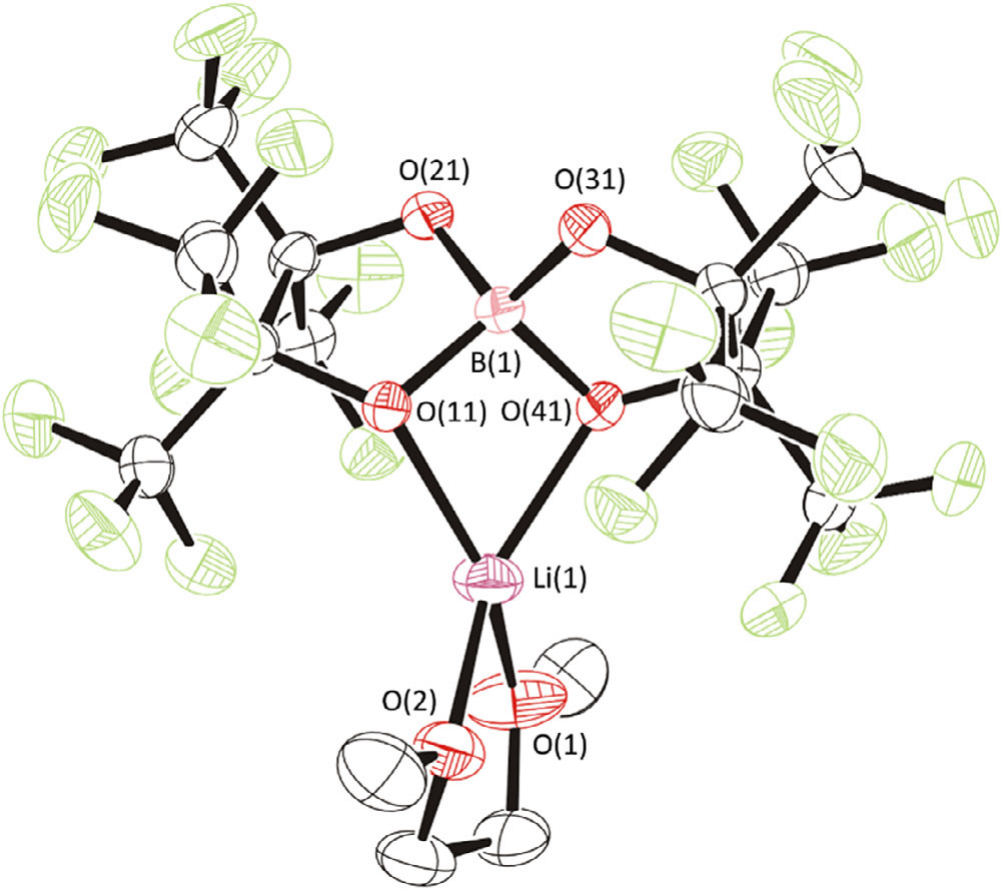
Solid‐state structure of lithium borate Li[B(pp)_2_]⋅DME.^[^ ^35^ ^]^ Pink: Boron, Red: Oxygen, Green: Fluorine, Lilac: lithium. Displacement ellipsoids drawn at 50% probability and H‐atoms omitted. Disorder of the DME ligand in Li[B(pp)_2_]·DME is also omitted for clarity.

The solution‐state ^1^H NMR spectrum confirms that Li[B(pp)_2_]⋅DME (**1a**) is a DME adduct, with signals observed at 3.47 and 3.30 ppm for the two ^1^H environments in DME. These signals integrate to 2:3, respectively, as expected (Figure S5.1.1). The level of DME solvation was quantified by elemental analysis and single‐crystal X‐ray diffraction. The latter revealed that one DME solvent molecule coordinates to the lithium cation within an ion‐paired monomeric structure (Figure 2).

**Scheme 2:**
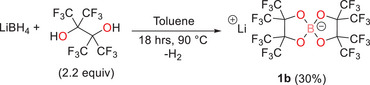
Reaction scheme to synthesize DME‐free Li[B(pp)_2_] (**1b**).

Removal of the DME solvation to produce the unsolvated salt Li[B(pp)_2_] (**1b**) was attempted by heating Li[B(pp)_2_]⋅DME (**1a**) at 120°C under vacuum, but sublimation of the salt meant this approach was not possible. Consequently, solvation‐free **1b** was prepared using the same protocol as **1a**, but using toluene as the solvent. The reaction was heated to 90°C for 18 hours, during which the product salt precipitated out of solution. After cooling to room temperature, the solvent was removed by filtration using a filter cannula, and **1b** was dried under vacuum (1 × 10^−2^ mbar), giving the product as a white powder in moderate yield (30%, Scheme 2). ^1^H NMR spectroscopy and elemental analysis confirmed the absence of solvent coordination (Figure S5.1.7).

The air stability of the salts Li[B(pp)_2_]⋅DME (**1a**) and Li[B(pp)_2_] (**1b**) was then investigated. An electrolyte salt with a high tolerance to air is desirable, as it allows for safe and convenient handling, transport, and storage. Moreover, it has been proposed that under battery operating conditions, ethylene carbonate can be oxidized to produce water and other decomposition products. The water produced can then attack and decompose the salt.^[^ ^36^, ^37^ ^]^

To assess air stability, 0.1 mmol of the salts Li[B(pp)_2_]⋅DME (**1a**) and Li[B(pp)_2_] (**1b**) were exposed to air in vials at room temperature for 5 weeks. After this, solution‐state multinuclear NMR spectroscopy using DMSO‐*d*_6_ solvent was performed to determine if any degradation had taken place. For both **1a** and **1b**, the ^11^B and ^19^F NMR spectra showed no change with air exposure during this time, indicating that no decomposition of the anion had taken place (Figures S5.4.1–S5.4.8). In comparison, 0.1 mmol of LiPF_6_ was left air exposed, which after 24 hours ^31^P NMR spectroscopy showed a complete breakdown of the PF_6_^−^ anion (Figure S5.5.1–S5.5.3).

**Figure 3:**
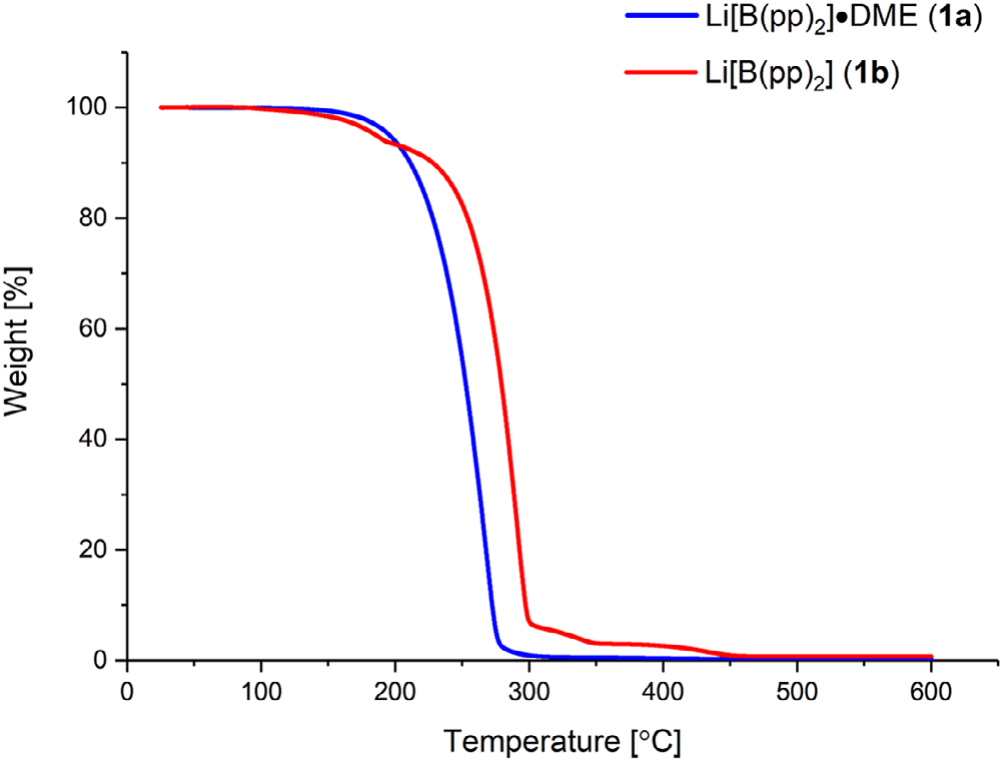
TGA curves for the salts Li[B(pp)_2_]⋅DME (**1a**) and Li[B(pp)_2_] (**1b**). Heating rate of 10 °C min^−1^ and under continuous nitrogen flow.

The thermal properties of the salts Li[B(pp)_2_]⋅DME (**1a**) and Li[B(pp)_2_] (**1b**) were then examined using thermogravimetric analysis (TGA) under dry nitrogen (Figures 3, S1.1, and S1.2). A high decomposition temperature allows elevated drying temperatures to be used to remove coordinated water and potentially enables high‐temperature battery cycling. Both salts **1a** and **1b** were found to have relatively high decomposition temperatures, which are greater than for LiPF_6_ (107°C in a dry atmosphere, 87°C in the presence of 300 ppm water).^[^ ^16^ ^]^ The TGA plot for **1a** shows a one‐step mass loss process, which may result from either thermal decomposition and/or sublimation of the salt (sublimation of **1a** was observed during the synthesis when heated to 120°C under reduced pressure), with an onset temperature of 211°C. For **1b**, a minor mass loss step is observed at an onset temperature of 162°C, before the major decomposition/sublimation occurs at 241°C. The higher onset temperature for the unsolvated salt **1b** compared to the DME adduct **1a** was also seen for the analogous Na[B(pp)_2_]⋅3DME and Na[B(pp)_2_] salts.^[^ ^29^ ^]^

**Figure 4:**
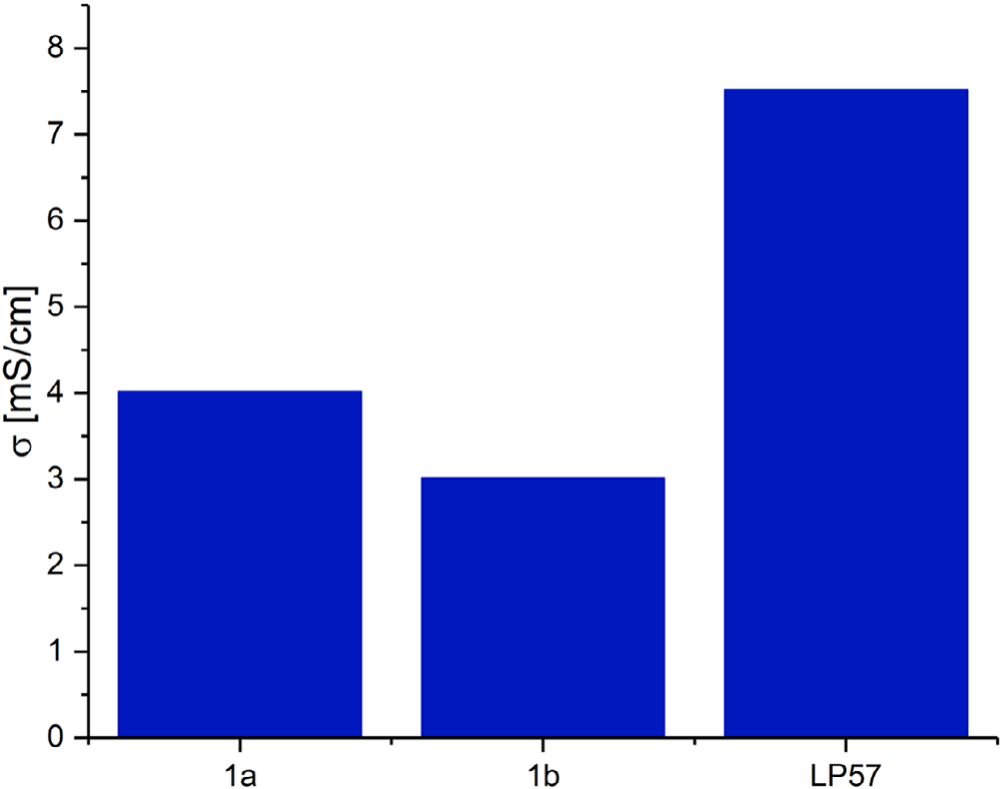
Bulk conductivities of 1 M Li[B(pp)_2_]⋅DME (**1a**), 0.2 M Li[B(pp)_2_] (**1b**), and 1 M LiPF_6_ (LP57) in EC:EMC (3:7 v/v). Measured at 25°C.

Electrochemical studies on Li[B(pp)_2_]⋅DME (**1a**) and Li[B(pp)_2_] (**1b**) in carbonate solvents were then performed. To begin, the bulk conductivities were determined using electrochemical impedance spectroscopy (EIS) by dissolving the salts in a binary EC:EMC (3:7 v/v) mixture. A 1 M solution of **1a** gave a bulk conductivity of 4.0 mS/cm at 25°C. The solubility of DME‐free borate salt **1b** was found to be lower in this solvent system so that only lower concentrations could be explored. A 0.1 M solution of **1b** in EC:EMC gave a bulk conductivity value of 1.6 mS/cm, while the concentration at 0.2 M gave an expected increase in conductivity (to 3.0 mS/cm). For comparison, the bulk conductivity of 1 M LiPF_6_ in EC:EMC (3:7 v/v) (LP57) electrolyte when measured under the same conditions was 7.5 mS/cm (Figure 4).

**Figure 5:**
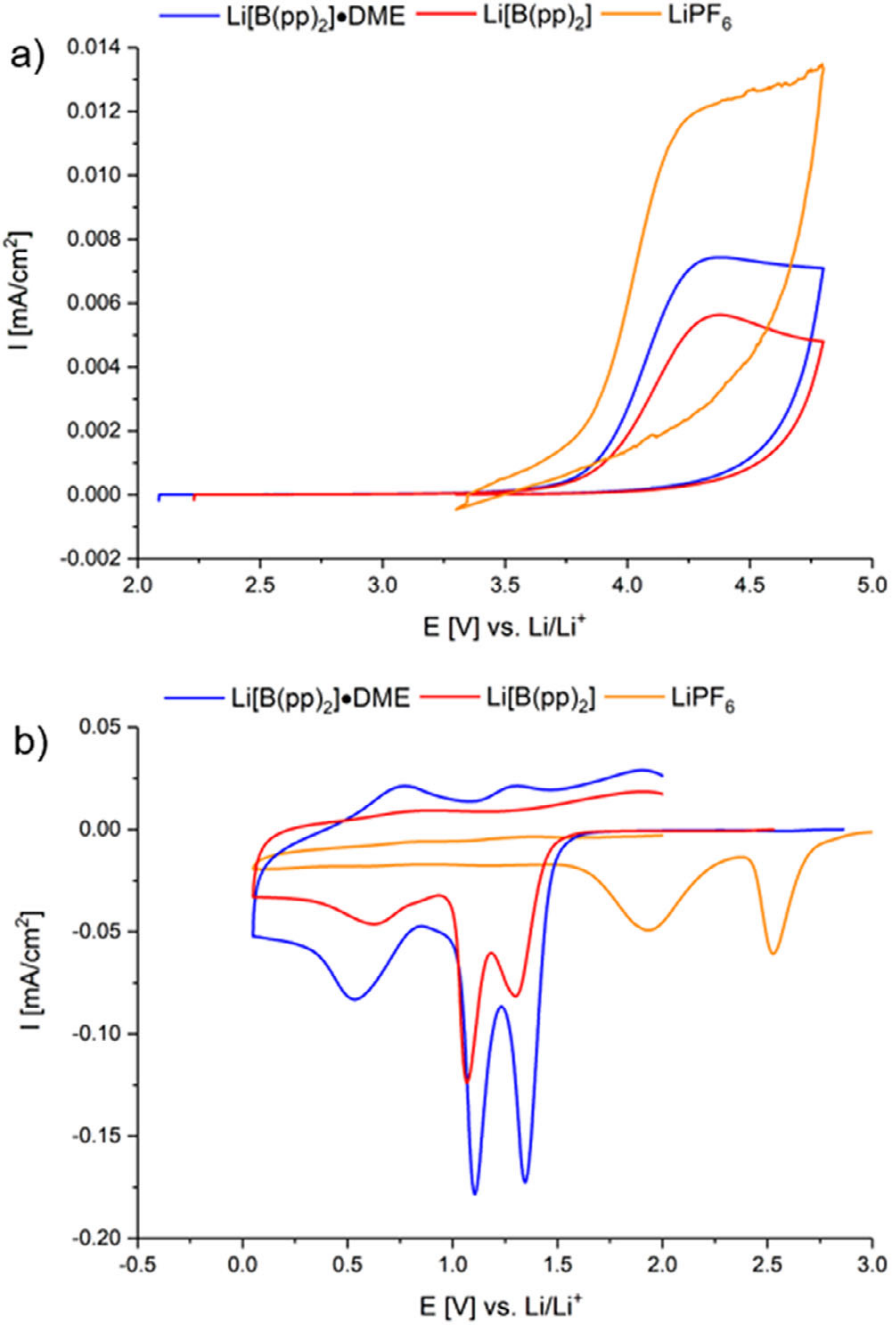
Cyclic voltammetry of 1 M Li[B(pp)_2_]⋅DME (**1a**) (blue), 0.1 M Li[B(pp)_2_] (**1b**) (red), and 1 M LiPF_6_ (LP57, orange) in EC:EMC (3:7 v/v) in a three‐electrode cell. a) Aluminum working electrode; lithium metal counter and pseudo‐reference electrodes. The first cycle was measured at 5 mV/s between open circuit voltage (OCV) and 4.8 V. b) Copper working electrode; lithium metal counter and pseudo‐reference electrodes. The first cycle was measured at 5 mV/s between OCV and 0.05 V.

Following conductivity measurements, the electrochemical stability window (ESW) of both 1 M Li[B(pp)_2_]⋅DME (**1a**) and 0.1 M Li[B(pp)_2_] (**1b**) in EC:EMC (3:7 v/v) toward aluminim and copper was investigated. This was then compared to 1 M LiPF_6_ in EC:EMC (3:7 v/v). The choice of copper and aluminim for the working electrode was due to their use as the current collectors for LIB anodes and cathodes, respectively. When using an aluminim working electrode (Figures 5a and S3.2.1–S3.2.3), the first cycle for 1 M Li[B(pp)_2_]⋅DME (**1a**) in EC:EMC showed oxidation occurred at approximately 3.9 V vs. Li/Li^+^ (oxidation was deemed to occur at the potential at which the current is a quarter of the maximum oxidation current), whereas for 0.1 M Li[B(pp)_2_] (**1b**) in EC:EMC oxidation occurred at 4.0 V vs. Li/Li^+^. The magnitude of the oxidation peak current is lower for 0.1 M **1b** than for 1 M **1a**; however, the difference in conductivity values and concentration between the two electrolytes may account for this. Through integration of current (I) vs time (t) plots, the charge passed for each oxidative CV sweep was calculated (Figure S3.2.7). 1 M **1a** showed a significant reduction in charge passed in the first oxidative sweep and a faster decay in charge passed in subsequent oxidative sweeps, compared with 1 M LiPF_6_. This suggests more effective Al passivation compared with LiPF_6_.

For the CVs performed using copper as the working electrode (Figures 5b and S3.2.4–S3.2.6), the first cycle for both 1 M Li[B(pp)_2_]⋅DME (**1a**) and 0.1 M Li[B(pp)_2_] (**1b**) showed two reductive peaks at 1.1 V and 1.3 V vs. Li/Li^+^. These peaks do not appear in subsequent cycles. In addition, a further broad peak at approximately 0.6 V vs. Li/Li^+^ for **1a** and **1b** was observed, for which the current density decreases with increased cycles. We hypothesize these peaks are likely due to the reduction of copper oxide and electrolyte reduction, resulting in the partial formation of an SEI. The 1 M LiPF_6_ (LP57) electrolyte showed two reductive peaks but at higher voltages, 1.9 V and 2.5 V vs. Li/Li^+^. Reduction of PF_6_^−^‐containing electrolytes has previously been reported in some studies to occur at approximately 2 V vs. Li/Li^+^,^[^ ^38^, ^39^, ^40^ ^]^ with the formation of LiF and PF_3_ hypothesized as reduction products (PF_3_ formed from the reduction of PF_5_).^[^ ^39^ ^]^ More recent work by Menkin et al. has assigned the peaks close to 2.5 V vs. Li/Li^+^ to reduction of the copper fluorides formed in PF_6_^−^ electrolytes and those close to 2.0 V to reduction of copper oxides.^[^ ^41^ ^]^ Note that the exact contribution and potentials associated with those processes were shown to be strongly influenced by how the copper electrode was washed prior to its use. Further comment and deconvolution of reductive CV sweeps is complicated by significant overlap between potential ranges for copper oxide and electrolyte reduction; thus, complete deconvolution is beyond the scope of the current work.

Since the 1 M Li[B(pp)_2_]⋅DME (**1a**) in EC:EMC solvent exhibited good solubility, bulk conductivity, and a reasonable ESW, this electrolyte was tested further under galvanostatic cycling. The electrolyte was tested in coin cells consisting of an NMC811 cathode and a graphite anode, with an upper cut‐off voltage of 4.2 V. The cells were first formed using one C/20 cycle and then cycled with a 1C rate using a constant current, constant voltage charge (CCCV, <C/20 cut‐off) and a 1C constant current discharge for 500 cycles. Every 250 cycles, two C/20 diagnostic cycles were performed as well as an EIS measurement.

**Figure 6:**
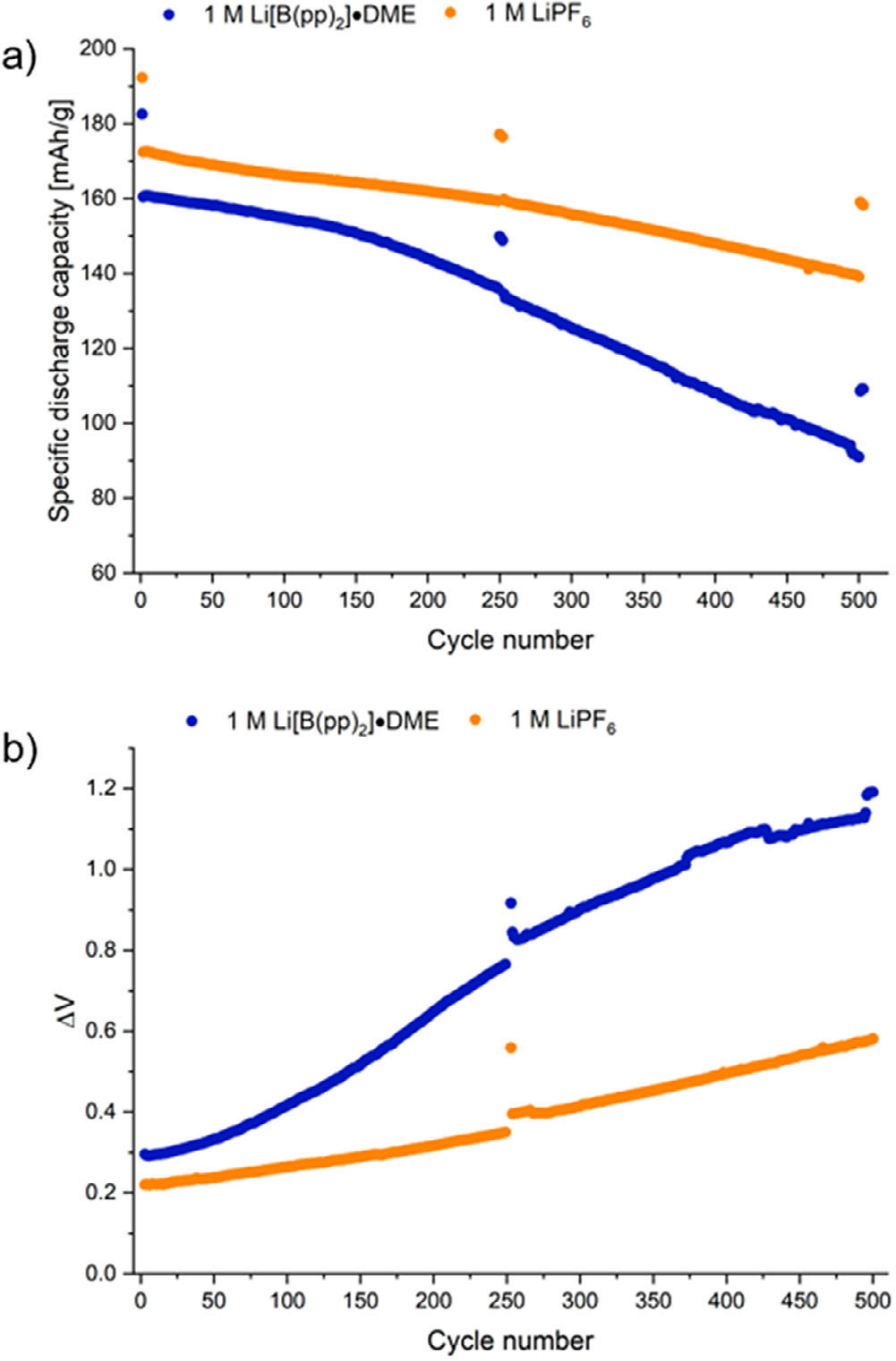
a) Discharge gravimetric capacity vs. cycle number. Active electrode materials are NMC811 and graphite for the cathode and anode, respectively. Approximate constant current rate of C/20 charge and discharge for formation, middle, and end cycles; 1C for remaining cycles using cell voltage limits of 4.2 and 2.5 V. Measured in coin cells. b) Difference between the average voltage on charge and discharge (ΔV) vs. cycle number from the discharge gravimetric capacity vs. cycle number plot in Figure 6a. Electrolytes are 1 M Li[B(pp)_2_]⋅DME (**1a**) in EC:EMC (3:7 v/v) (blue) and 1 M LiPF_6_ in EC:EMC (3:7 v/v) (LP57, orange).

The 1 M Li[B(pp)_2_]⋅DME (**1a**) in EC:EMC electrolyte underwent galvanostatic cycling for the 503‐cycle duration, with an initial capacity of 183 mAh/g (Figure 6a). The capacity retention, as measured by the C/20 diagnostic cycles, for the first 250 cycles was 82%; this then decreased to 60% after 500 cycles. In comparison, the 1 M LiPF_6_ in EC:EMC (LP57) electrolyte cycled with a slightly higher initial capacity of 192 mAh/g and after 503 cycles had a capacity retention of 82%. The Coulombic efficiencies are shown in Figure S3.4.1, where 1 M LiPF_6_ gave slightly higher Coulombic efficiencies throughout the 503 cycles. The voltage vs. capacity plots for the 2^nd^, 255^th^, and 500^th^ cycles have been compared for the two electrolytes, which shows the slightly greater capacity loss for the **1a** electrolyte (Figures S3.4.2–S3.4.5). The difference between the average voltage on charge and discharge was plotted as a function of cycle number to quantify the voltage polarization during cycling (Figure 6b). Although both cells underwent polarization during cycling, the increase for **1a** was larger.

**Figure 7:**
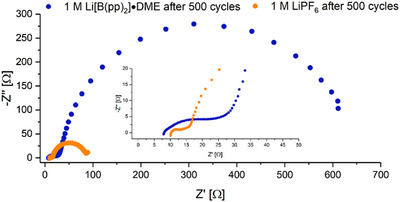
Nyquist plots of 1 M Li[B(pp)_2_]⋅DME (**1a**) in EC:EMC (3:7 v/v) (blue) and 1 M LiPF_6_ EC:EMC (LP57, orange) electrolytes after 500 cycles. Measured using impedance spectroscopy with a frequency range of 1 MHz–0.01 Hz.

To investigate the causes for the differences in capacity retention and polarization after galvanostatic cycling of Li[B(pp)_2_]⋅DME (**1a**) and LiPF_6_ electrolytes during cycling, EIS and X‐ray photoelectron spectroscopy (XPS) measurements of the cathode were performed. The Nyquist plots of EIS measurements for the **1a**‐ and LiPF_6_‐containing cells after 500 cycles are shown in Figure 7. Overlays of the Nyquist plots after formation and 250 cycles are shown in the supporting information, Figures S3.3.1–S3.3.5. The larger size of the lower frequency semi‐circle for **1a** after 500 cycles is likely to be due to the larger increase in the charge transfer resistance. While the increased charge transfer resistance in principle could be associated with either the cathode or anode, in the NMC811/graphite system, the increase has been reported to be associated more with the CEI.^[^ ^42^, ^43^ ^]^

**Figure 8:**
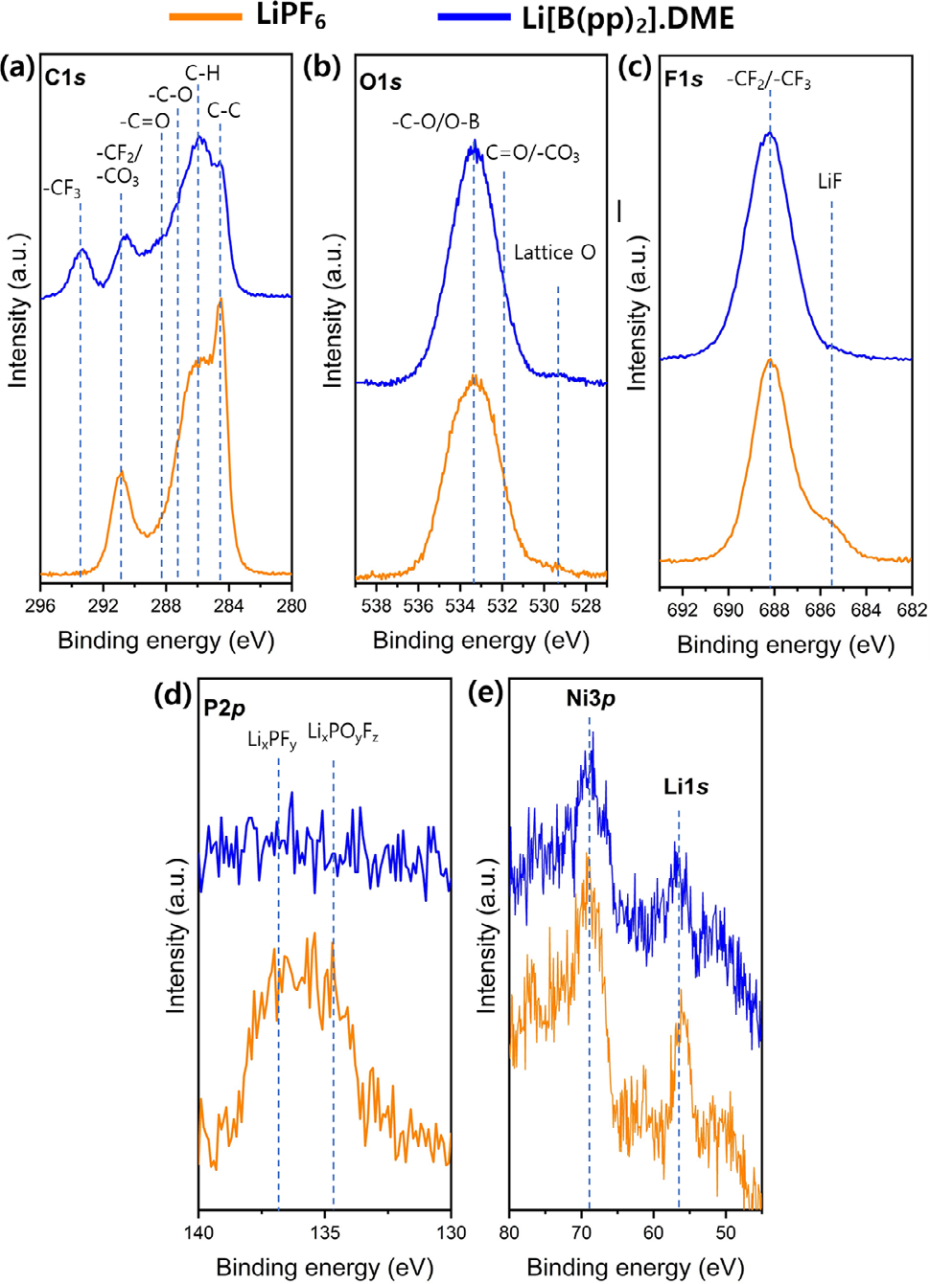
a) C1s, b) O1s, c) F1s, d) P2p, and e) Ni3p/Li1s XPS spectra of the NMC811 cathode using 1 M Li[B(pp)_2_]⋅DME (**1a**) (blue) in EC:EMC (3:7 v/v) and LP57 (orange) electrolytes after 500 cycles.

The CEI is a critical component in determining the cycling stability of LIBs and consists of organic and inorganic species from electrolyte and electrode degradation. Depending on the thickness and composition, the CEI may hinder Li^+^ migration, resulting in poorer charge transfer kinetics.^[^ ^44^ ^]^ Thus, in order to determine the nature and thickness of the CEI when using 1 M Li[B(pp)_2_]⋅DME (**1a**) and 1 M LiPF_6_ in EC:EMC electrolytes, XPS analysis was undertaken on the NMC811 cathode after the 500 cycles (Figure 8). Additionally, the XPS of the pristine NMC811 cathode is shown in Figure S4.2.

The C1s spectra reveal a noticeable decrease in the intensity of the C–C peak associated with conductive carbon (at 284.5 eV) and the PVDF binder (–CF_2_ at 291.0 eV, C–H at 286.0 eV) in Li[B(pp)_2_]⋅DME (**1a**),^[^ ^45^, ^46^ ^]^ suggesting the formation of a thicker CEI. The presence of –CF_3_ species (at 293.5 eV) may be attributed to residual **1a** salt on the electrodes and/or the inclusion of **1a** (or breakdown products of **1a**, containing a –CF_3_ group) in the CEI. Moreover, the O1s spectra indicates higher levels of –CO_3_ (at 531.8 eV) and C–O (at 533.2 eV) species in **1a** compared to 1 M LiPF_6_. Interestingly, lower intensity peaks in the F1s spectra, indicative of LiF (*ca*. 685.4 eV), are observed in **1a**, suggesting either negligible LiF formation or that this is covered by a thick layer containing –C–O and –CF_3_. The –C–F peak (at 688 eV) corresponding to the PVDF binder is evident in the F1s spectra. For the 1 M LiPF_6_ electrolyte, the P2p spectra shows POF and residual LiPF_6_ salt species at 134.9 and 137.1 eV, respectively. Lastly, in the Li1s/TM3p core levels region, the intensity of the Ni3p peak (at 69.8 eV) is observed to decrease in **1a**. This is consistent with the formation of a thicker CEI with the **1a**‐containing electrolyte, covering the NMC811 cathode surface. Overall, the thicker CEI (in part due to the decomposition of DME) with less LiF that is formed using 1 M **1a** electrolyte leads to the observed poorer capacity retention compared to using 1 M LiPF_6_ electrolyte.

To see if the lithium‐ion cycling performance of the [B(pp)_2_]^−^ anion could be improved, 0.2 M Li[B(pp)_2_] (**1b**, no DME solvation) in EC:EMC (3:7 v/v) was used as the electrolyte salt. Although the bulk conductivity is lower for 0.2 M **1b**, it was hypothesized the absence of DME may give more stable cycling.^[^ ^47^ ^]^ As before, coin cells were constructed using NMC811 and graphite as the active cathode and anode materials, respectively. Due to the lower bulk conductivity of 0.2 M **1b**, a slower cycling rate of C/3 was performed. For comparison, coin cells using 1 M Li[B(pp)_2_]⋅DME (**1a**) and 1 M LiPF_6_ in EC:EMC (3:7 v/v) electrolyte were also constructed and cycled at a C/3 rate.

**Figure 9:**
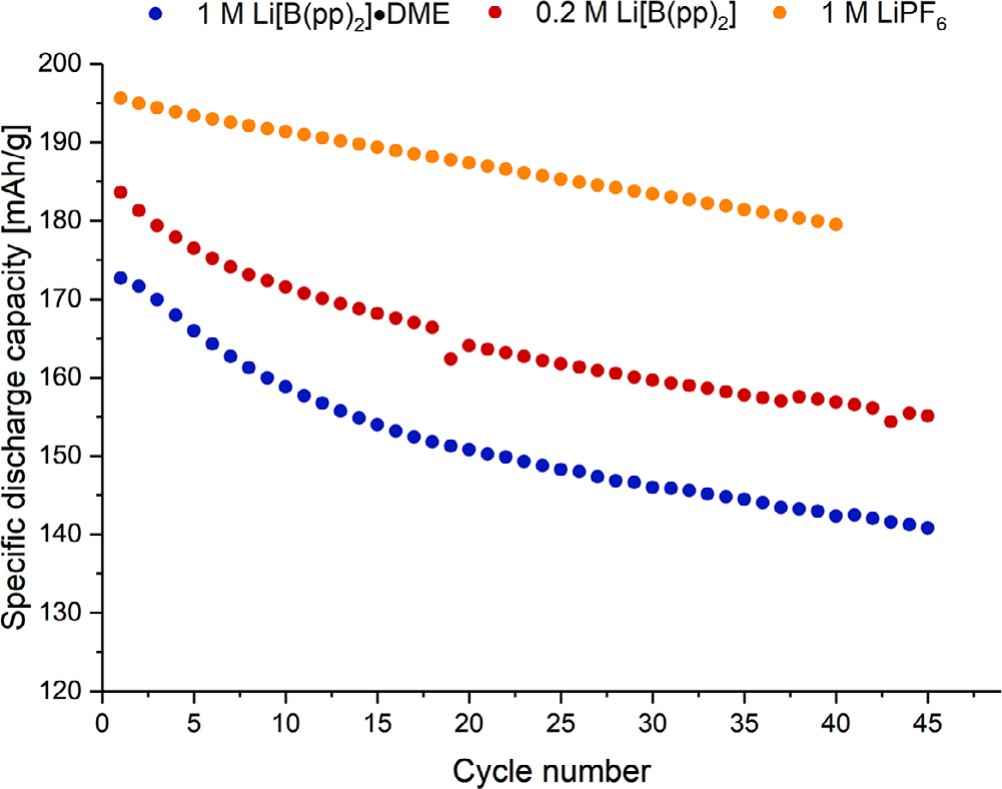
Discharge gravimetric capacity vs. cycle number for 1 M Li[B(pp)_2_]⋅DME (**1a**) EC:EMC (blue), 0.2 M Li[B(pp)_2_] (**1b**) EC:EMC (red), and 1 M LiPF_6_ EC:EMC (orange, LP57). Active electrode materials are NMC811 and graphite for the cathode and anode, respectively. Approximate constant current rate of C/3 for charge and discharge using cell voltage limits of 4.2 and 2.5 V. Measured in coin cells.

When using 0.2 M Li[B(pp)_2_] (**1b**) in EC:EMC (3:7 v/v), higher initial capacity and Coulombic efficiency (CE) were observed, compared to using 1 M Li[B(pp)_2_]⋅DME (**1a**) electrolyte (Figure 9 and S3.4.6). This finding is consistent with our observation made on the analogous sodium system.^[^ ^29^ ^]^ Although cells with **1a** cycled with lower capacity than **1b**, the capacity retention of both electrolytes was approximately the same after 45 cycles: 82% and 84% for **1a** and **1b**, respectively. Compared to 1 M LiPF_6_ electrolyte, the initial capacity after the formation cycles for 0.2 M **1b** was 6% lower, whereas for 1 M **1a** it was approximately 12% lower. The CEs for **1a** and **1b** began lower than for LiPF_6_ as dictated by the lower capacities. However, after approximately 18 cycles, the CEs stabilized and reached 98.3% and 99.0 % for **1a** and **1b**, respectively.

**Figure 10:**
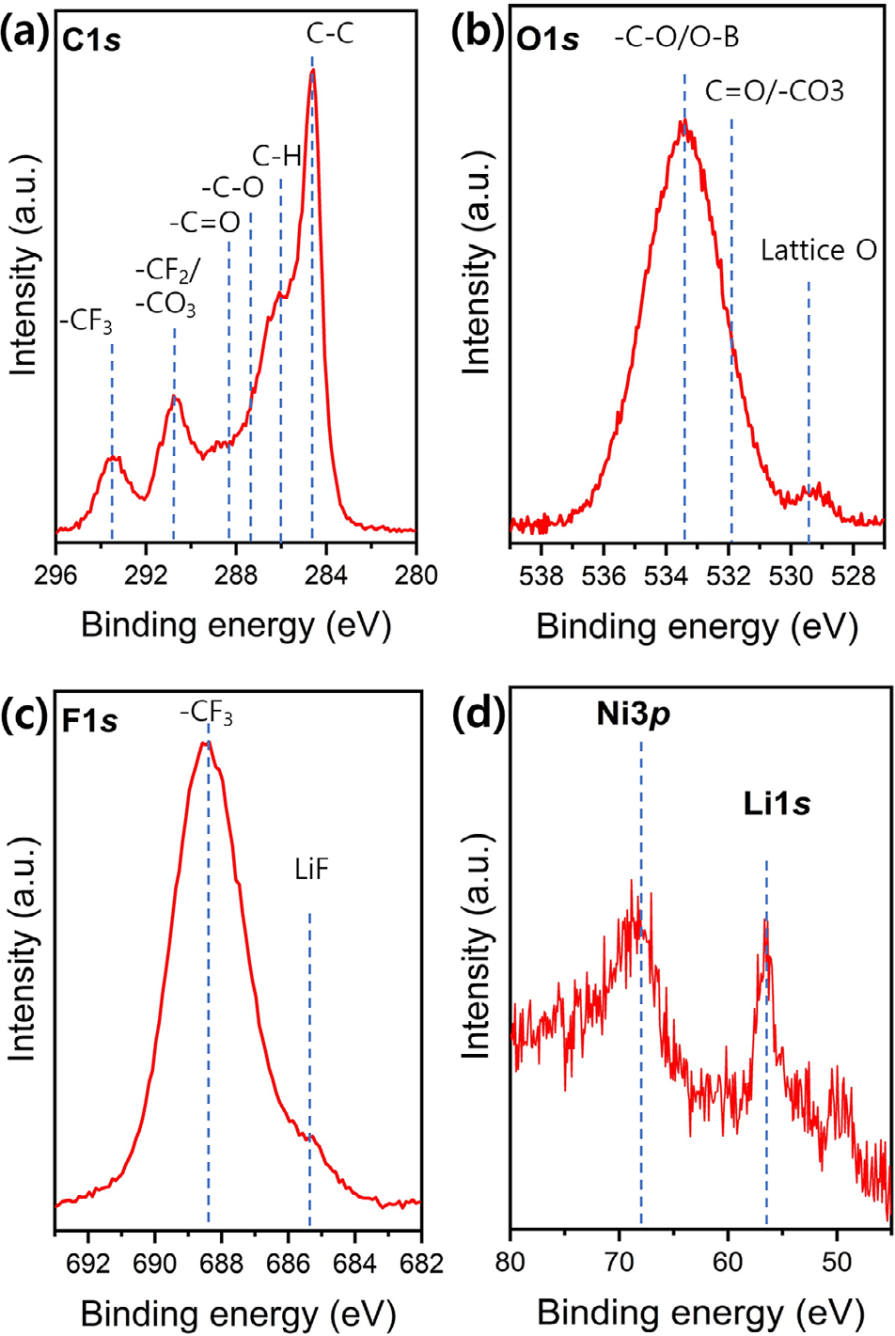
a) C1s, b) O1s, c) F1s, d) Ni3p/Li1s XPS spectra of the NMC‐811 cathode using 0.2 M Li[B(pp)_2_] (**1b**) in EC:EMC (3:7 v/v) electrolyte after 45 cycles.

Post‐cycling XPS on the NMC811 cathode was performed for cells containing 0.2 M Li[B(pp)_2_] (**1b**) to again gain insight into the CEI of the electrolyte. For **1b**, intense peaks for C–C (at 284.5 eV) and the PVDF binder (–CF_2_ at 291.0 eV, C–H at 285.8 eV) were found in the C1s spectra, along with a clearly visible peak of lattice oxygen (at 529.3 eV) in the O1s spectra. Additionally, an intense Ni3p peak along with the Li1s peak indicates the formation of a thinner CEI compared to Li[B(pp)_2_]⋅DME (**1a**) (Figure 10). However, this may in part be attributed to the fewer cycles performed for the 0.2 M **1b** electrolyte. Despite the fewer cycles, more LiF was found in the **1b** sample than in **1a**.

To investigate the electrolyte degradation when using lithium borate electrolytes further, solution‐state multinuclear NMR spectroscopy was performed. Coin cells, which were cycled at a C/3 rate, were opened in an inert atmosphere, and the electrolyte was extracted from the glass‐fiber separator using DMSO‐*d*_6_ as the NMR solvent. Electrolyte degradation of LiPF_6_‐containing electrolytes with NMC cathodes has previously been studied. These reports have shown that O_2_ is formed during cycling and reacts with EC to form CO_2_, CO, and H_2_O. The formation of water leads to secondary reactions and hydrolysis of the PF_6_^−^ anion.^[^ ^36^, ^48^, ^49^ ^]^ Interestingly, when preparing the NMR sample using 1 M Li[B(pp)_2_]⋅DME (**1a**), the DMSO‐*d*_6_ solution was yellow in color. This contrasts with the more transparent appearance of both the DMSO‐*d*_6_‐soaked separators using Li[B(pp)_2_] (**1b**) and LiPF_6_, suggesting the color is a consequence of DME solvent breakdown (Figure S6.1).

**Figure 11:**
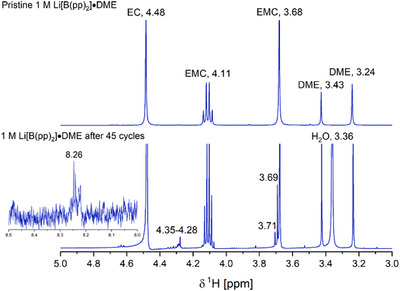
Top: ^1^H NMR (500 MHz, (CD_3_)_2_SO, 295 K) spectrum of pristine 1 M Li[B(pp)_2_]⋅DME (**1a**) in EC:EMC (3:7 v/v) electrolyte. Bottom: ^1^H NMR (500 MHz, (CD_3_)_2_SO, 295 K) spectrum of 1 M Li[B(pp)_2_]⋅DME (**1a**) in EC:EMC (3:7 v/v) electrolyte after cycling for 45 cycles.

From the ^1^H NMR spectrum of cycled 1 M Li[B(pp)_2_]⋅DME (**1a**), a new strong intensity singlet at 3.36 ppm is seen (Figures 11 and S5.3.5). This is ascribed to water. Water is also present in the DMSO‐*d*_6_ solvent; thus, it is not possible to quantify the amount of water formed during cycling accurately. By integrating the EMC solvent signals against EC, it is estimated that approximately 60% of EMC in the electrolyte was lost. This assumes that the EC content of the electrolyte is constant during cycling, but this may not be accurate if any EC is lost due to degradation (as is known to happen during SEI formation). Moreover, evaporation of EMC while preparing the NMR sample likely accounts for this large loss. The ^1^H NMR spectrum shows a series of new low‐intensity overlapping multiplets between 4.35 and 4.28 ppm, which may be assigned to the formation of polyethylene oxide‐based oligomers.^[^ ^49^ ^]^ Furthermore, it has previously been reported that lithium ethylene dicarbonate (LEDC), which forms from the reduction of EC solvent, gives rise to a singlet with a chemical shift of 4.29 ppm.^[^ ^50^ ^]^ Thus, the singlet at 4.28 ppm observed in this work may be assigned to LEDC.

**Scheme 3:**
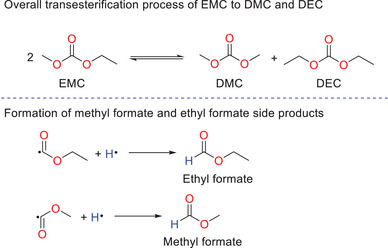
Top: transesterification of ethyl methyl carbonate (EMC) to dimethyl carbonate (DMC) and diethyl carbonate (DEC). Bottom: formation of methyl formate and ethyl formate side products, formed from recombination of methyl formate/ethyl formate radical with a hydrogen radical.

It is proposed that the new signal at 3.69 ppm is from the formation of dimethyl carbonate (DMC), which is known to form from the transesterification of EMC to give DMC and diethyl carbonate (DEC) as products (Scheme 3).^[^ ^51^, ^52^, ^53^ ^]^ Evidence of DEC formation was observed at 1.2 ppm. Further evidence of transesterification occurring was observed with the formation of ethyl formate, which is a known side product of the transesterification process and seen by low‐intensity signals at 8.26 ppm and 1.22 ppm.^[^ ^52^, ^53^ ^]^

By again integrating relative to the EC signal in the ^1^H NMR spectrum, the level of DME had reduced by 57% after the 45 cycles. Previously, lithium formate has been proposed as a decomposition product of DME in Li–O_2_ batteries,^[^ ^54^, ^55^ ^]^ which may also be assigned to the signal at 8.26 ppm. However, this formate signal appears in the post‐cycled ^1^H NMR spectrum of the unsolvated Li[B(pp)_2_] (**1b**) salt, where no DME is present. Moreover, the formation of formaldehyde from DME decomposition has also been proposed in Li–O_2_ batteries,^[^ ^54^, ^55^ ^]^ however, the ^1^H NMR spectrum does not show evidence of formaldehyde formation (expected at 9.58 ppm).^[^ ^35^ ^]^

**Figure 12:**
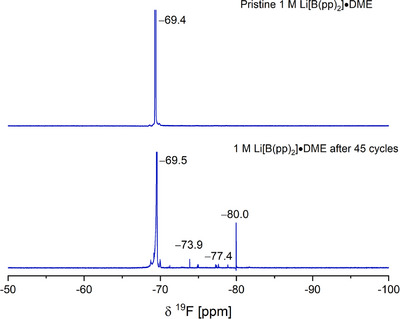
Top: ^19^F NMR (471 MHz, (CD_3_)_2_SO, 295 K) spectrum of pristine 1 M Li[B(pp)_2_]⋅DME (**1a**) EC:EMC (3:7 v/v) electrolyte. Bottom: ^19^F NMR (471 MHz, (CD_3_)_2_SO, 295 K) spectrum of 1 M Li[B(pp)_2_]⋅DME (**1a**) EC:EMC (3:7 v/v) electrolyte after cycling for 45 cycles.

The ^19^F NMR spectrum of cycled 1 M Li[B(pp)_2_]⋅DME (**1a**) shows the formation of new signals. Multiple low‐intensity signals between −71.3 ppm and −80.0 ppm in the ^19^F NMR spectrum were seen (Figures 12 and S5.3.7); these signals were not present in the pristine electrolyte and indicate partial decomposition of the anion. By integrating relative to the [B(pp)_2_]^−^ anion signal, these new resonances make up approximately 1% of the sample. The chemical shifts of these new signals are consistent with the presence of –CF_3_ groups. The ^11^B NMR spectrum shows no change compared to the pristine electrolyte (Figure S5.3.6); the relatively low sensitivity of ^11^B NMR spectroscopy is a likely reason for this.

Similar findings to Li[B(pp)_2_]⋅DME (**1a**) in EC:EMC electrolyte were observed in the degradation analysis of Li[B(pp)_2_] (**1b**)‐containing electrolyte (Figures S5.3.9–S5.3.12). In the ^1^H NMR spectrum, there was a 40% reduction in the EMC integral values and the presence of a strong water signal. A series of low‐intensity overlapping multiplets indicating polyethylene oxide‐based oligomers and the singlet at 8.25 ppm assigned to ethyl formate were again found. The ^19^F NMR spectrum post cycling showed new low‐intensity signals between −71.2 ppm and −80 ppm, indicating partial degradation of the Li[B(pp)_2_] salt, while the ^11^B NMR spectrum remained unchanged.

Lastly, as 0.2 M Li[B(pp)_2_] (**1b**) in EC:EMC (3:7 v/v) was used as an electrolyte, a 0.2 M LiPF_6_ in EC:EMC (3:7 v/v) solution was prepared and used as an electrolyte for a more direct comparison. Cells were again constructed using NMC811 and graphite electrodes and cycled at rate C/3. The 0.2 M LiPF_6_ in EC:EMC (3:7 v/v) cells cycled at lower initial capacity compared to the 1 M LiPF_6_ cells and is comparable to the 0.2 M **1b**‐containing electrolyte (Figure S3.4.7). However, during the course of 33 cycles, the capacity retention of the 0.2 M LiPF_6_ EC:EMC (3:7 v/v) electrolyte was higher than the 0.2 M **1b**‐containing electrolyte (96% and 86%, respectively).

## 3 Conclusion

In summary, the synthesis of Li[B(pp)_2_]⋅DME (**1a**) and Li[B(pp)_2_] (**1b**) has been reported, and their application as electrolyte salts for lithium‐ion batteries has been investigated. The lithium borate salts are prepared from the addition of lithium borohydride with perfluoropinacol, using either DME (for **1a**) or toluene (for **1b**) as the reaction solvents. Both **1a** and **1b** exhibit excellent air stability, with no decomposition of the [B(pp)_2_]^−^ anion detected after 5 weeks. Moreover, the salts have high thermal decomposition temperatures of 211°C and 241°C for **1a** and **1b**, respectively, which may allow for higher temperature battery cycling. Both these factors offer significant improvements on the conventionally used LiPF_6_ electrolyte salt. For galvanostatic cycling experiments, both **1a** and **1b** salts underwent cycling, where **1b** gave higher capacities than **1a**. Compared to using LiPF_6_, greater cell polarization occurred for **1a** and XPS analysis on the cycled NMC811 cathode found a thicker CEI with less LiF present. Nevertheless, the superior air tolerance and thermal stability of the lithium borate salts may help realize a more logistically convenient (handling, transport, and storage) electrolyte.

## 4 Experimental Section

Unless stated otherwise, all reactions were carried out under an atmosphere of dinitrogen using standard Schlenk and glove box (under argon; Saffron, Alpha model) techniques. 1,2‐dimethoxyethane (DME) solvent was dried over 4 Å activated molecular sieves for 24 hours and stored in an ampoule fitted with a Teflon valve under a dinitrogen atmosphere. Toluene and diethyl ether were collected freshly distilled over sodium‐potassium amalgam before use. Deuterated solvents were dried over 4 Å activated molecular sieves and stored in an argon‐filled glovebox. Perfluoropinacol was purchased from Fluorochem. Perfluoropinacol was heated to 60°C and dried over 4 Å activated molecular sieves, then stored over these molecular sieves in an ampoule fitted with a Teflon valve under a dinitrogen atmosphere. Lithium borohydride was purchased from Sigma‐Aldrich, crystallized from diethyl ether, and dried at 90°C in vacuo (1 × 10^−2^ mbar) for two hours. Ethylene carbonate: ethyl methyl carbonate (EC:EMC 3:7 v/v) was purchased from Solvionic and dried over 4 Å activated molecular sieves for 24 hours before use.

^1^H, ^13^C{^1^H}, ^19^F, ^11^B, ^31^P, and ^7^Li solution‐state NMR spectra were recorded at 298.0 K on a Bruker 400 MHz AVIII HD Smart Probe spectrometer. Chemical shifts are expressed as parts per million (ppm, δ) and are referenced to CD_3_CN (1.95/118.26 ppm) and (CD_3_)_2_SO (2.50/39.52 ppm) as internal standards. Multinuclear NMR spectra were referenced to BF_3_·Et_2_O/CDCl_3_ (^11^B), H_3_PO_4_ (^31^P), CFCl_3_ (^19^F) and 1 M LiCl in D_2_O (^7^Li) The description of signals includes s = singlet, d = doublet, t = triplet, q = quartet, q = quintet, and m = multiplet. All coupling constants are absolute values and are expressed in Hertz (Hz). High‐resolution mass spectra (HRMS) were collected by the School of Chemistry at the University of Cambridge using a Waters Xevo G2‐S QTOF mass spectrometer in negative mode. Elemental analysis for carbon, hydrogen, and nitrogen was performed using a PerkinElmer 240 Elemental Analyzer.

### Synthesis of Lithium Borate Salts

#### Synthesis of lithium bis(perfluorinated pinacolato)borate, Li[B(pp)_2_]·*DME* (**1a**)

A Schlenk tube was charged with purified lithium borohydride (106 mg, 4.87 mmol, 1.0 equiv) and 1,2‐dimethoxyethane (DME, 30 mL). The solution was cooled to 0°C, and hexafluoro‐2,3‐bis(trifluoromethyl)‐2,3‐butanediol (3.58 g, 10.7 mmol, 2.2 equiv) was added dropwise. The evolution of dihydrogen was immediately observed with effervescence. The reaction was left to stir at 0°C for 30 minutes before slowly warming to room temperature. The solution was then heated to 50°C and left to stir for 18 hours. After heating, the reaction was left to cool to ambient temperature; the solvent level was then reduced to approximately one‐third *in vacuo*, and pentane (40 mL) was added, which precipitated the product out. The resulting white powder was washed with pentane (3 × 10 mL) and dried *in vacuo* at 90°C to give the title product, lithium bis(perfluorinated pinacolato)borate, as a DME solvated adduct, Li[B(pp)_2_]·DME. Yield: 1.94 g, 2.51 mmol, 52%.

**Caution: hexafluoro‐2,3‐bis(trifluoromethyl)‐2,3‐butanediol is fatal in contact with skin. Given the toxicity of the alcohol, the salt should be handled as being harmful and toxic. Degrading the salt to liberate the ligand should not be attempted**.

**^1^H NMR** (400 MHz, CD_3_CN, 295 K) δ/ppm: 3.47 (s, 4H, DME–CH_2_), 3.30 (s, 6H, DME–CH_3_). **^13^C{^1^H} NMR** (101 MHz, (CD_3_)_2_SO, 295 K) δ/ppm: 125.9–117.1 (m, CF_3_), 85.1 (br m, OC(CF_3_)_2_), 71.1 (s, DME–CH_2_), 58.0 (s, DME–CH_3_). **^11^B NMR** (128 MHz, CD_3_CN, 295 K) δ/ppm: 11.4 (s). **^19^F NMR** (376 MHz, CD_3_CN, 295 K) δ/ppm: −70.4 (s). **^7^Li NMR** (155 MHz, CD_3_CN, 295 K) δ/ppm: −2.4 (s). **HRMS** (ASAP^−^) m/z calculated for [M]^−^ [C_12_BO_4_F_24_]^−^: 674.9506 found: 674.9486. Anal. Calcd for C_16_H_10_BF_24_LiO_6_ (Li[B(pp)_2_]·DME): C, 24.9; H, 1.3. Found: C, 24.9; H, 1.3.

#### Synthesis of unsolvated lithium bis(perfluorinated pinacolato)borate, Li[B(pp)_2_] (**1b**)

A Schlenk tube was charged with purified lithium borohydride (318 mg, 14.6 mmol, 1.0 equiv) and toluene (30 mL). The solution was cooled to 0°C, and hexafluoro‐2,3‐bis(trifluoromethyl)‐2,3‐butanediol (10.2 g, 30.7 mmol, 2.1 equiv) was added dropwise. The evolution of dihydrogen was observed with effervescence. The reaction was left to stir at 0°C for 30 minutes before slowly warming to room temperature. The solution was then heated to 90°C and left to stir for 18 hours. During this time the product salt began to precipitate out of solution. After heating, the reaction was left to cool to ambient temperature, and pentane (40 mL) was added, which further precipitated the product out. The solvent was removed by syringe, and the white powder was washed with pentane (3 × 10 mL) and dried *in vacuo* at 90°C to give the title product, lithium bis(perfluorinated pinacolato)borate, Li[B(pp)_2_]. Yield: 3.35 g, 4.34 mmol, 30%.

**Caution: hexafluoro‐2,3‐bis(trifluoromethyl)‐2,3‐butanediol is fatal in contact with skin. Given the toxicity of the alcohol, the salt should be handled as being harmful and toxic. Degrading the salt to liberate the ligand should not be attempted**.

**^13^C{^1^H} NMR** (101 MHz, (CD_3_)_2_SO, 295 K) δ/ppm: 125.9–117.1 (m, CF_3_). NB: OC(CF_3_)_2_ not observed. **^11^B NMR** (128 MHz, (CD_3_)_2_SO, 295 K) δ/ppm: 11.4 (s). **^19^F NMR** (376 MHz, (CD_3_)_2_SO, 295 K) δ/ppm: −69.3 (s). **^7^Li NMR** (155 MHz, (CD_3_)_2_SO, 295 K) δ/ppm: −0.9 (s). **HRMS** (ASAP^−^) *m/z* calculated for [M]^−^ [C_12_BO_4_F_24_]^−^: 674.9506 found: 674.9532. **Anal**. Calcd for C_12_BF_24_LiO_4_ (Li[B(pp)_2_]): C, 21.1; H, 0.0. Found: C, 21.1; H, 0.3.

Electrolyte solutions were prepared by dissolving the lithium borate salt Li[B(pp)_2_]·DME or Li[B(pp)_2_] in an organic solvent blend of ethylene carbonate and ethyl methyl carbonate (EC:EMC 3:7 v/v). The water content of the electrolytes was determined via Metrohm 899 Coulometer Karl Fischer titration and found to be approximately 40 ppm. Air‐sensitive techniques were used throughout the synthesis of the salts to minimize water content. EC:EMC solvent was dried over 4 Å activated molecular sieves before the salt was added.

The studied electrolyte electrochemical stability window (ESW) was determined using cyclic voltammetry (CV). Electrolytes were tested in a three‐electrode cell (RHD, surface cell). Lithium metal was used as the counter and pseudo‐reference electrode. The RHD surface cell is a “beaker style” cell that uses an excess of electrolyte (650 µl) and does not use a separator. For the aluminim working electrode, three consecutive CV scans were carried out from open circuit voltage to 4.8 V at a scan rate of 5 mV/sec. For the copper working electrode, three consecutive CV scans were carried out from open circuit voltage to 0.05 V at a scan rate of 5 mV/sec.

Coin cells (2032 from Cambridge Energy Solutions) were prepared in an argon glovebox (O_2_ < 1 ppm, H_2_O < 1 ppm). For Li‐ion battery cells, 2‐electrode single‐layer coin cells with a 1.77 cm^2^ cathode area were assembled with a geometrically oversized hard carbon anode (2.01 cm^2^). LiNi_0.8_Mn_0.1_Co_0.1_O_2_ (NMC811) and graphite‐printed electrode foils were provided by the Cell Analysis, Modeling, and Prototype (CAMP) facility at Argonne National Lab (USA). The NMC cathode (batch code A‐C020) consisted of 90 wt% NMC811 (Targray), 5 wt% PVDF binder (Solvay 5130), and 5 wt% carbon black (Timcal C45) coated onto an aluminim current collector. This had a mass loading of active material of 8.3 mg cm^−2^ and a practical capacity of 195 mAhg^−1^. The graphite electrode consisted of 91.83 wt% graphite powder (Hitachi MagE3), 2 wt% carbon black (Timcal C45), 6 wt% PVDF binder (Kureha 9300), and 0.17 wt% oxalic acid coated onto a copper current collector. This had a mass loading of 5.8 mg cm^−2^ and a practical capacity of 360 mAhg^−1^.^[^ ^42^, ^56^ ^]^

The long‐term cycle life battery tests of Li[B(pp)_2_]·DME and LP57 electrolytes were performed at room temperature with a battery cycler (LBT 20084, Arbin Instruments). The formation protocol consisted of being held at 1.5 V for 3 hours, then one charge‐discharge cycle at a rate of C/20 between a voltage limit of 4.2–2.5 V. For cycle life tests, the cell was cycled between 2.5 V and 4.2 V with a 1C charge/discharge rate. The C‐rate was based on a nominal specific capacity of 195 mAhg^−1^ for the cathode. Celgard 3500 polyethylene‐polypropylene, which was dried under vacuum (1 × 10^−2^ mbar) at 40 °C for 20 hours, was used as the separator with 50 µL of electrolyte. Potentiostatic electrochemical impedance spectroscopy (PEIS) measurements were done using Biologic Instruments VSP‐300 in the frequency range 1 MHz–0.01 Hz at 5 mV amplitude.

For electrochemical battery tests using Li[B(pp)_2_]·DME, Li[B(pp)_2_] and LP57 electrolytes for 45 cycles, Li‐ion coin cells were tested at room temperature with a battery cycler (MPG 200, Biologic Instruments). The formation protocol consisted of 2 charge‐discharge cycles at a rate of C/3 between a voltage limit of 4.2–2.5 V. For cycle life tests, a C/3 charge/discharge (constant current‐constant voltage, CCCV profile) rate was used. The C‐rate was based on a nominal specific capacity of 195 mAhg^−1^ for the cathode. Glass fiber, which was dried under vacuum (1 × 10^−2^ mbar) at 100 °C for 18 hours, was used as the separator, with 100 µL of electrolyte.

The electrochemically cycled NMC and graphite electrodes underwent rinsing with dimethyl carbonate solvent and were subsequently dried for X‐ray Photoelectron Spectroscopy (XPS) analysis. All samples were transferred to the XPS measurement chamber via an inert transfer vessel and analyzed using a Phi XPS Versaprobe III with an Al Kα X‐ray source at 1486 eV (probe depth < 10 nm) under high vacuum conditions (>10^−6^ mbar). The XPS spectra were energy calibrated by setting the C‐C peak to 284.5 eV and analyzed using CASA‐XPS software with Shirley background subtraction.

## Conflict of Interest

The authors declare no conflict of interest.

## Supporting information



Supporting Information

Supporting Information

## Data Availability

The data that support the findings of this study are available in the supplementary material of this article.
