# Supplementary material for: Synthesis and Electrolyte Study of Lithium Bis(perfluorinated pinacolato)Borate for Lithium‐Ion Batteries
Source: Chemistry. 2025 Nov 19;31(71):e02653. doi: 10.1002/chem.202502653 (PMC12734670; doi:10.1002/chem.202502653)
Supplement: Supplementary file 1 — Supporting Information [file CHEM-31-e02653-s002.docx]

Supplementary Information for:

**Synthesis and Electrolyte Study of Lithium Bis(perfluorinated pinacolato)borate for Lithium-Ion Batteries**

Darren M. C. Ould,^[a,c]^ Zachary Ruff,^[a,c]^ Megan E. Penrod,^[a,c]^ Pravin N. Didwal,^[b,c]^ Timothy Weiss,^[a]^ Kieran Mylrea,^[a,c]^ Holly E. Smith,^[a]^ Andrew D. Bond,^[a]^ Robert S. Weatherup,^[b,c]^ Clare P. Grey*^[a,c]^ and Dominic S. Wright*^[a,c]^

[a] Yusuf Hamied Department of Chemistry, University of Cambridge, Lensfield Road, Cambridge, CB2 1EW, U.K.

[b] Department of Materials, University of Oxford, Oxford, OX1 3PH, U.K.

[c] The Faraday Institution, Quad One, Harwell Science and Innovation Campus, Didcot, OX11 0RA, U.K.

Contents

[**S1 Thermal measurements.** 2](#_Toc213230530)

[**S2 Single crystal X-ray diffraction.** 3](#_Toc213230531)

[**S3 Electrochemistry measurements.** 6](#_Toc213230532)

[**S4 X-ray photoelectron spectroscopy (XPS) measurements** 16](#_Toc213230533)

[**S5 NMR spectra.** 18](#_Toc213230534)

[**S6 Photo of NMR solutions of Li-ion cycled electrolyte.** 59](#_Toc213230535)

# **S1 Thermal measurements.**

**S1.1 Thermal stability general experimental.**

Thermogravimetric analysis (TGA): TGA data was recorded with a Mettler Toledo TGA / DSC 2 Star^ed^ system equipped with a Huber minichiller. A few milligrams of sample were taken out of the argon‑filled glovebox and immediately transferred to the TGA heating chamber to minimise air exposure. All the measurements were performed form 25 °C to 600 °C with a heating rate of 10 °C min^‑1^ and under nitrogen flow.

**S1.2 Thermal stability plots.**


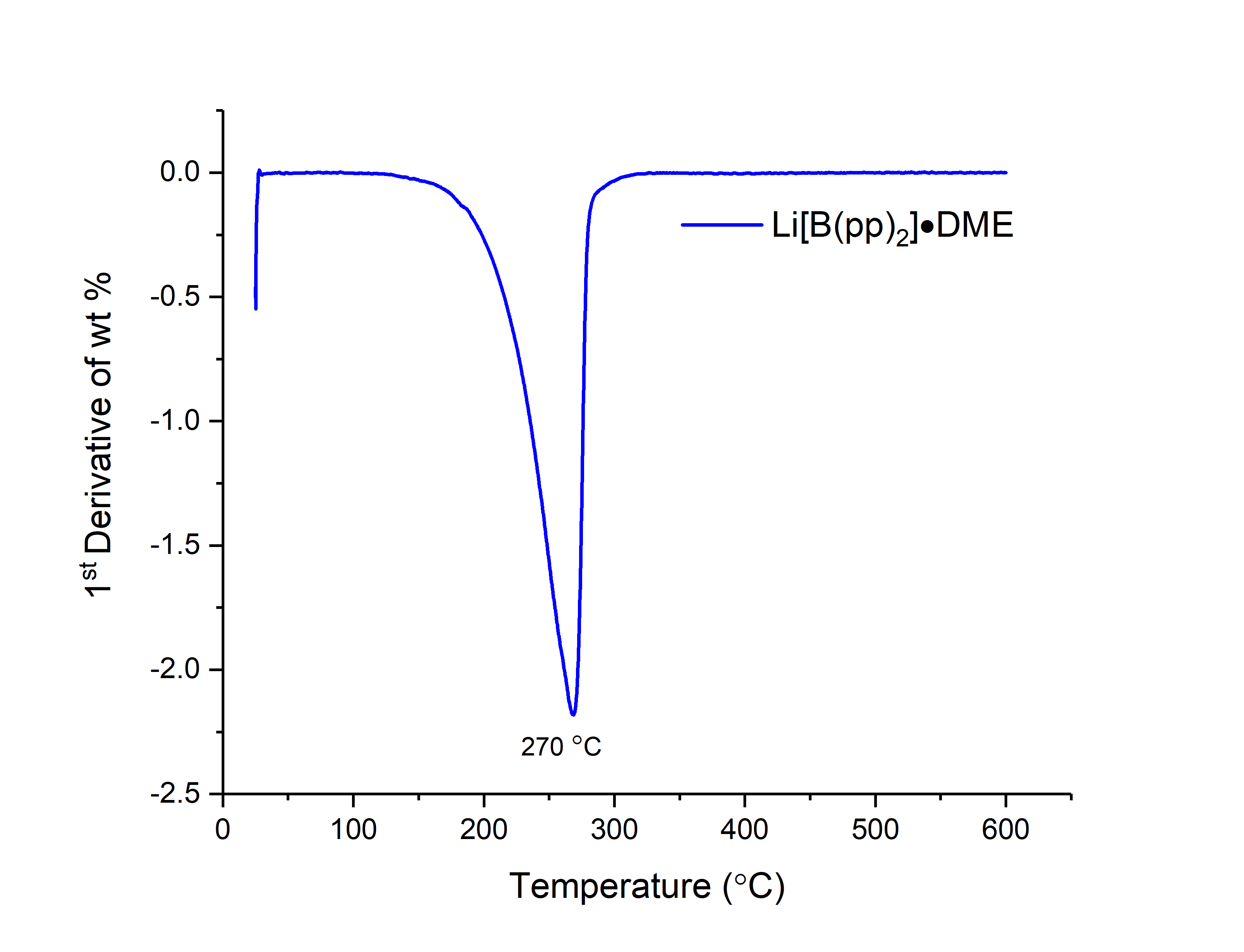


**Figure S1.1** 1^st^ derivative of the Weight *vs*. Temperature plot for Li[B(pp)_2_]·DME electrolyte salt, showing the inflection point temperature.


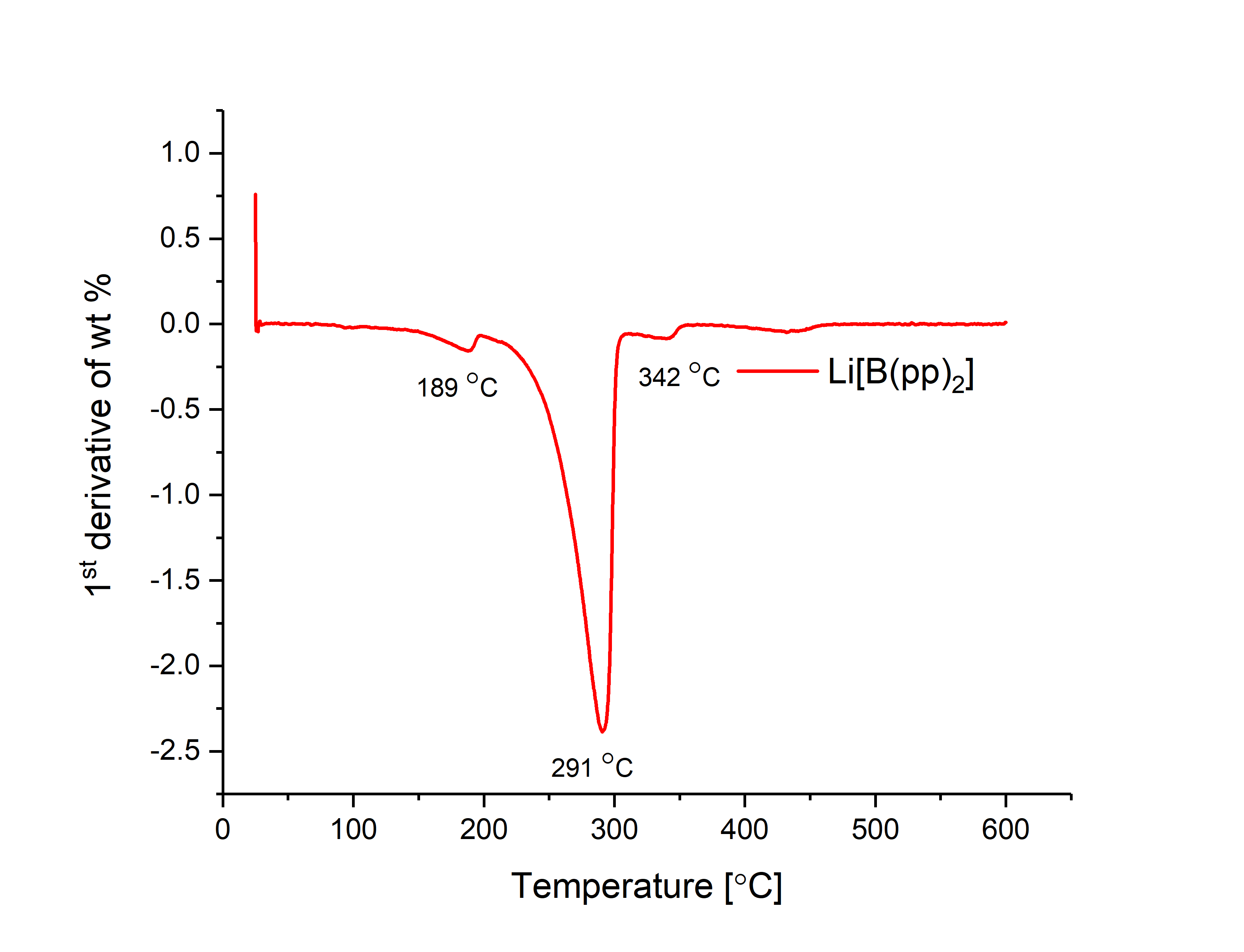


**Figure S1.2** 1^st^ derivative of the Weight *vs*. Temperature plot for Li[B(pp)_2_] electrolyte salt, showing the inflection point temperature.

# **S2 Single crystal X-ray diffraction.**

**S2.1 Single crystal X-ray diffraction experimental.**

X-ray data were collected on a Bruker D8-QUEST diffractometer, equipped with an Incoatec IμS Cu microsource (λ = 1.5418 Å) and a PHOTON-III detector operating in shutterless mode. The crystal was mounted on a MiTeGen crystal mount using inert polyfluoroether oil and the analysis was carried out under an Oxford Cryosystems open-flow N_2_ Cryostream operating at 180(2) K. The control and processing software was Bruker *APEX4*. The diffraction images were integrated using *SAINT* in *APEX4*, and a multi-scan correction was applied using *SADABS*. The final unit-cell parameters were refined against all reflections. Structures were solved using *SHELXT* and refined using *SHELXL*.

|  | **Li[B(pp)_2_]⋅DME** |
| --- | --- |
| CCDC number | 2358258 |
| Cambridge data number | DW_B1_0474 |
| Chemical formula | C_16_H_10_BF_24_LiO_6_ |
| Formula weight | 771.99 |
| Temperature / K | 180(2) |
| Crystal system | monoclinic |
| Space group | P2_1_/n |
| a / Å | 9.7753(4) |
| b / Å | 17.1666(6) |
| c / Å | 16.2536(6) |
| α / ° | 90 |
| β / ° | 96.670(2) |
| γ / ° | 90 |
| Unit-cell volume / Å^3^ | 2709.03(18) |
| Z | 4 |
| Calc. density / g cm^–3^ | 1.893 |
| F(000) | 1512 |
| Radiation type | Cu Kα |
| Absorption coefficient / mm^–1^ | 2.254 |
| Crystal size / mm^3^ | 0.30 x 0.22 x 0.08 |
| 2-θ range / ° | 10.31-133.33 |
| Completeness to max 2-θ | 0.993 |
| No. of reflections measured | 56602 |
| No. of independent reflections | 4763 |
| R(int) | 0.0322 |
| No. parameters / restraints | 446 / 3 |
| Final R1 values (I > 2σ(I)) | 0.0367 |
| Final wR(F^2^) values (all data) | 0.0889 |
| Goodness-of-fit on F^2^ | 1.051 |
| Largest difference peak & hole / e Å^–3^ | 0.531, -0.376 |

**S2.2 Crystal structure of Li[B(pp)_2_]⋅DME.**


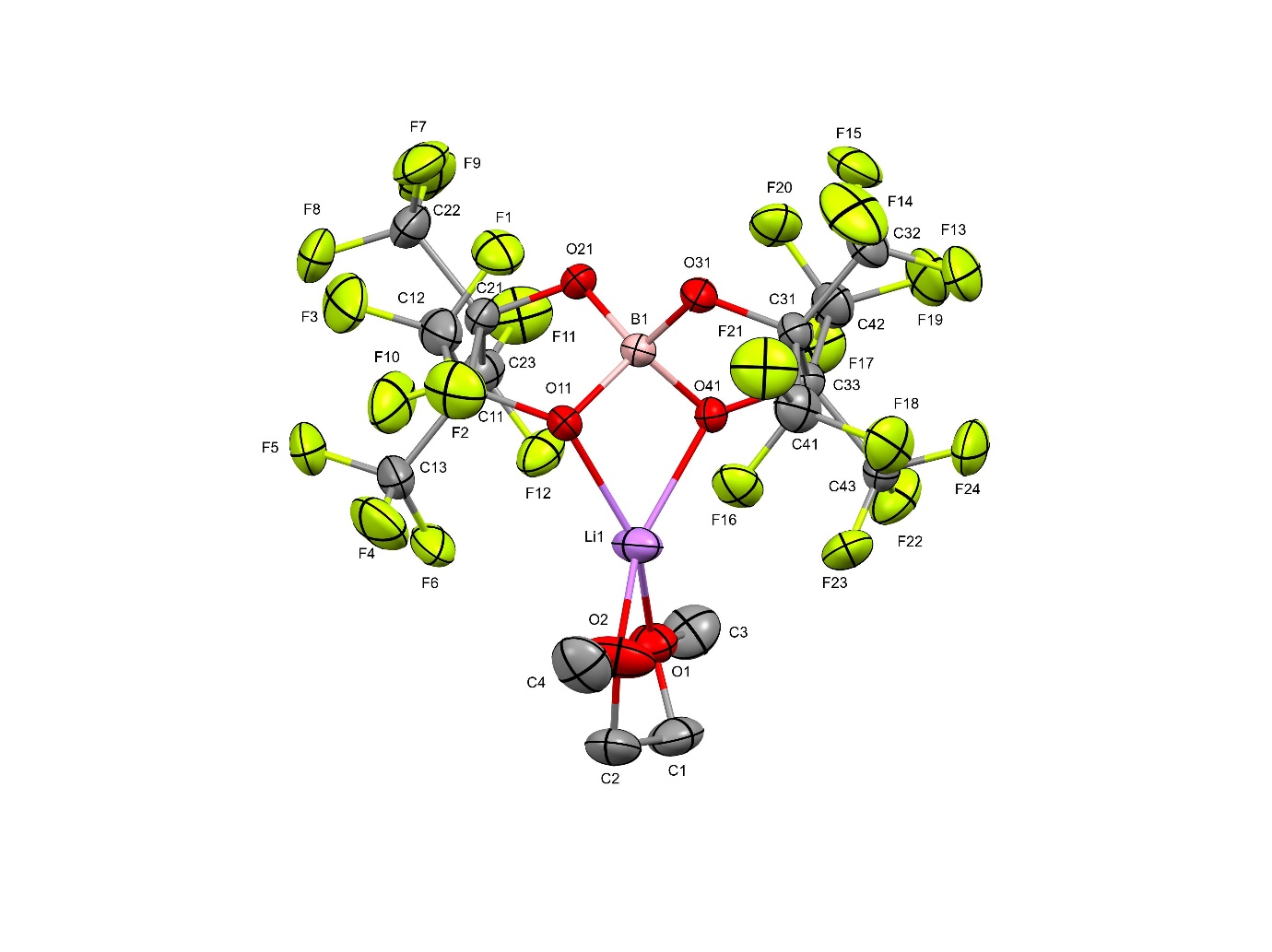


**Figure S2.2.1** Molecular structure of Li[B(pp)_2_]⋅DME. Pink: B, red: O, green: F, lilac: Li. Displacement ellipsoids are drawn at 50% probability and H atoms are omitted. A minor disorder component for one CH_2_ group in the DME ligand is also omitted for clarity.

**Selected bond distances (Å)**

| B(1)–O(11) | 1.493(2) |  | Li(1)–O(1) | 1.933(3) |  | C(11)–O(11) | 1.3944(19) |  | O(1)–C(1) | 1.390(3) |
| --- | --- | --- | --- | --- | --- | --- | --- | --- | --- | --- |
| B(1)–O(21) | 1.450(2) |  | Li(1)–O(2) | 1.910(3) |  | C(21)–O(21) | 1.385(2) |  | O(1)–C(3) | 1.420(3) |
| B(1)–O(31) | 1.452(2) |  | Li(1)–O(11) | 2.027(3) |  | C(31)–O(31) | 1.3877(19) |  | O(2)–C(2) | 1.401(3) |
| B(1)–O(41) | 1.489(2) |  | Li(1)–O(41) | 2.095(3) |  | C(41)–O(41) | 1.3970(18) |  | O(2)–C(4) | 1.434(3) |
|  |  |  |  |  |  |  |  |  | C(1)–C(2) | 1.545(5) |
|  |  |  |  |  |  |  |  |  |  |  |
| C(11)–C(12) | 1.564(3) |  | C(12)–F(1) | 1.328(2) |  | C(23)–F(10) | 1.327(2) |  | C(42)–F(19) | 1.333(2) |
| C(11)–C(13) | 1.555(2) |  | C(12)–F(2) | 1.325(3) |  | C(23)–F(11) | 1.319(2) |  | C(42)–F(20) | 1.322(2) |
| C(11)–C(21) | 1.611(2) |  | C(12)–F(3) | 1.336(2) |  | C(23)–F(12) | 1.336(2) |  | C(42)–F(21) | 1.328(2) |
| C(21)–C(22) | 1.557(2) |  | C(13)–F(4) | 1.326(3) |  | C(32)–F(13) | 1.323(2) |  | C(43)–F(22) | 1.327(2) |
| C(21)–C(23) | 1.569(3) |  | C(13)–F(5) | 1.323(2) |  | C(32)–F(14) | 1.321(3) |  | C(43)–F(23) | 1.323(2) |
| C(31)–C(32) | 1.557(2) |  | C(13)–F(6) | 1.319(2) |  | C(32)–F(15) | 1.316(2) |  | C(43)–F(24) | 1.322(2) |
| C(31)–C(33) | 1.564(2) |  | C(22)–F(7) | 1.320(2) |  | C(33)–F(16) | 1.336(2) |  |  |  |
| C(31)–C(41) | 1.610(2) |  | C(22)–F(8) | 1.325(2) |  | C(33)–F(17) | 1.320(2) |  |  |  |
| C(41)–C(43) | 1.552(2) |  | C(22)–F(9) | 1.324(3) |  | C(33)–F(18) | 1.334(2) |  |  |  |
| C(41)–C(42) | 1.562(2) |  |  |  |  |  |  |  |  |  |

**Selected bond angles (°)**

| O(21)–B(1)–O(31) | 109.60(12) |  | O(2)–Li(1)-O(1) | 84.50(13) |
| --- | --- | --- | --- | --- |
| O(21)–B(1)–O(41) | 118.68(13) |  | O(2)–Li(1)-O(11) | 111.89(16) |
| O(31)–B(1)–O(41) | 104.02(12) |  | O(1)–Li(1)-O(11) | 147.8(2) |
| O(21)–B(1)–O(11) | 103.85(12) |  | O(2)–Li(1)-O(41) | 152.7(2) |
| O(31)–B(1)–O(11) | 118.30(13) |  | O(1)–Li(1)-O(41) | 109.26(15) |
| O(41)–B(1)–O(11) | 102.98(12) |  | O(11)–Li(1)-O(41) | 68.94(10) |
|  |  |  |  |  |
| B(1)–O(11)–Li(1) | 95.32(12) |  | C(11)–O(11)–Li(1) | 132.81(14) |
| B(1)–O(41)–Li(1) | 92.67(11) |  | C(41)–O(41)–Li(1) | 131.48(13) |
| C(11)–O(11)–B(1) | 110.12(12) |  | C(1)–O(1)–Li(1) | 111.15(18) |
| C(21)–O(21)–B(1) | 111.40(12) |  | C(3)–O(1)–Li(1) | 129.05(16) |
| C(31)–O(31)–B(1) | 111.24(12) |  | C(2)–O(2)–Li(1) | 112.49(16) |
| C(41)–O(41)–B(1) | 109.74(11) |  | C(4)–O(2)–Li(1) | 131.67(17) |

# **S3 Electrochemistry measurements.**

**S3.1 Conductivity measurement experimental.**

Solution conductivity measurements were made in a TSC 70 Closed cell from RHD instruments. 70 µl of each liquid was filled into the cell and sealed inside an argon-filled glovebox at approximately 25°C. Impedance spectra were measured using a PalmSens4 or Biologic potentiostat, with an applied voltage amplitude of 10 mV and frequencies between 1 MHz and 1 Hz. The impedance spectra were fitted using the equivalence circuit R+Q, and the solution conductivity was found by taking the reciprocal of the R component, multiplied by the cell constant.

The cell constant was determined using a 1413 µS cm^-1^ conductivity standard solution from Hanna Instruments. 70 µl of the solution was filled into the cell, which was sealed and placed in an incubator held at 25°C. An impedance spectrum was measured as above. The spectrum was fitted with a Q+R/Q circuit and the measured resistance was multiplied by 1413 µS cm^-1^ to determine the cell constant, which was found to be 6.1 ± 0.1cm^‑1^.

**S3.2 Cyclic voltammetry measurements of the studied electrolytes.**


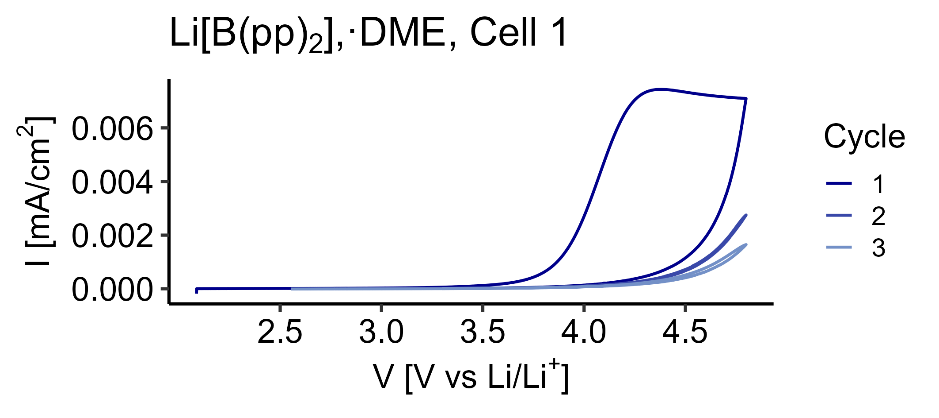


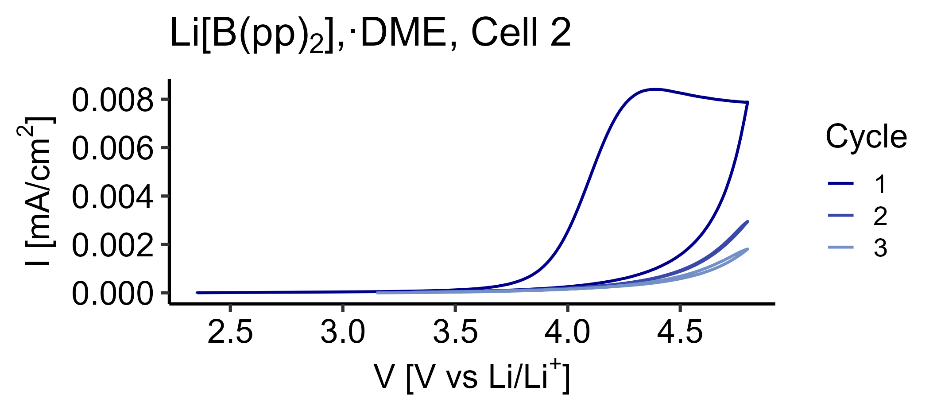


**Figure S3.2.1** Cyclic voltammetry of 1 M Li[B(pp)_2_]⋅DME in EC:EMC (3:7 v/v) electrolyte measured in three-electrode cell at scan rate 5 mV s^−1^. Working electrode: aluminium, counter electrode: lithium, reference electrode: lithium.


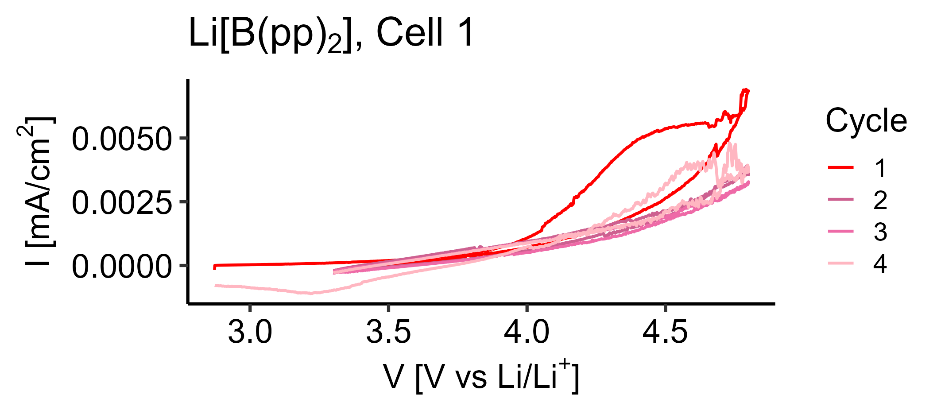


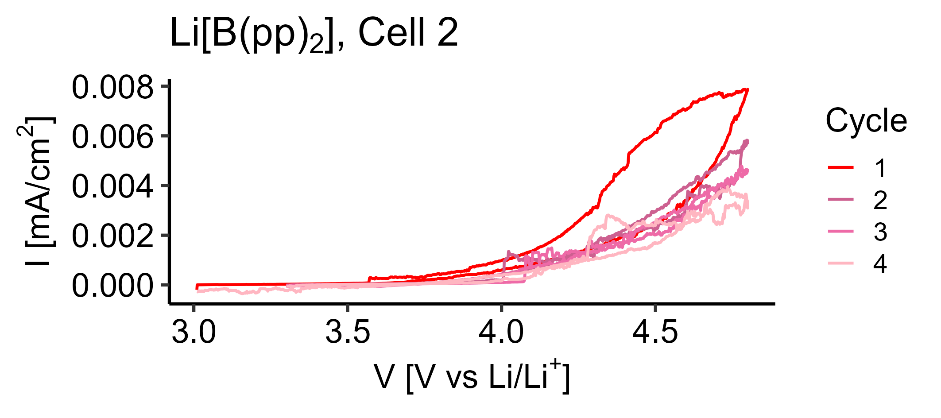


**Figure S3.2.2** Cyclic voltammetry of 0.1 M Li[B(pp)_2_] in EC:EMC (3:7 v/v) electrolyte measured in three-electrode cell at scan rate 5 mV s^−1^. Working electrode: aluminium, counter electrode: lithium, reference electrode: lithium.


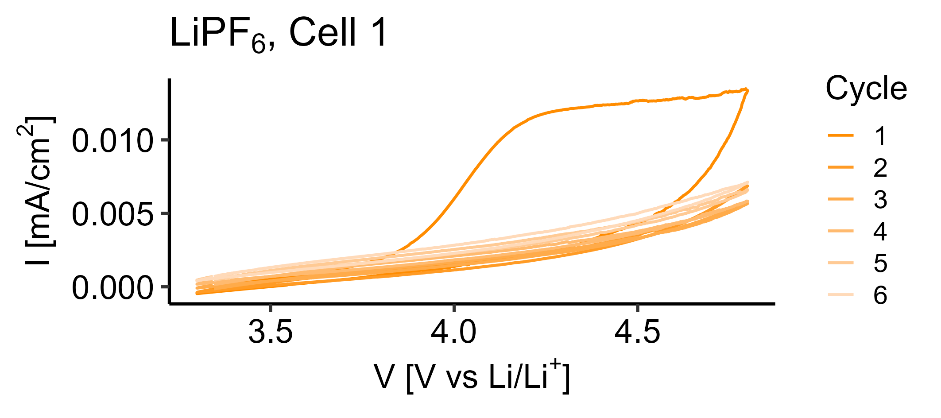


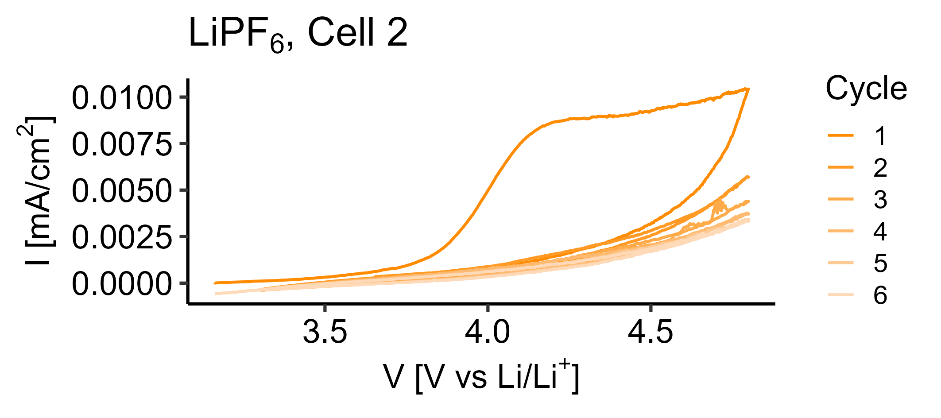


**Figure S3.2.3** Cyclic voltammetry of 1 M LiPF_6_ in EC:EMC (3:7 v/v) (LP57) electrolyte measured in three-electrode cell at scan rate 5 mV s^−1^. Working electrode: aluminium, counter electrode: lithium, reference electrode: lithium.

**
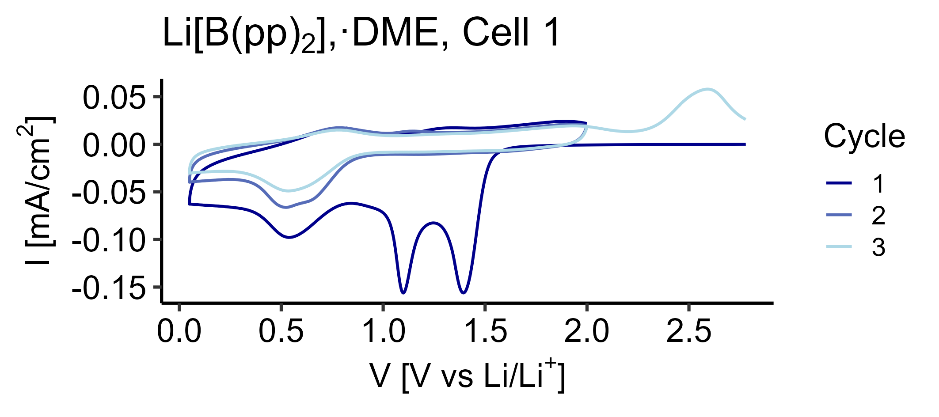
**

**
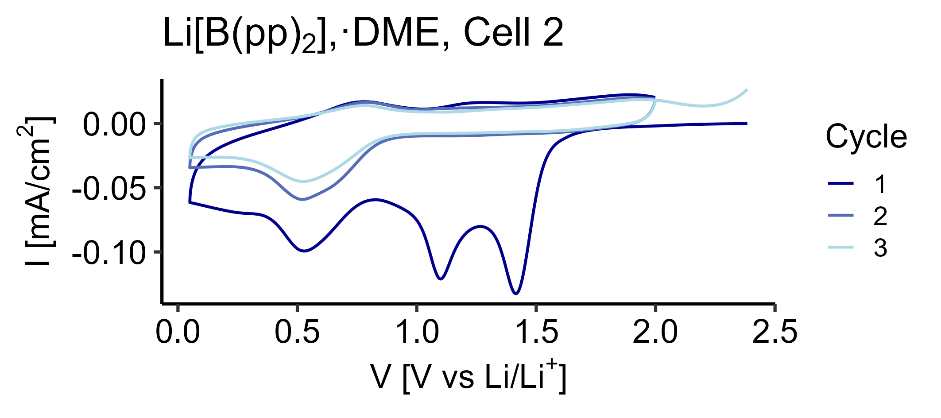
**

**
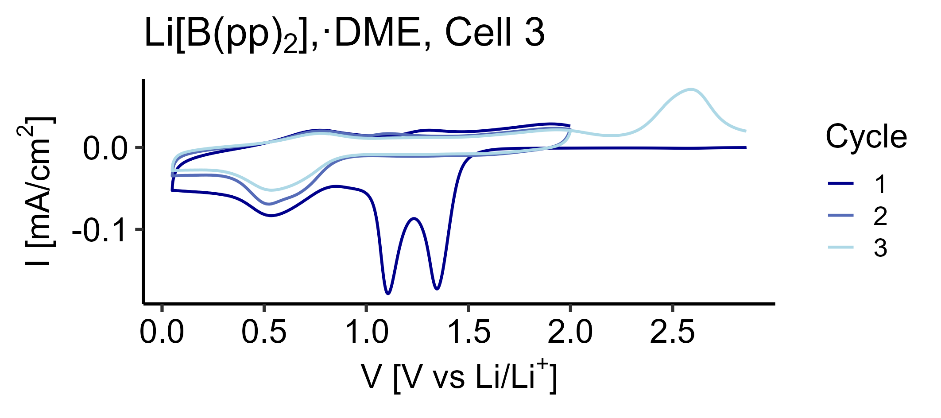
**

**Figure S3.2.4** Cyclic voltammetry of 1 M Li[B(pp)_2_]⋅DME in EC:EMC (3:7 v/v) electrolyte measured in three-electrode cell at scan rate 5 mV s^−1^. Working electrode: copper, counter electrode: lithium, reference electrode: lithium.

**
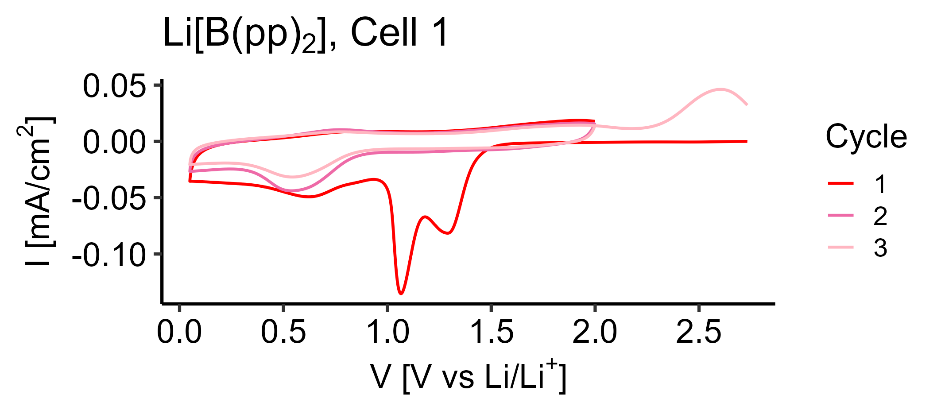
**

**
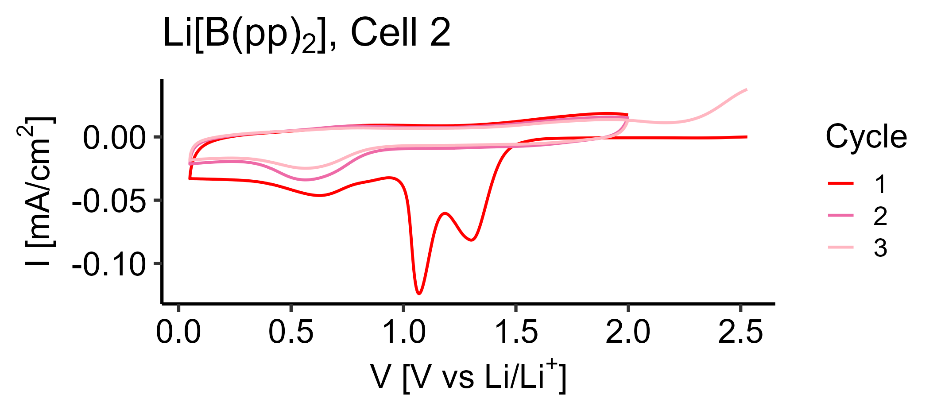
**

**Figure S3.2.5** Cyclic voltammetry of 0.1 M Li[B(pp)_2_] in EC:EMC (3:7 v/v) electrolyte measured in three-electrode cell at scan rate 5 mV s^−1^. Working electrode: copper, counter electrode: lithium, reference electrode: lithium.


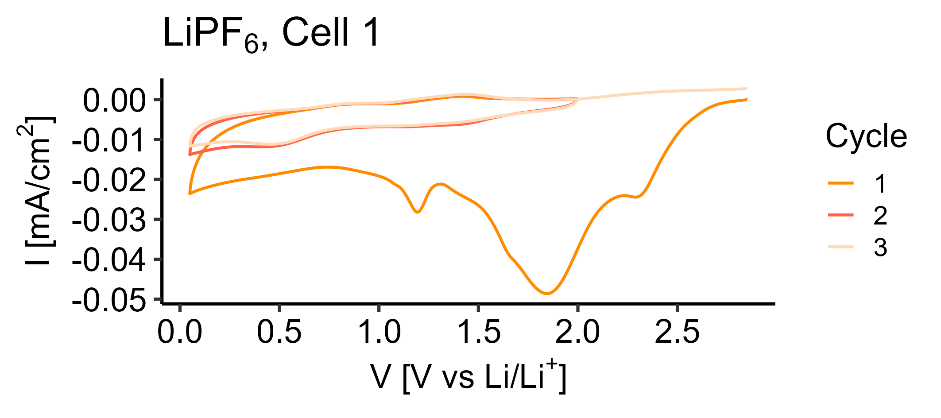


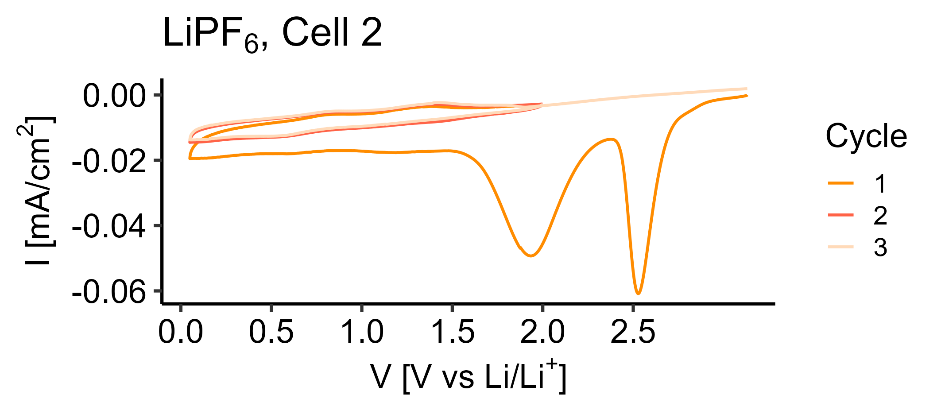


**Figure S3.2.6** Cyclic voltammetry of 1 M LiPF_6_ in EC:EMC (3:7 v/v) (LP57) electrolyte measured in three-electrode cell at scan rate 5 mV s^−1^. Working electrode: copper, counter electrode: lithium, reference electrode: lithium.


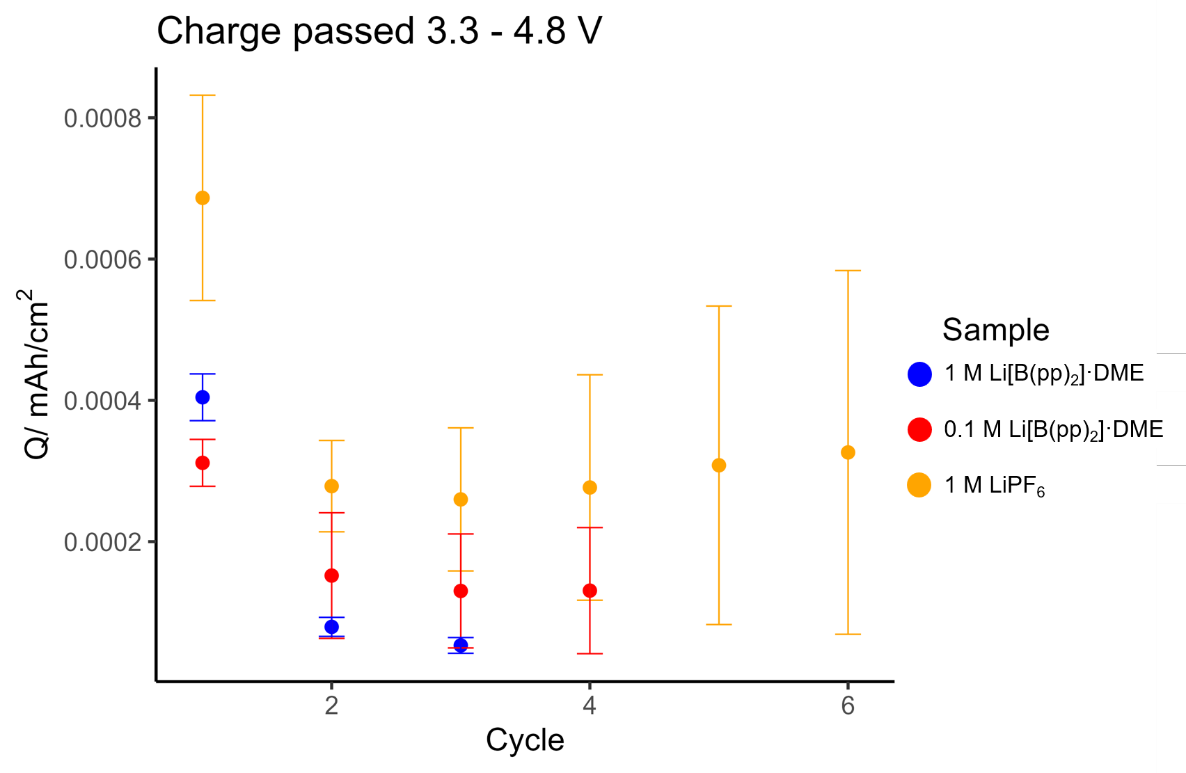


**Figure S3.2.7** Charge passed from 3.3 to 4.8 V, determined from integration of current (I) *vs*. time (t) plots using aluminium working electrode CV.

**S3.3 Electrochemical impedance spectroscopy measurements.**


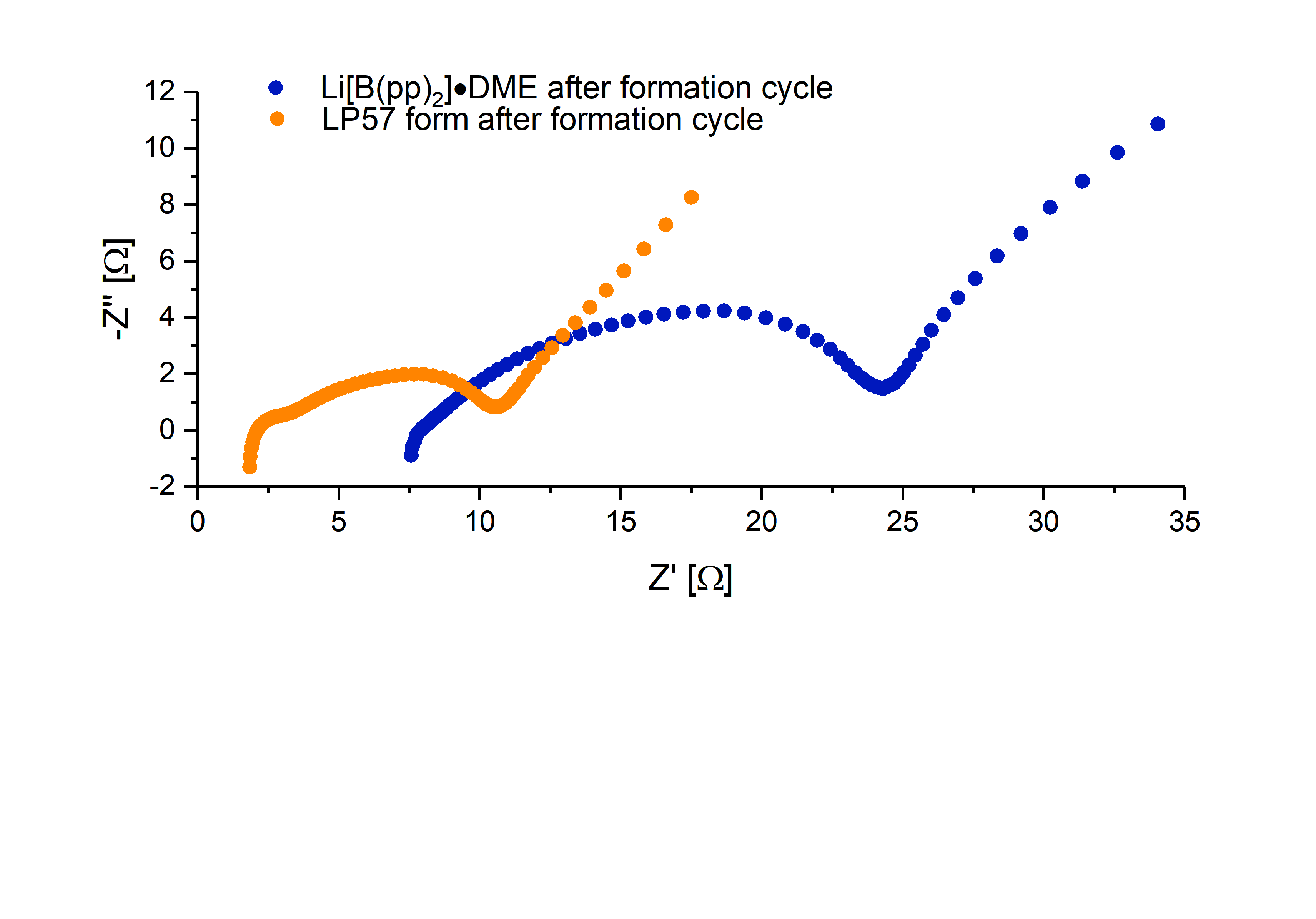


**Figure S3.3.1** EIS Nyquist plots after the formation cycle of coin cells using 1 M Li[B(PP)_2_]·DME (blue) and LP57 (LiPF_6_, orange) electrolytes. Measured using impedance spectroscopy with a frequency range of 1 MHz–0.01 Hz.


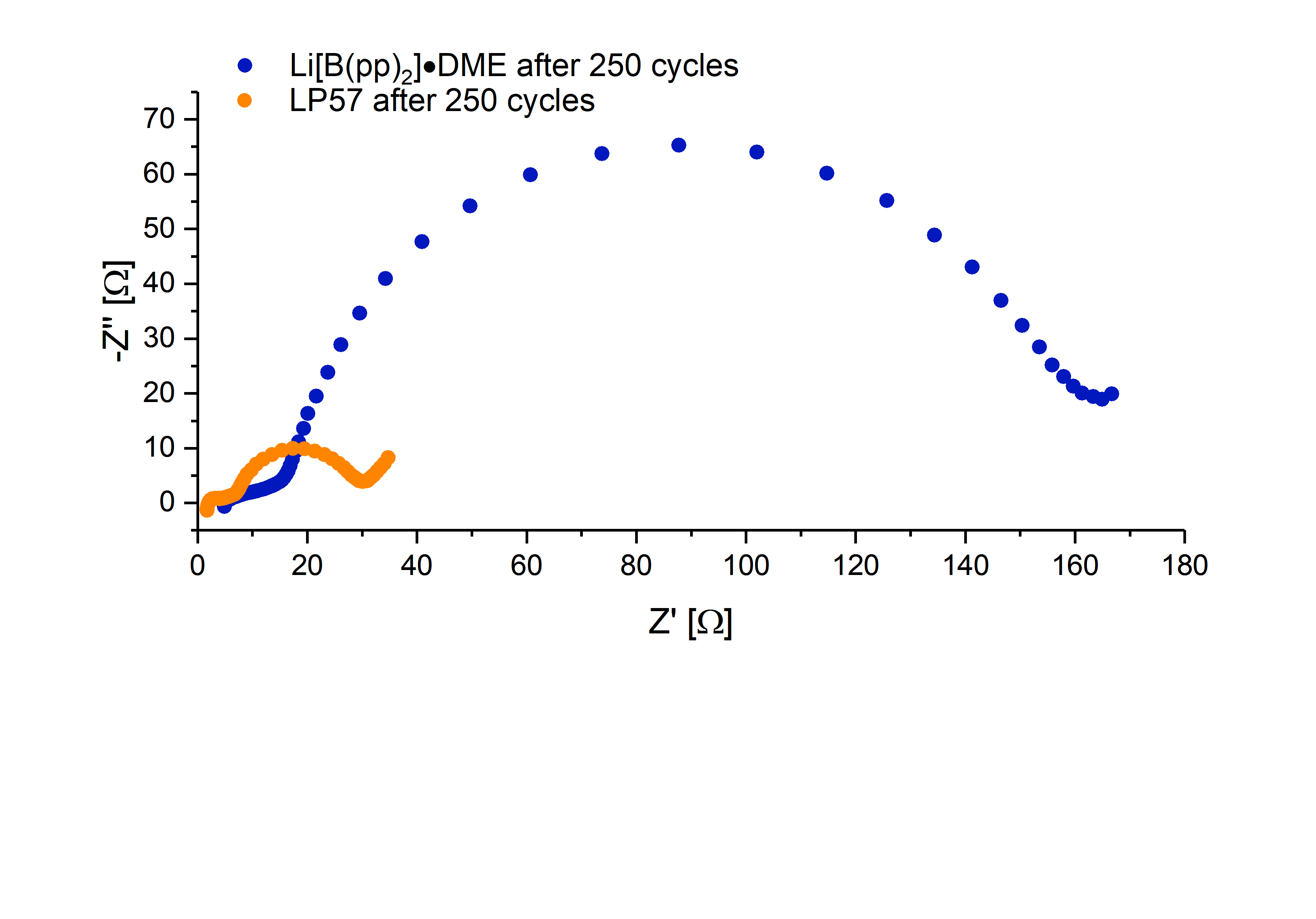


**Figure S3.3.2** EIS Nyquist plots after 250 cycles of coin cells using Li[B(PP)_2_]·DME (blue) and LP57 (LiPF_6_, orange) electrolytes. Measured using impedance spectroscopy with a frequency range of 1 MHz–0.01 Hz.


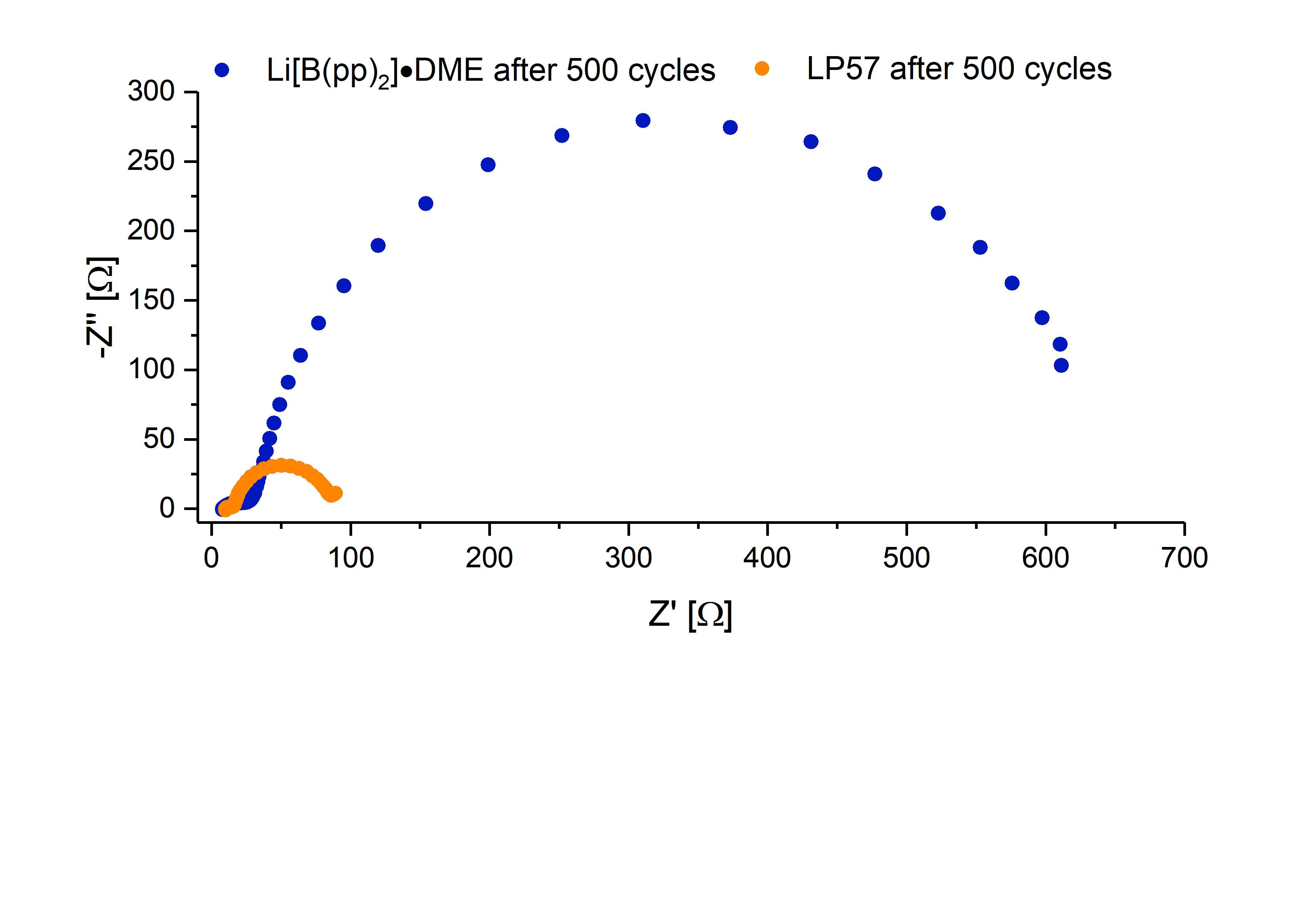


**Figure S3.3.3** EIS Nyquist plots after 500 cycles of coin cells using Li[B(PP)_2_]·DME (blue) and LP57 (LiPF_6_, orange) electrolytes. Measured using impedance spectroscopy with a frequency range of 1 MHz–0.01 Hz.


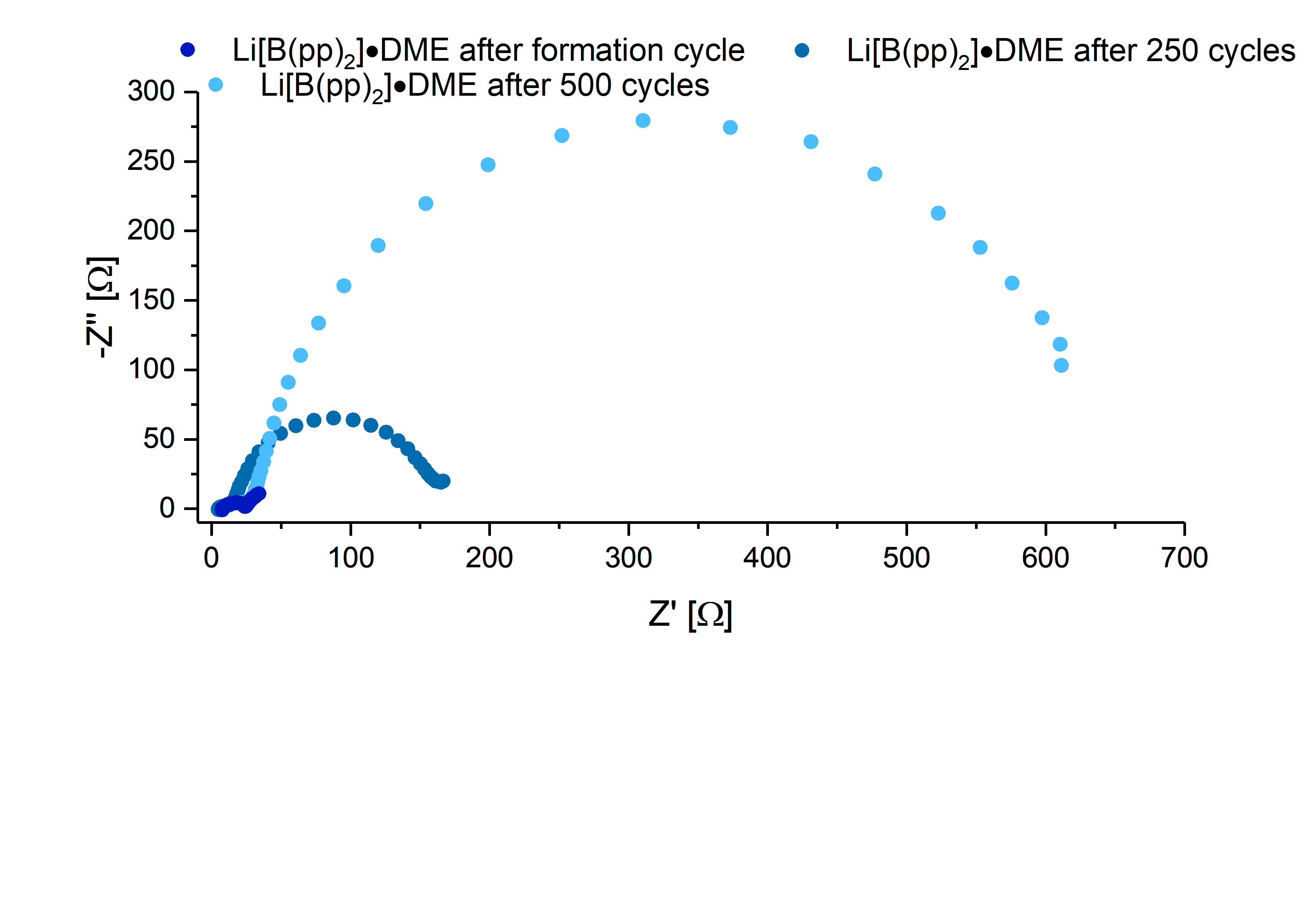


**Figure S3.3.4** EIS Nyquist plots overlay after formation cycle, 250 cycles and 500 cycles in coin cells using Li[B(PP)_2_]·DME electrolyte. Measured using impedance spectroscopy with a frequency range of 1 MHz–0.01 Hz.


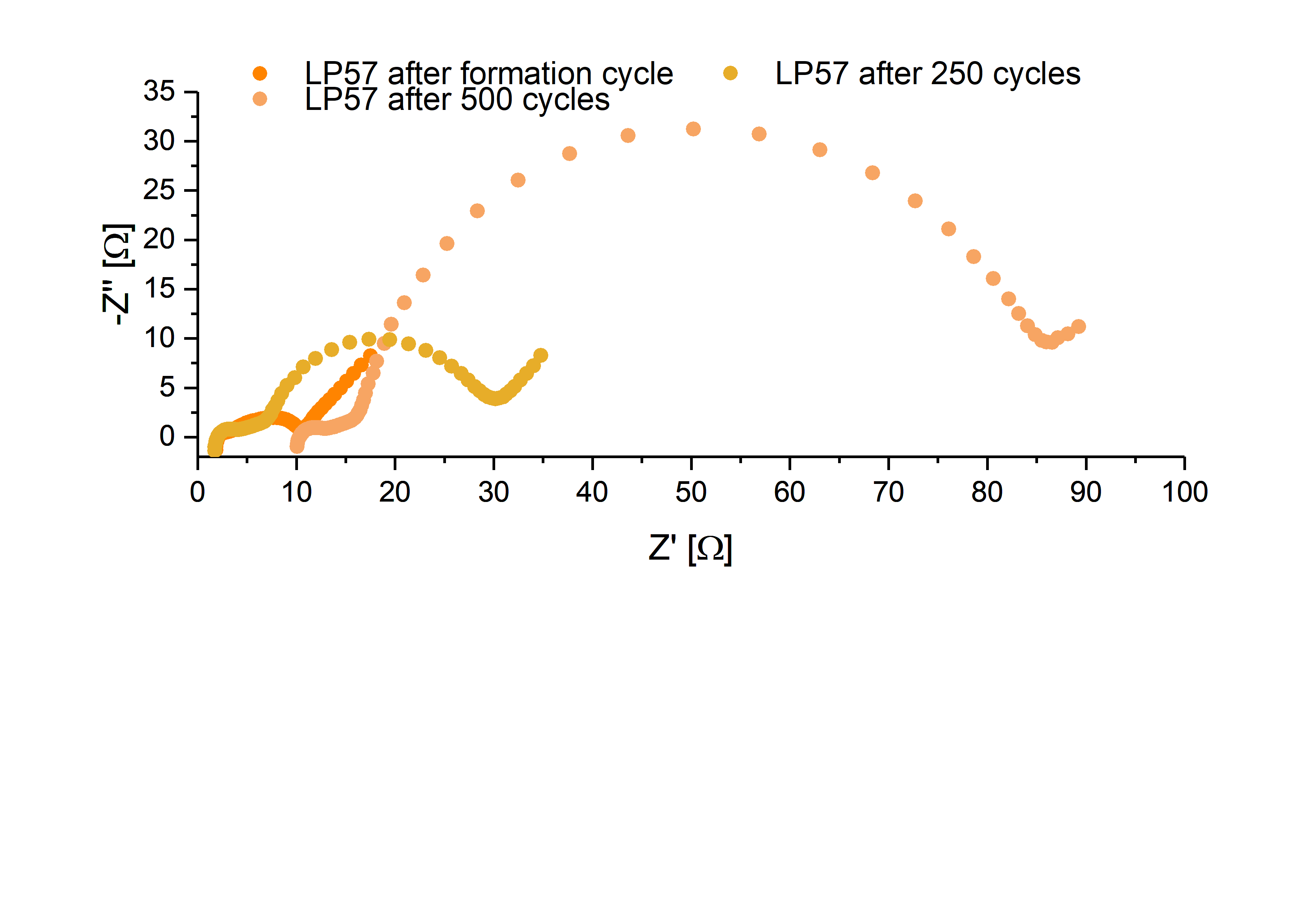


**Figure S3.3.5** EIS Nyquist plots overlay after formation cycle, 250 cycles and 500 cycles in coin cells using LP57 electrolyte. Measured using impedance spectroscopy with a frequency range of 1 MHz–0.01 Hz.

**S3.4 Lithium-ion cycling.**

**
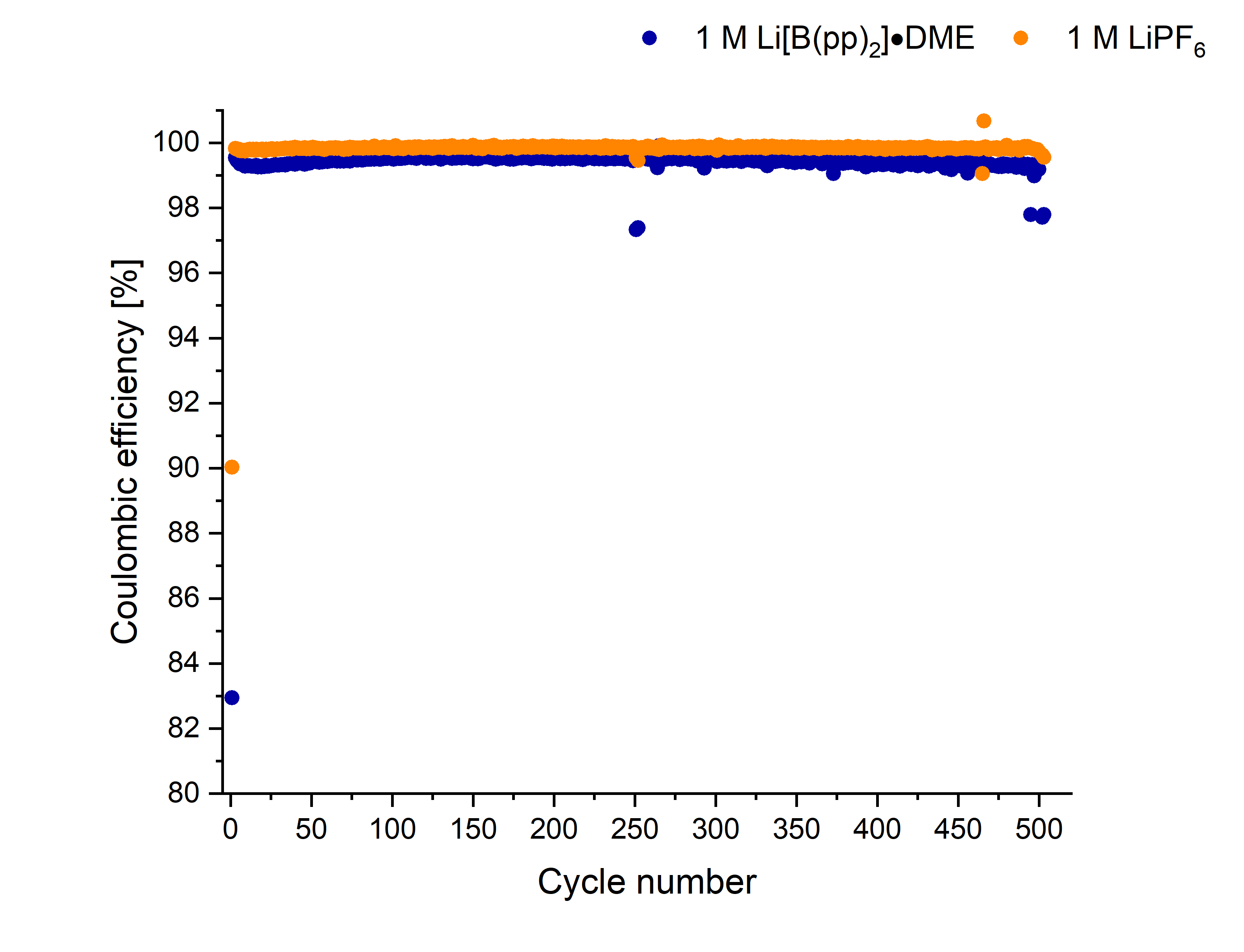
**

**Figure S3.4.1** Coulombic efficiency vs. cycle number for 1 M Li[B(pp)_2_]⋅DME (**1a**) EC:EMC (blue) and 1 M LiPF_6_ EC:EMC (orange, LP57). Active electrode materials are NMC811 and graphite for cathode and anode respectively. Approximate constant current rate of 1C for charge and discharge using cell voltage limits of 4.2 and 2.5 V. Measured in coin cells.


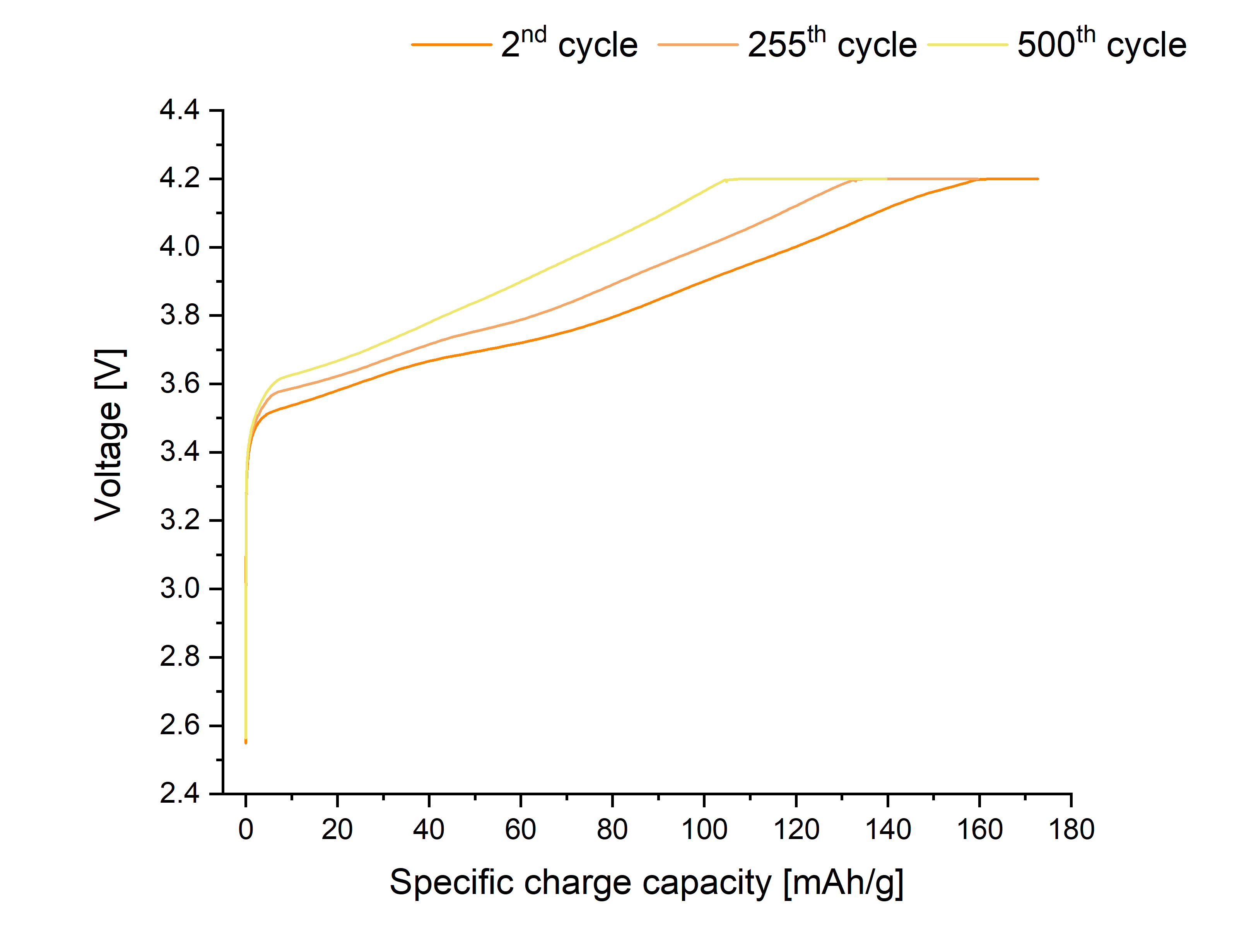


**Figure S3.4.2** Voltage vs. specific charge capacity for the 2^nd^, 255^th^ and 500^th^ cycle for 1 M LiPF_6_ EC:EMC (orange, LP57). Active electrode materials are NMC811 and graphite for cathode and anode respectively. Approximate constant current rate of 1C for charge and discharge using cell voltage limits of 4.2 and 2.5 V. Measured in coin cells.


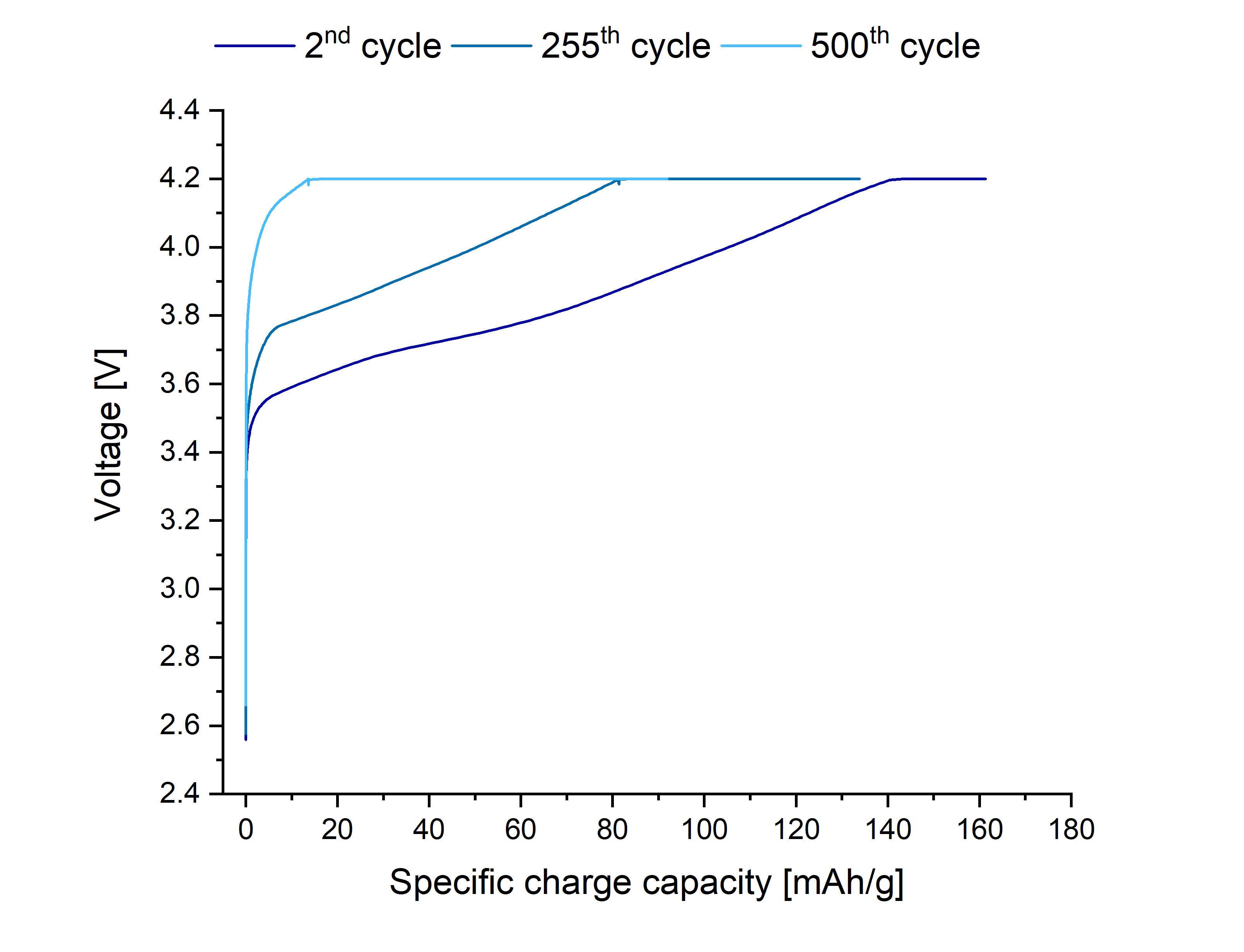


**Figure S3.4.3** Voltage vs. specific charge capacity for the 2^nd^, 255^th^ and 500^th^ cycle for 1 M Li[B(pp)_2_]⋅DME (**1a**) EC:EMC (blue). Active electrode materials are NMC811 and graphite for cathode and anode respectively. Approximate constant current rate of 1C for charge and discharge using cell voltage limits of 4.2 and 2.5 V. Measured in coin cells.

**
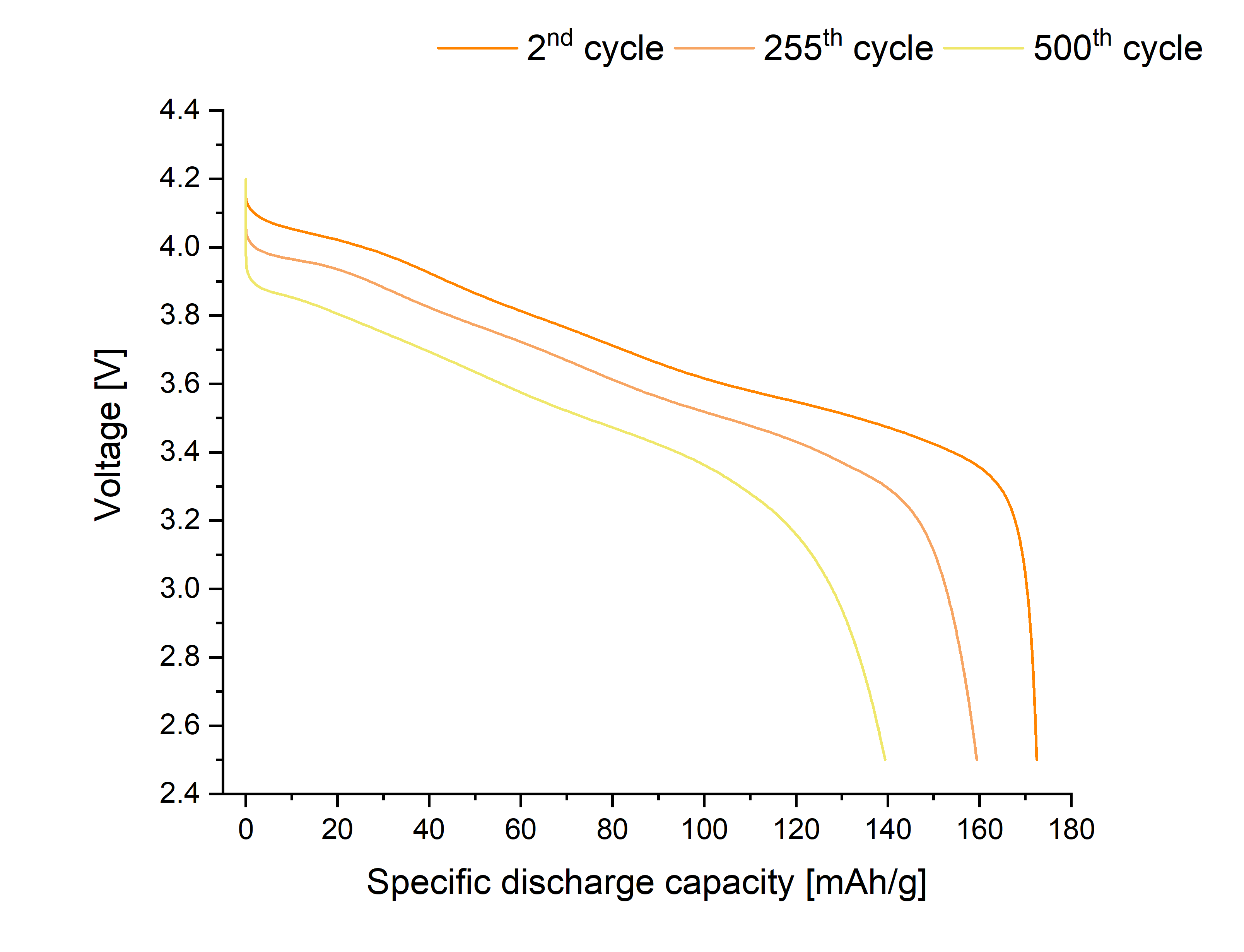
**

**Figure S3.4.4** Voltage vs. specific discharge capacity for the 2^nd^, 255^th^ and 500^th^ cycle for 1 M LiPF_6_ EC:EMC (orange, LP57). Active electrode materials are NMC811 and graphite for cathode and anode respectively. Approximate constant current rate of 1C for charge and discharge using cell voltage limits of 4.2 and 2.5 V. Measured in coin cells.

**
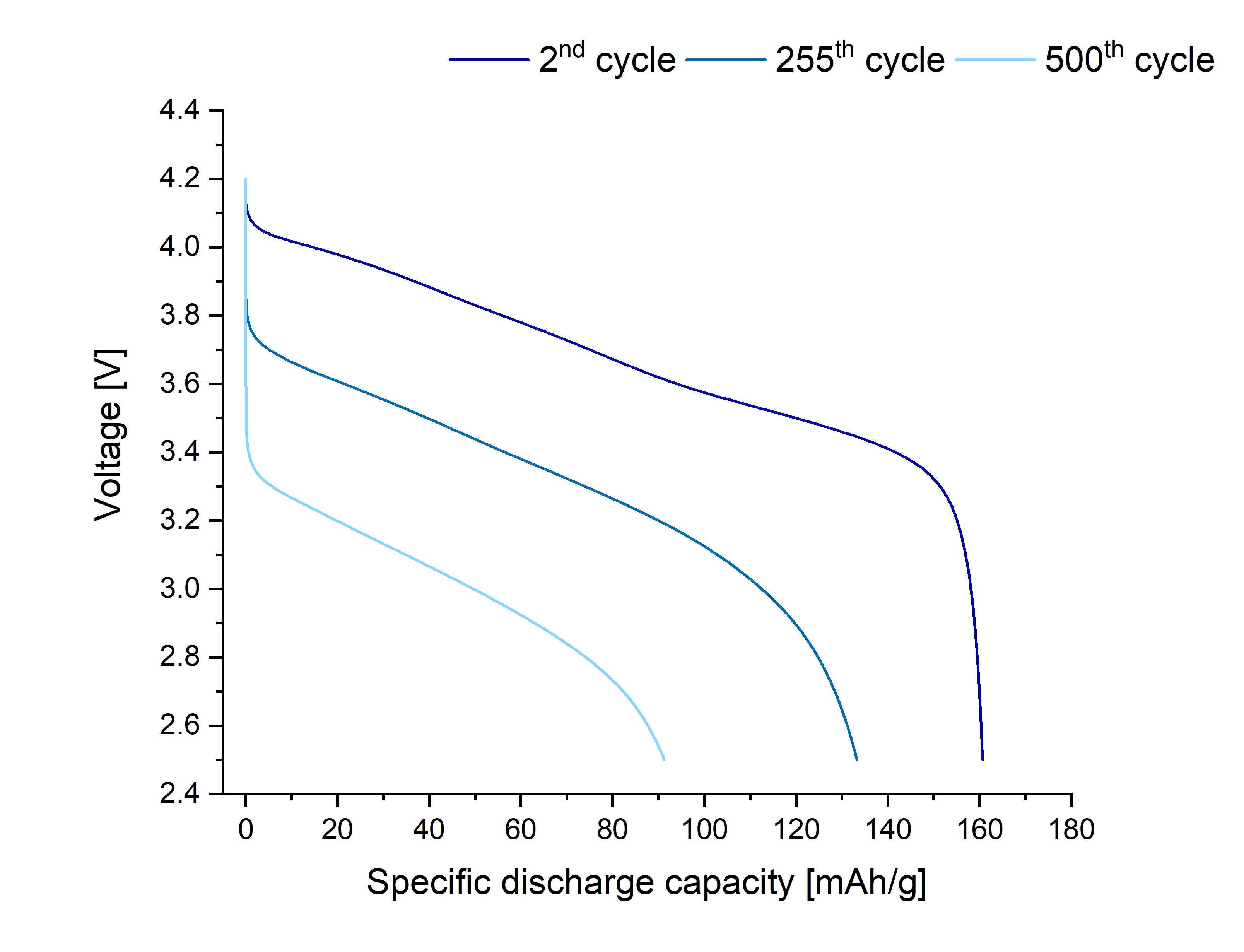
**

**Figure S3.4.5** Voltage vs. specific discharge capacity for the 2^nd^, 255^th^ and 500^th^ cycle for 1 M Li[B(pp)_2_]⋅DME (**1a**) EC:EMC (blue). Active electrode materials are NMC811 and graphite for cathode and anode respectively. Approximate constant current rate of 1C for charge and discharge using cell voltage limits of 4.2 and 2.5 V. Measured in coin cells.


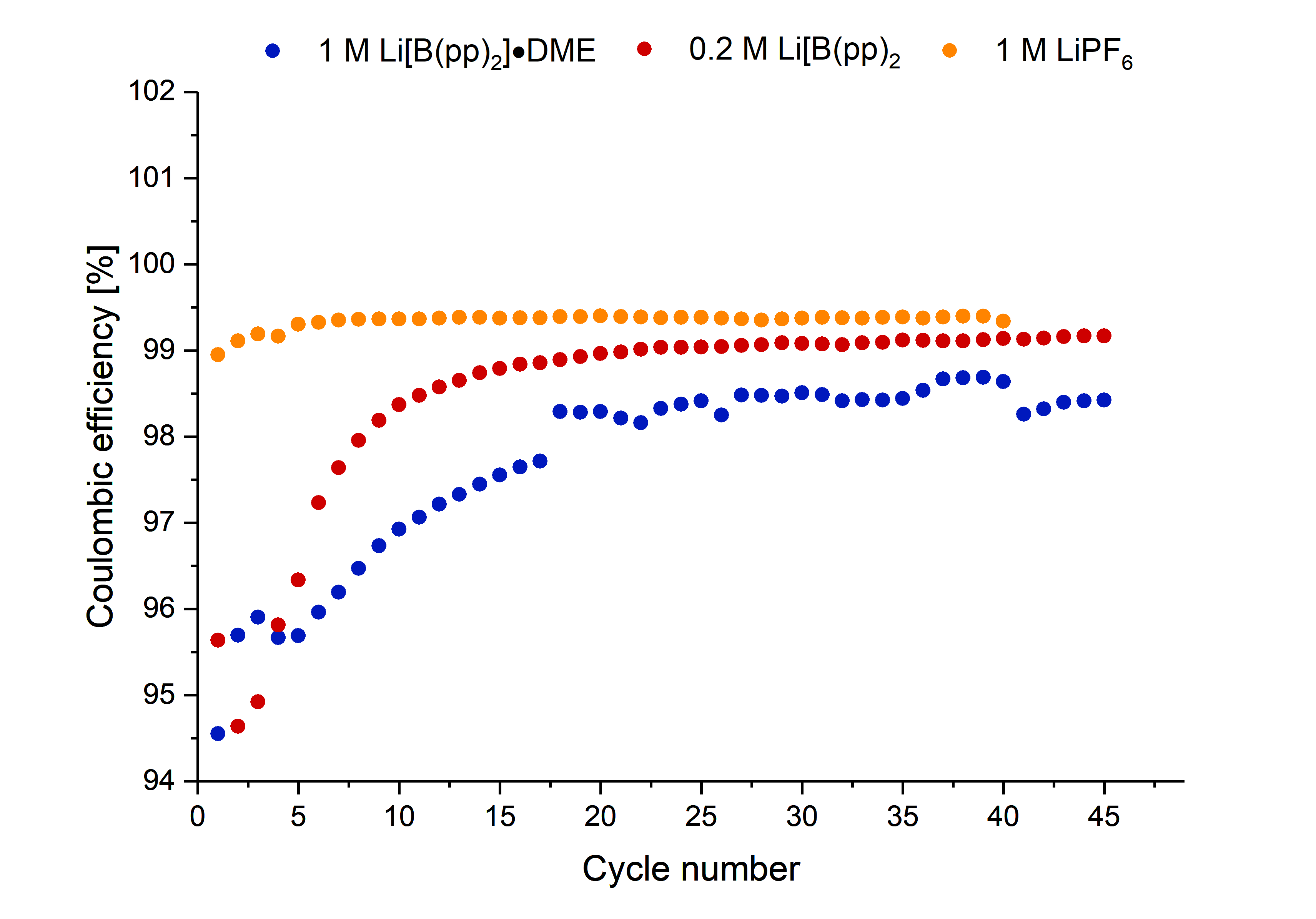


**Figure S3.4.6** Coulombic efficiency vs. cycle number collected from the first 45 cycles for 1 M Li[B(pp)_2_]⋅DME (**1a**) EC:EMC (blue) and 0.2 M Li[B(pp)_2_] (**1b**) EC:EMC (red) and 40 cycles for 1 M LiPF_6_ EC:EMC (orange, LP57). Active electrode materials are NMC811 and graphite for cathode and anode respectively. Approximate constant current rate of C/3 for charge and discharge using cell voltage limits of 4.2 and 2.5 V. Measured in coin cells.

**
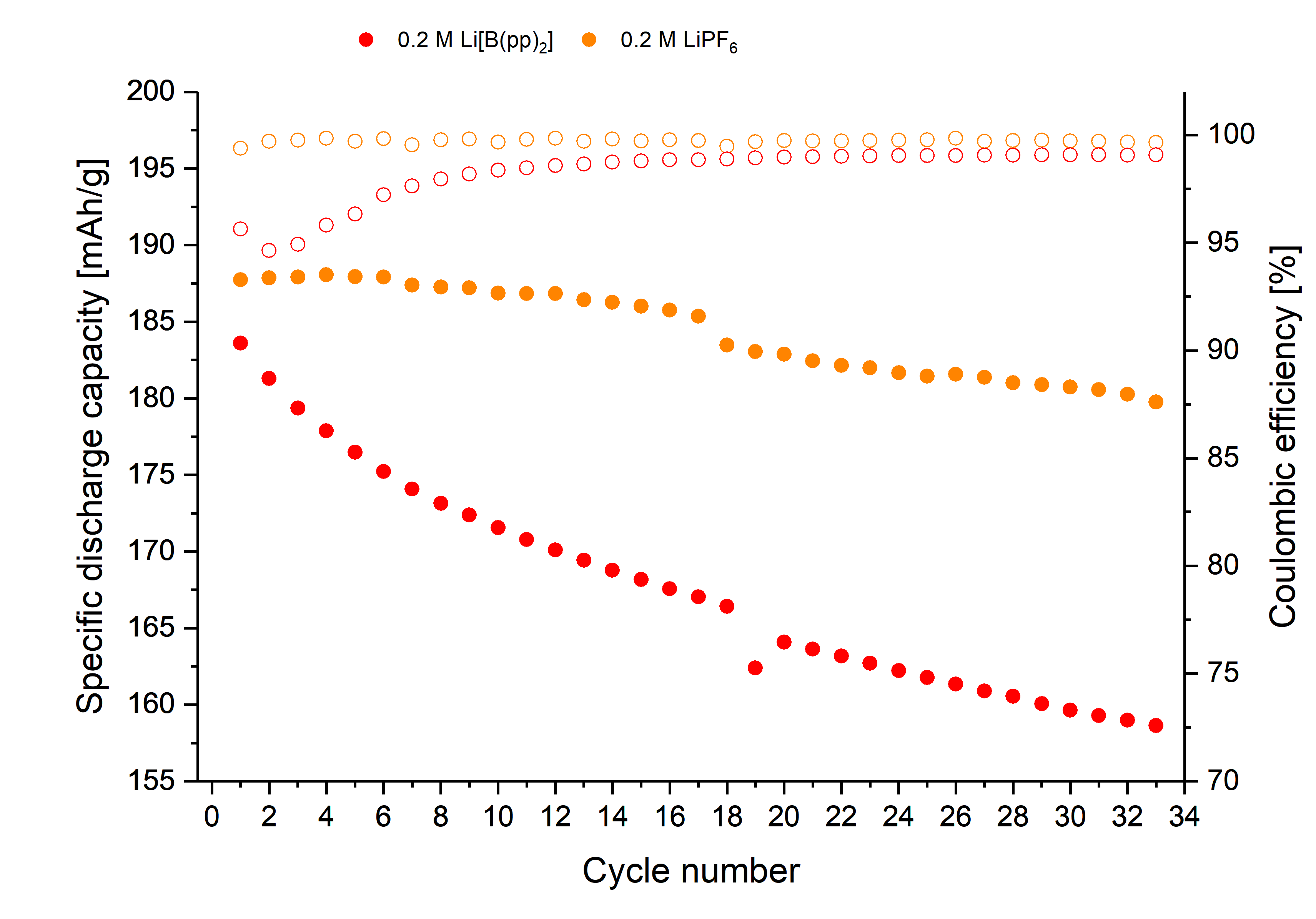
**

**Figure S3.4.7** Discharge gravimetric capacity (filled circles) and Coulombic efficiency (non-filled circles) vs. cycle number collected from the first 33 cycles for 0.2 M Li[B(pp)_2_] (**1b**) EC:EMC and 0.2 M LiPF_6_ EC:EMC. Approximate constant current rate of C/3 for charge and discharge using cell voltage limits of 4.2 and 2.5 V. Measured in coin cells.

# **S4 X-ray photoelectron spectroscopy (XPS) measurements**


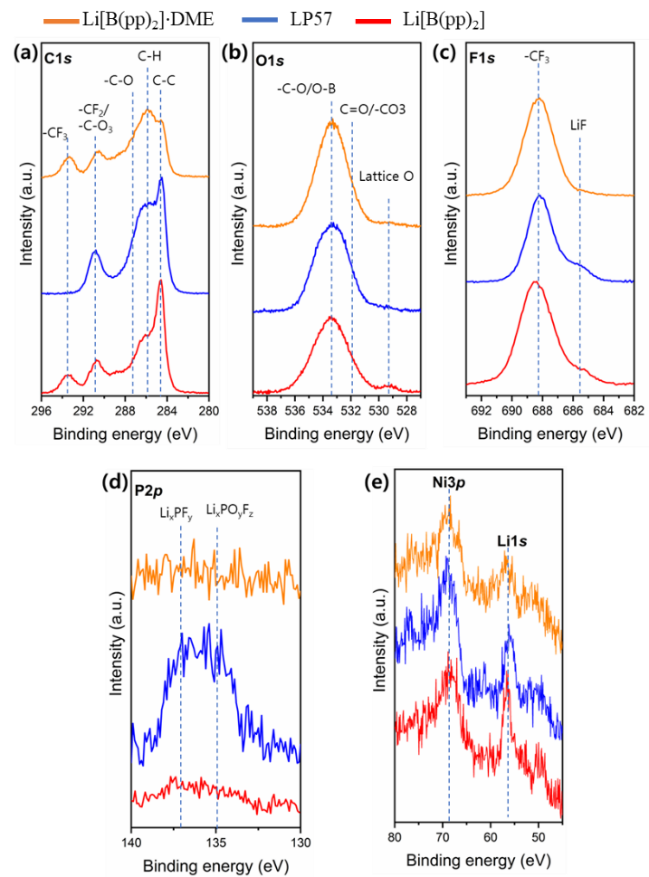


**Figure S4.1** (a) C1s, (b) O1s, (c) F1s, (d) P2p, and (e) Ni3p/Li1s XPS spectra of the NMC-811 cathode using 1 M Li[B(pp)_2_]⋅DME (**1a**) in EC:EMC (3:7 v/v) and LP57 electrolytes after 500 cycles and Li[B(pp)_2_] (**1b**) in EC:EMC (3:7 v/v) after 45 cycles.


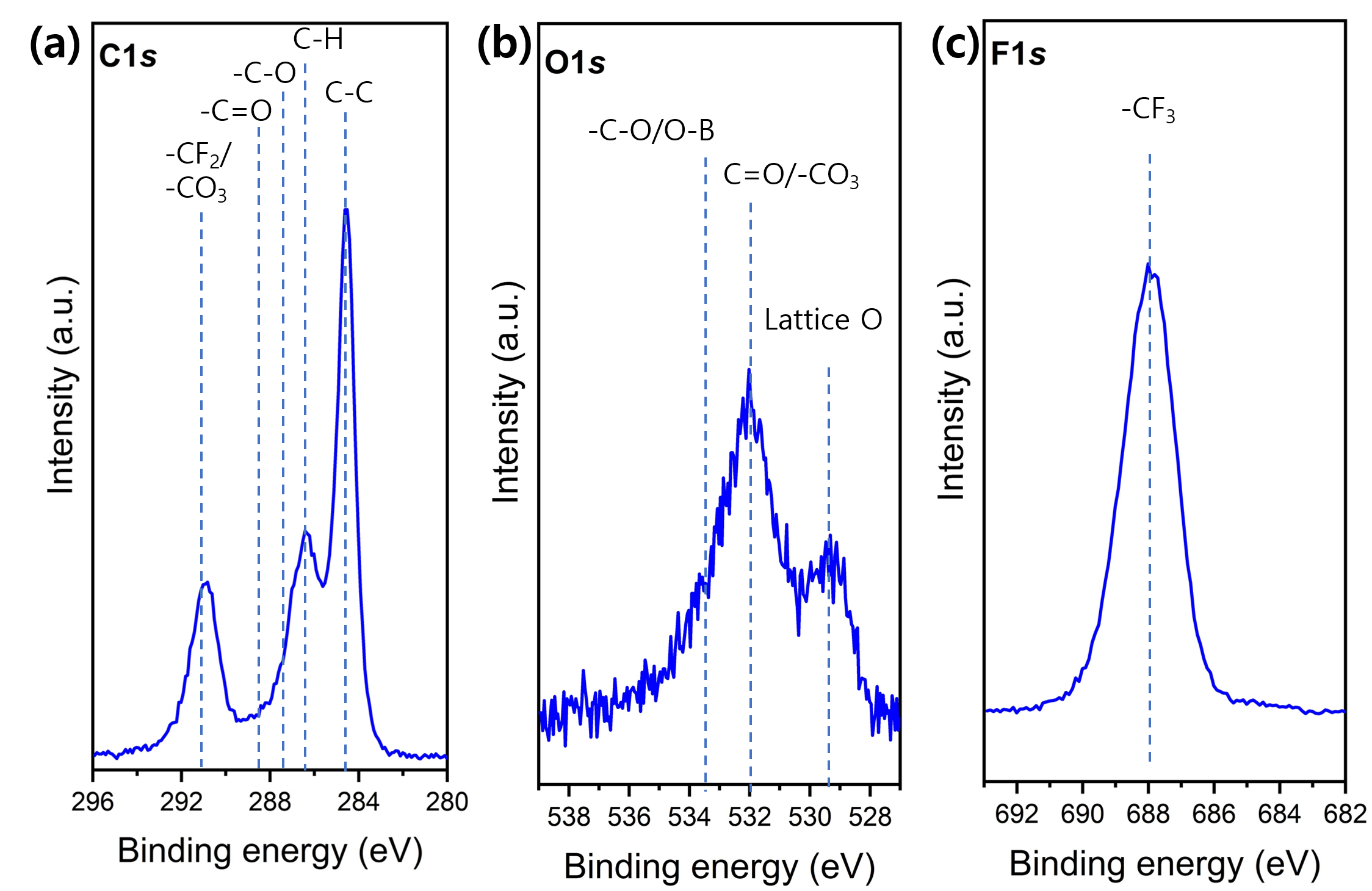


–CF_2_

**Figure S4.2** (a) (a) C1s, (b) O1s and (c) F1s XPS spectra of pristine NMC-811 electrode.

# **S5 NMR spectra.**

**S5.1 NMR spectra of lithium borate complexes**

**Figure S5.1.1** ^1^H NMR (400 MHz, CD_3_CN, 295 K) spectrum of lithium bis(perfluorinated pinacolato)borate, Li[B(pp)_2_]·DME.

**
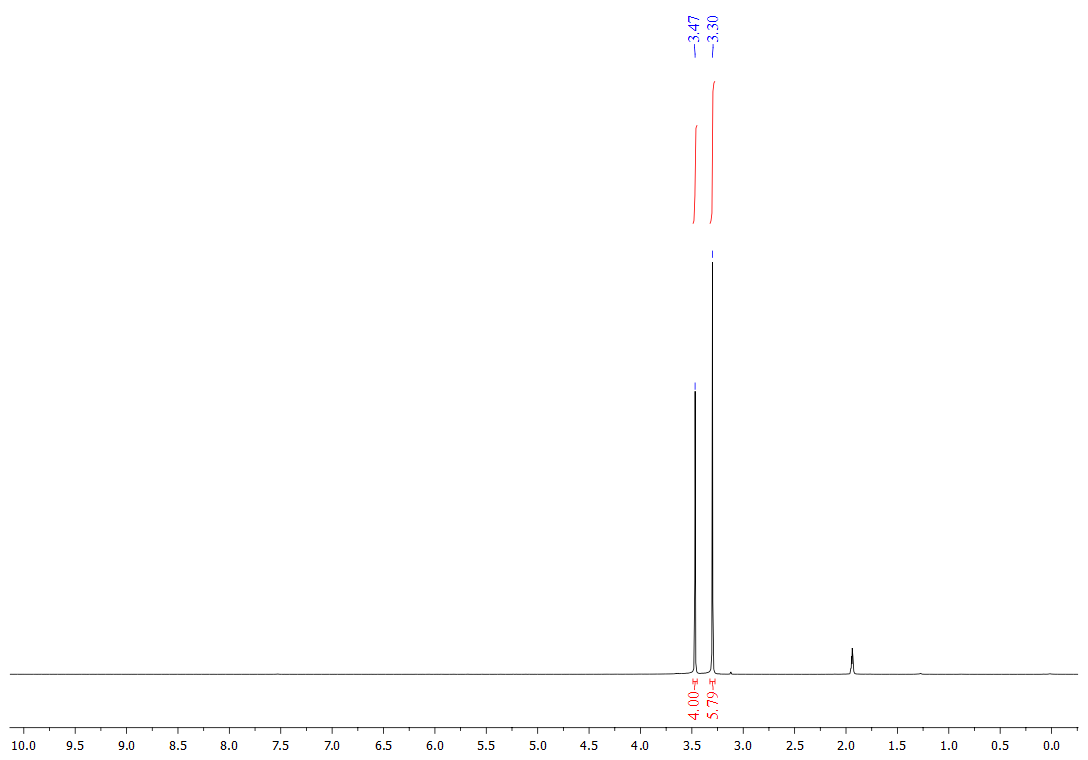
**

**Figure S5.1.2** ^13^C{^1^H} NMR (101 MHz, (CD_3_)_2_SO, 295 K) spectrum of lithium bis(perfluorinated pinacolato)borate, Li[B(pp)_2_]·DME.

**
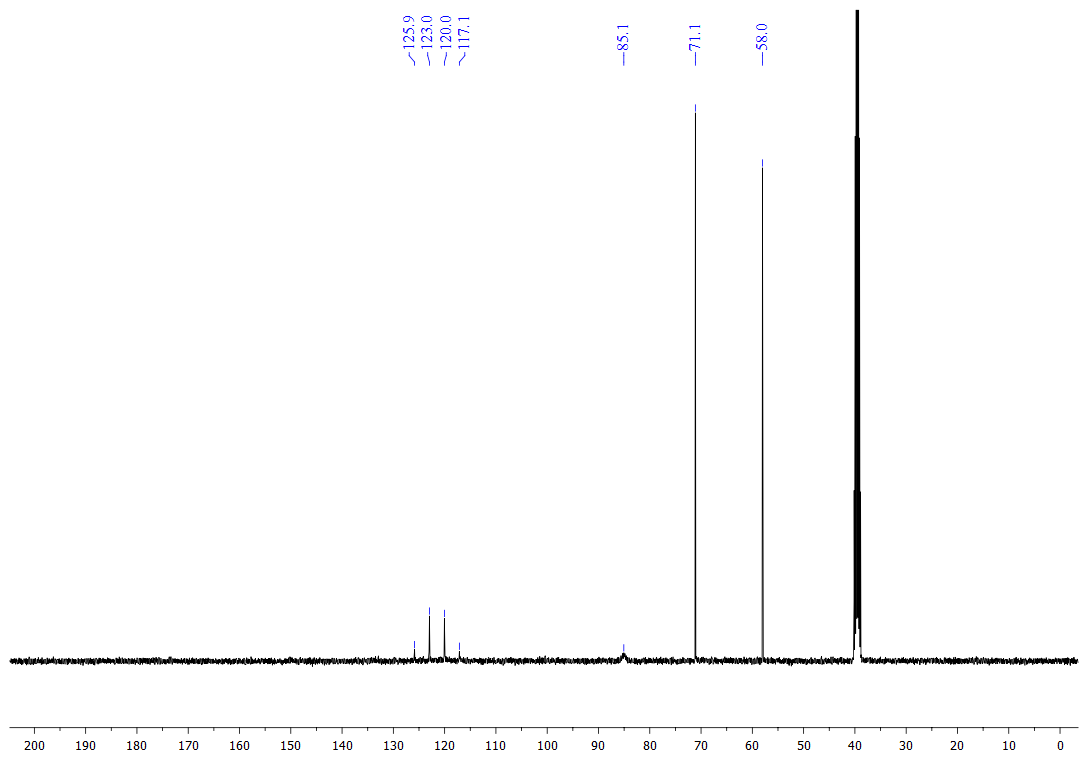
**

**Figure S5.1.3** ^11^B NMR (128 MHz, CD_3_CN, 295 K) spectrum of lithium bis(perfluorinated pinacolato)borate, Li[B(pp)_2_]·DME.

**
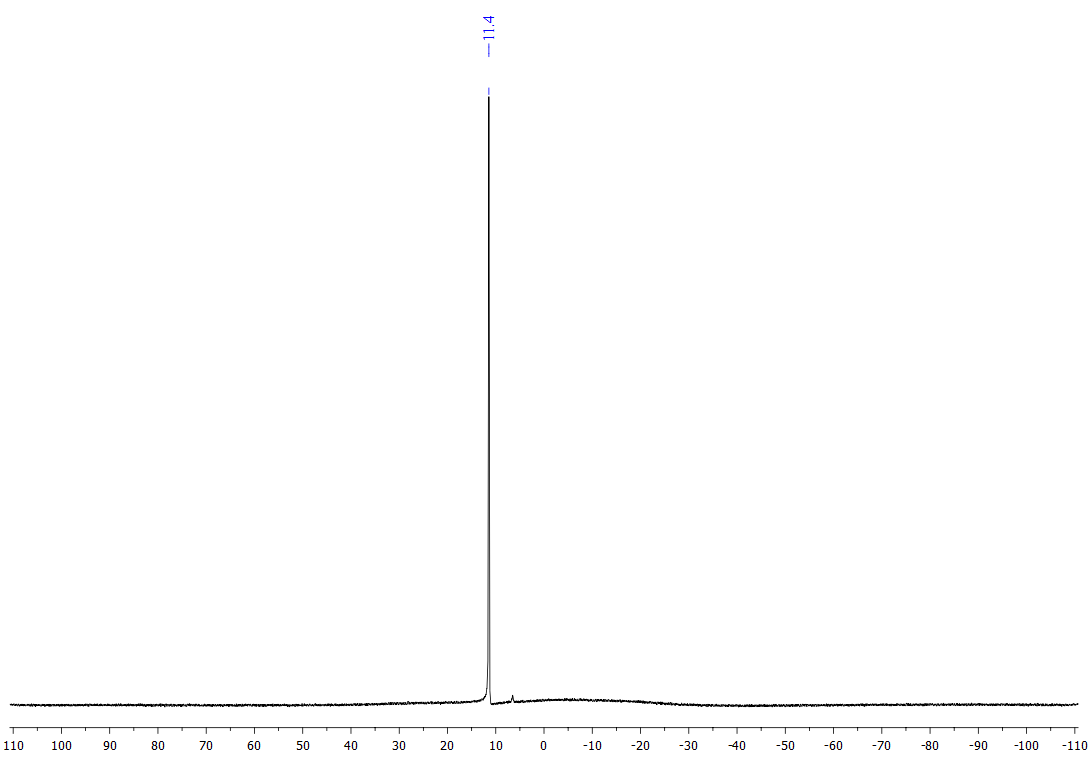
**

**Figure S5.1.4** ^19^F NMR (376 MHz, CD_3_CN, 295 K) spectrum of lithium bis(perfluorinated pinacolato)borate, Li[B(pp)_2_]·DME.

**
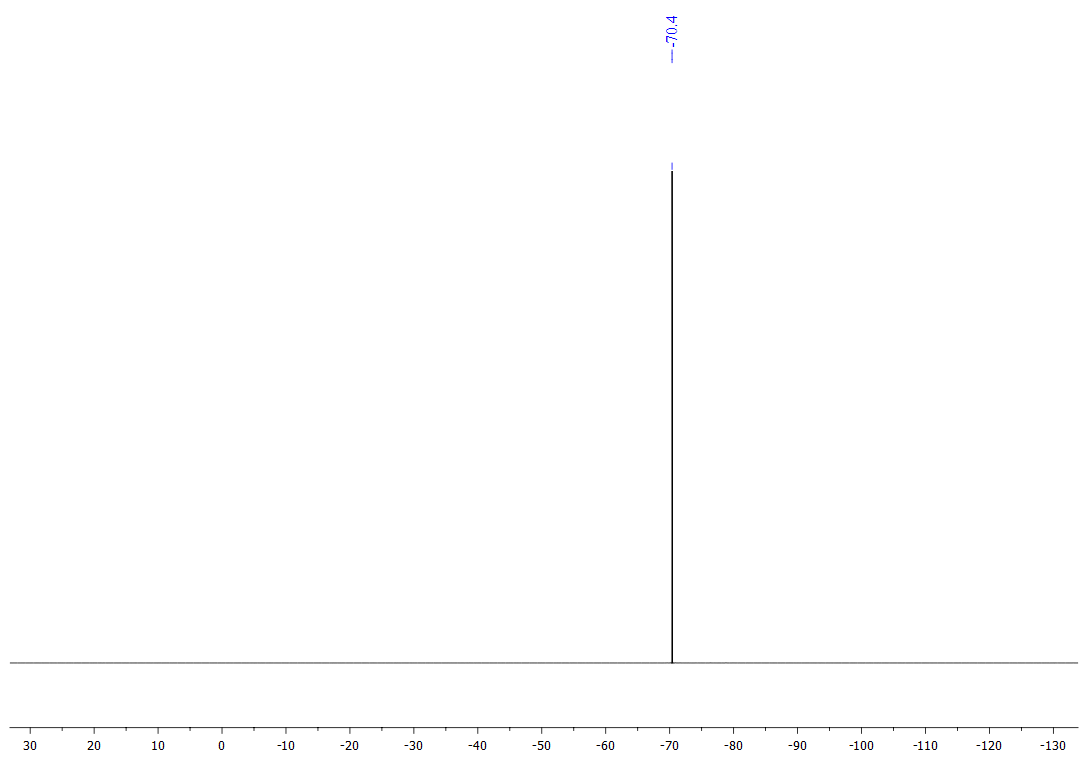
**

**Figure S5.1.5** ^7^Li NMR (155 MHz, CD_3_CN, 295 K) spectrum of lithium bis(perfluorinated pinacolato)borate, Li[B(pp)_2_]·DME.

**
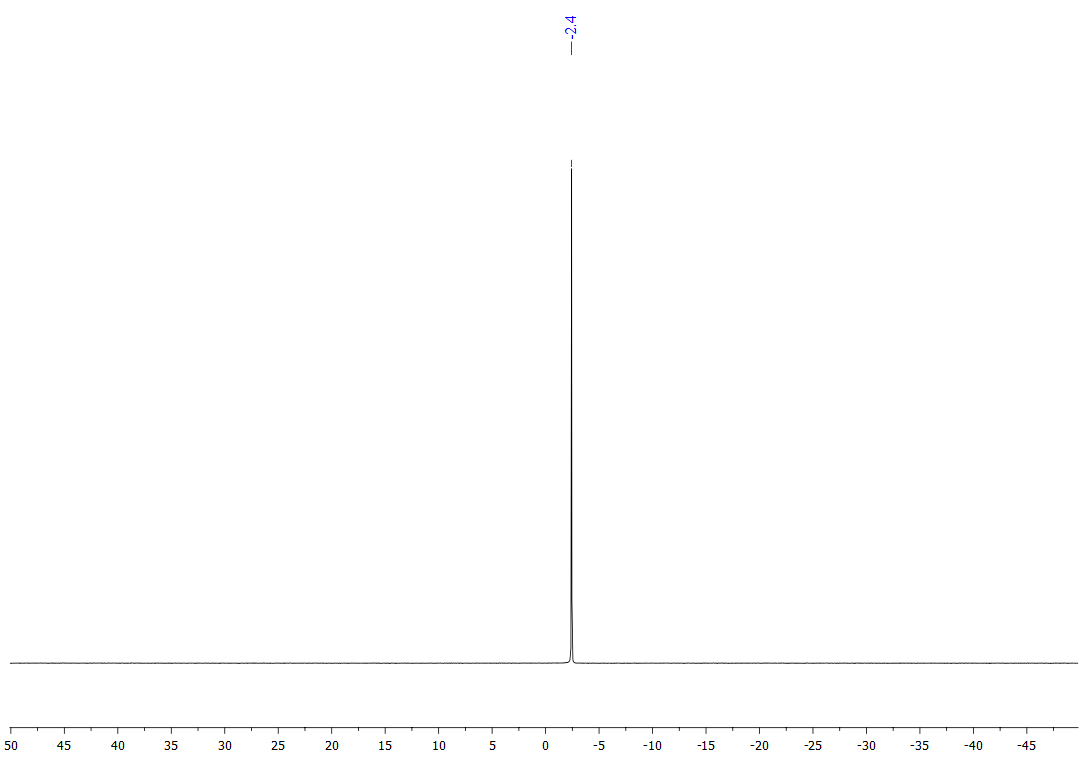
**

**Figure S5.1.6** ^1^H NMR (400 MHz, (CD_3_)_2_SO, 295 K) spectrum of 0.1 mmol of lithium bis(perfluorinated pinacolato)borate, Li[B(pp)_2_]·DME, and 0.1 mmol of 1,4-dimethoxybenzene as an internal standard.

**
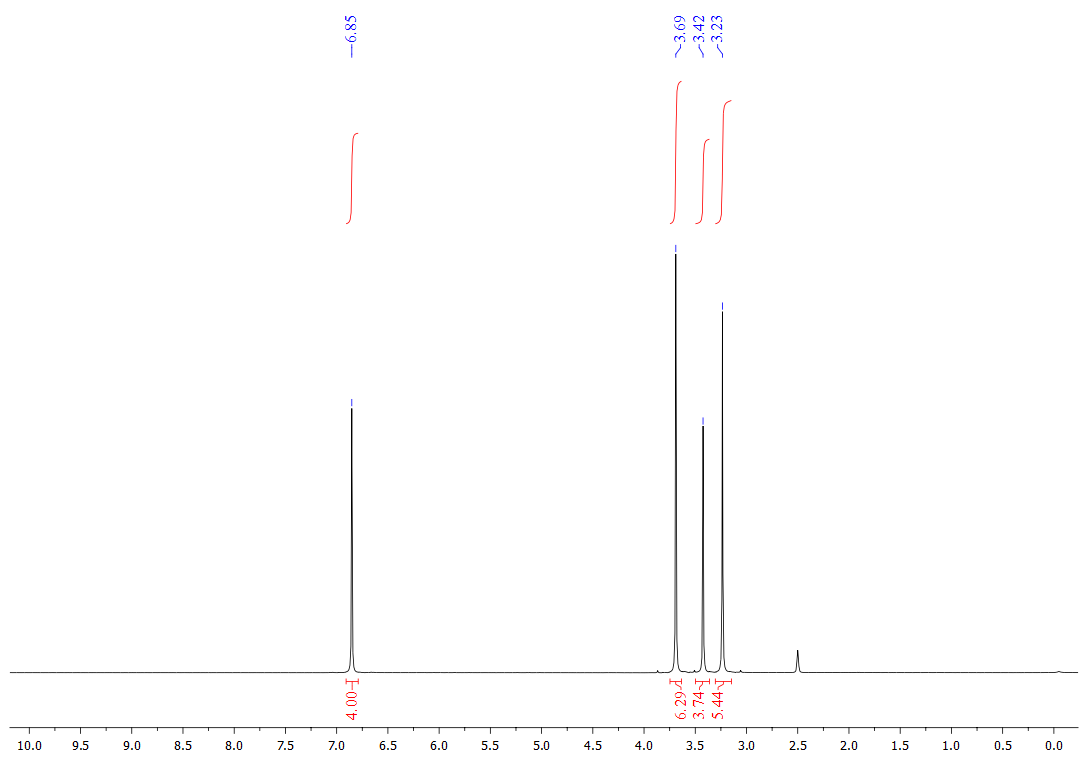
**

**Figure S5.1.7** ^1^H NMR (400 MHz, (CD_3_)_2_SO, 295 K) spectrum of lithium bis(perfluorinated pinacolato)borate, Li[B(pp)_2_].

**
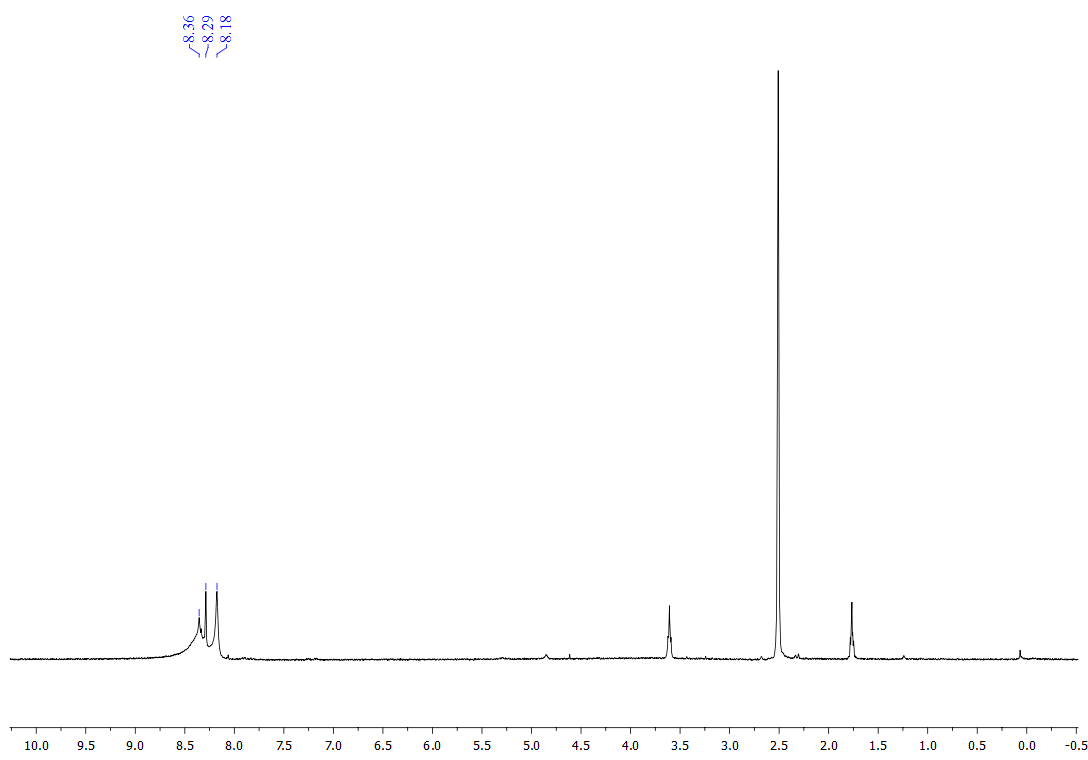
**

**Figure S5.1.8** ^13^C{^1^H} NMR (101 MHz, (CD_3_)_2_SO, 295 K) spectrum of lithium bis(perfluorinated pinacolato)borate, Li[B(pp)_2_].

**
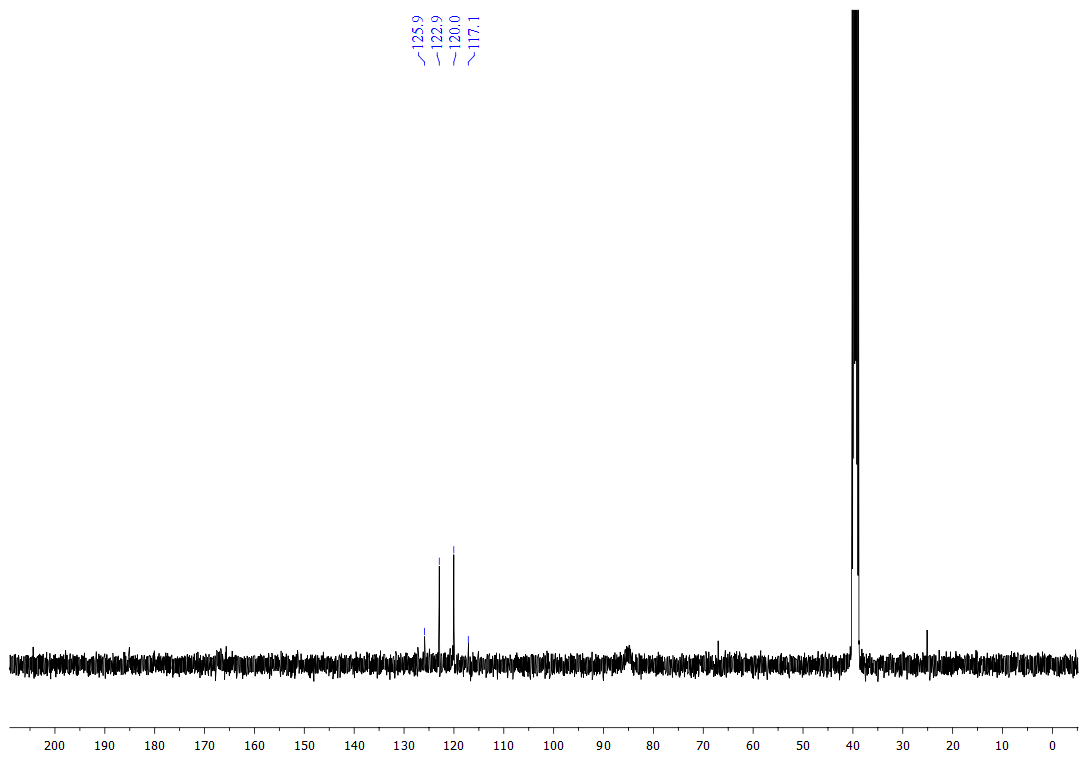
**

**Figure S5.1.9** ^11^B NMR (128 MHz, (CD_3_)_2_SO, 295 K) spectrum of unsolvated lithium bis(perfluorinated pinacolato)borate, Li[B(pp)_2_].

**
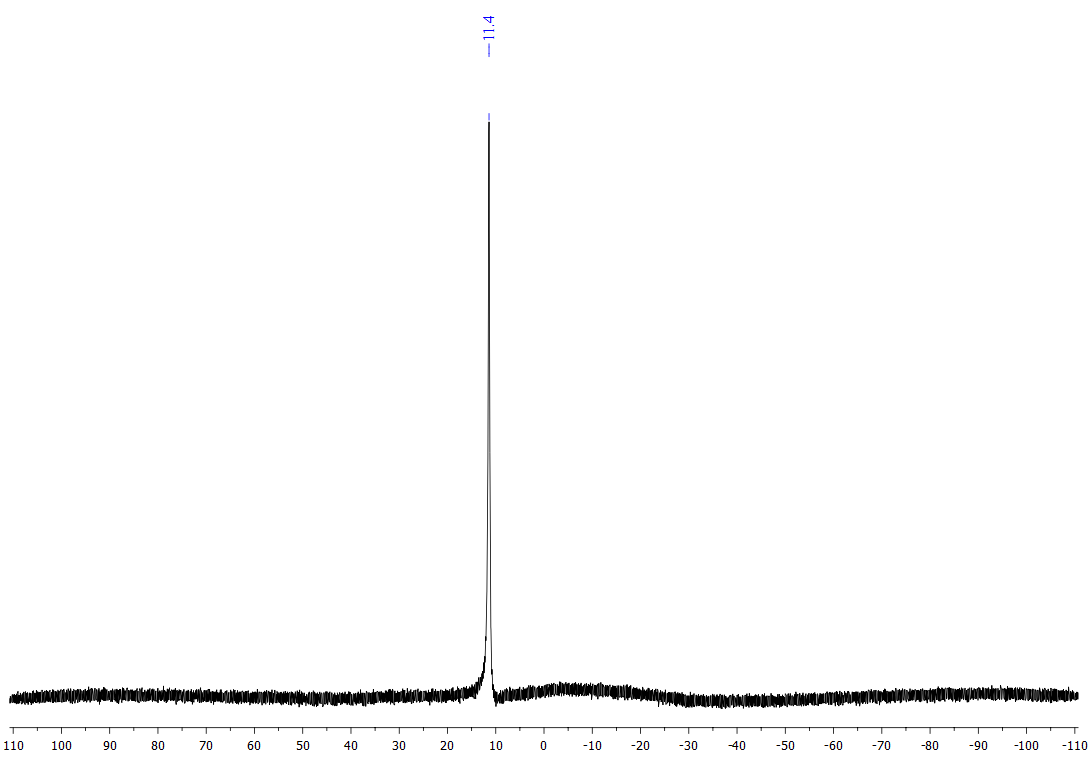
**
**Figure S5.1.10** ^19^F NMR (376 MHz, (CD_3_)_2_SO, 295 K) spectrum of unsolvated lithium bis(perfluorinated pinacolato)borate, Li[B(pp)_2_].


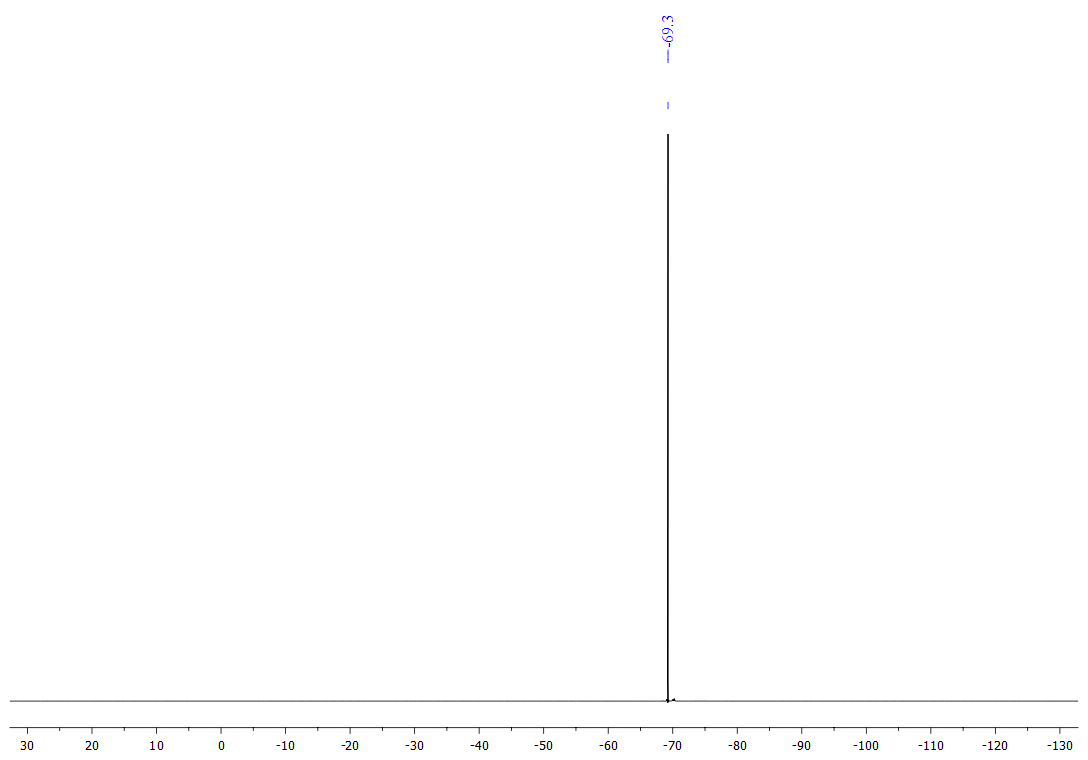


**Figure S5.1.11** ^7^Li NMR (155 MHz, (CD_3_)_2_SO, 295 K) spectrum of lithium bis(perfluorinated pinacolato)borate, Li[B(pp)_2_].

**
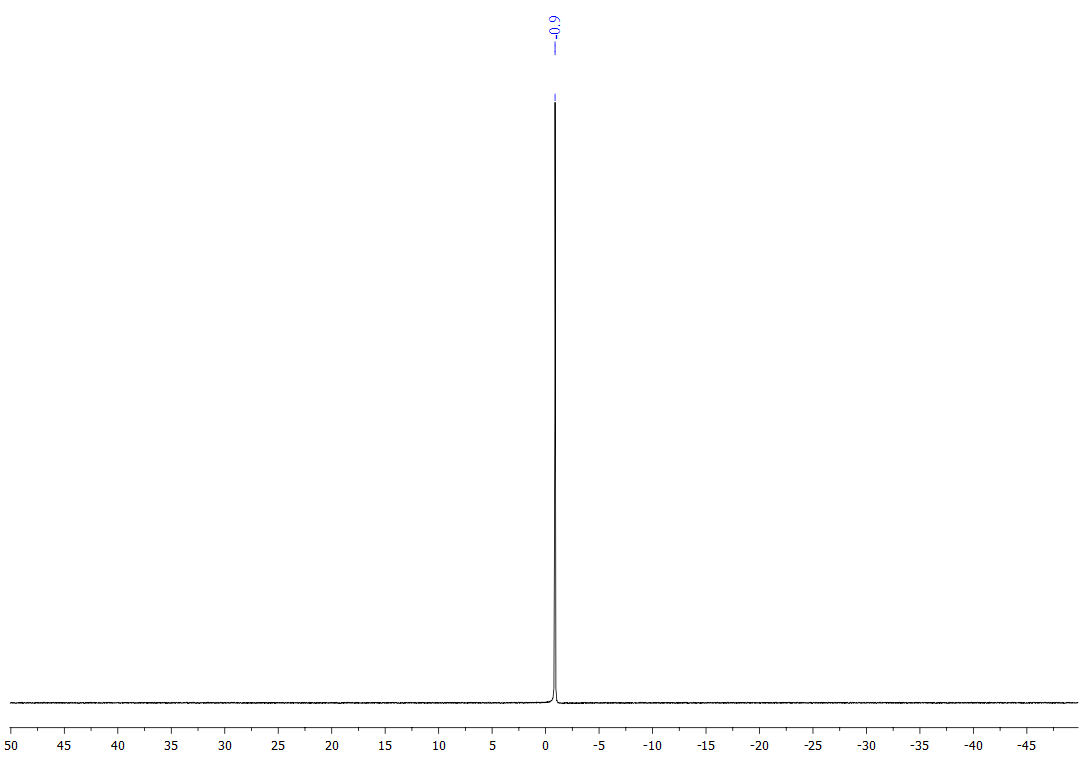
**

**S5.2 NMR spectra of pristine electrolytes**

**Figure S5.2.1** ^1^H NMR (400 MHz, (CD_3_)_2_SO, 295 K) spectrum of pristine 1 M lithium hexafluorophosphate electrolyte in EC:EMC (3:7 v/v) (LP57).


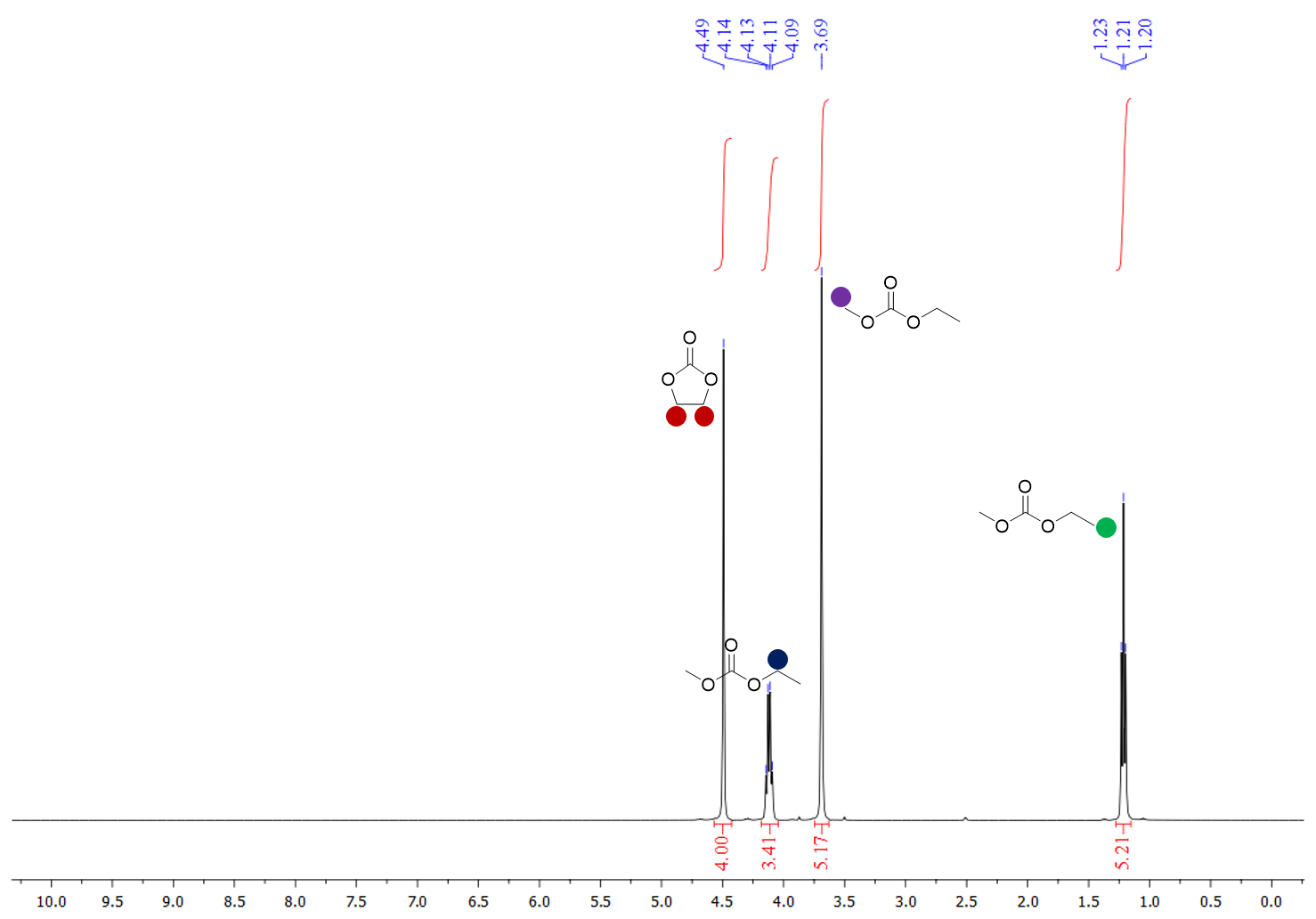


**Figure S5.2.2** ^31^P NMR (162 MHz, (CD_3_)_2_SO, 295 K) spectrum of pristine 1 M lithium hexafluorophosphate electrolyte in EC:EMC (3:7 v/v) (LP57).

**
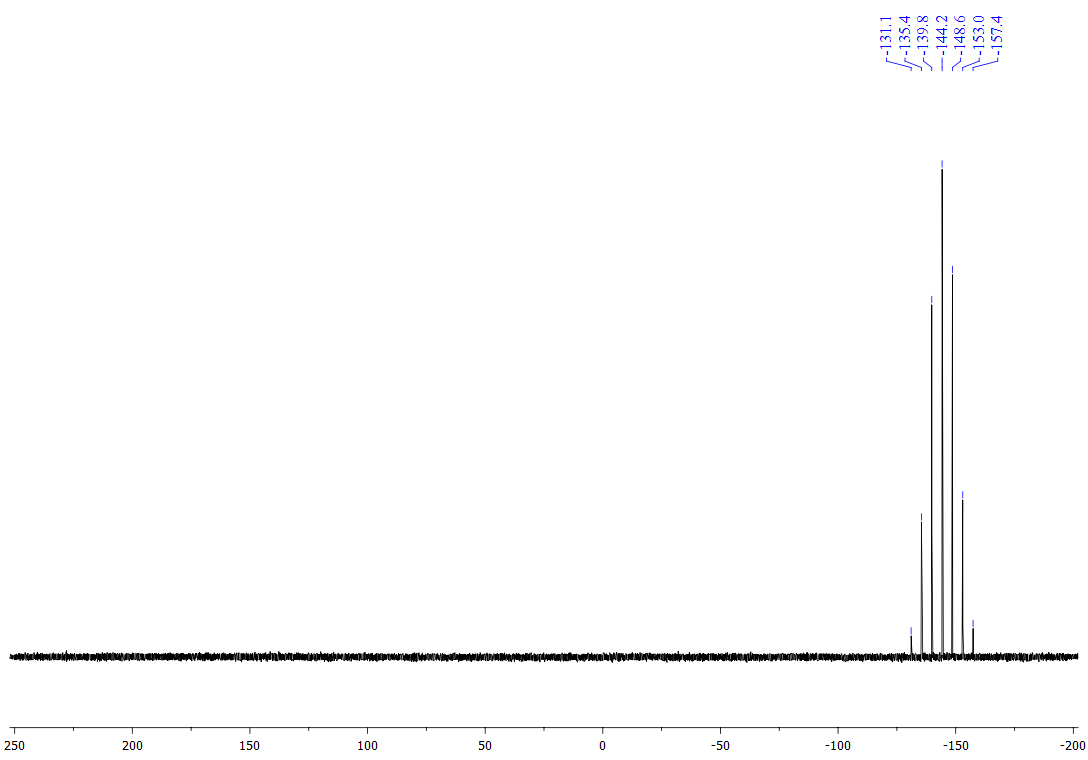
**

**Figure S5.2.3** ^19^F NMR (376 MHz, (CD_3_)_2_SO, 295 K) spectrum of pristine 1 M lithium hexafluorophosphate electrolyte in EC:EMC (3:7 v/v) (LP57).


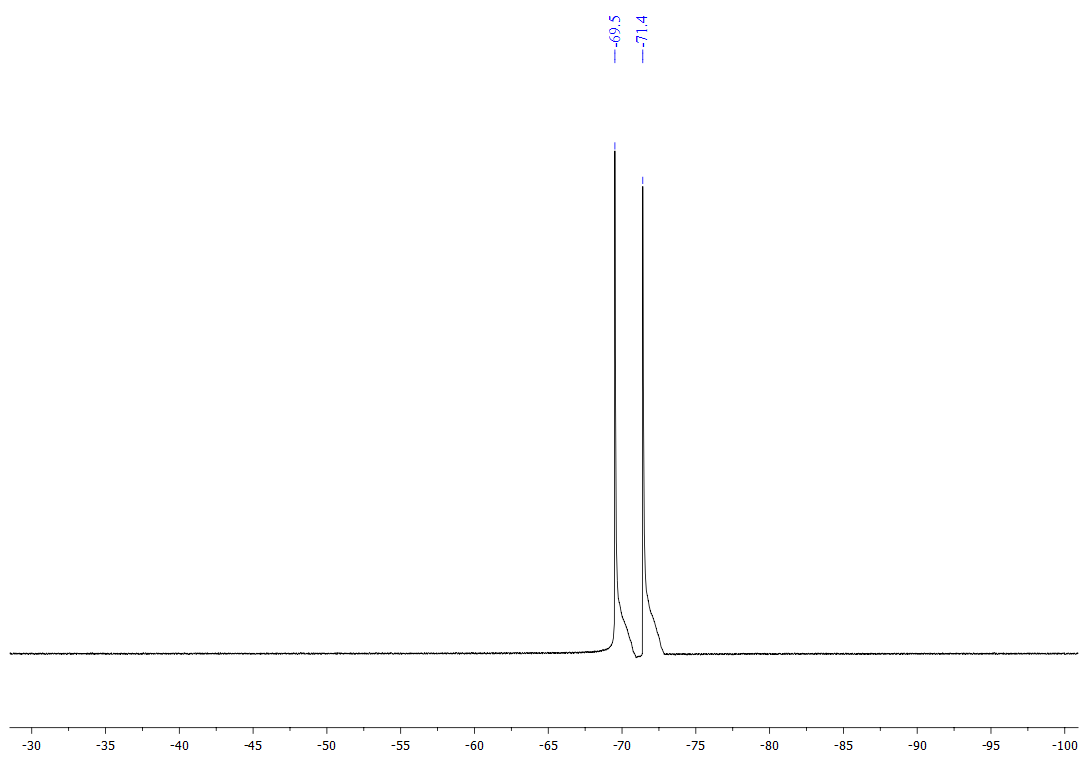


**Figure S5.2.4** ^7^Li NMR (155 MHz, (CD_3_)_2_SO, 295 K) spectrum of pristine 1 M lithium hexafluorophosphate electrolyte in EC:EMC (3:7 v/v) (LP57).


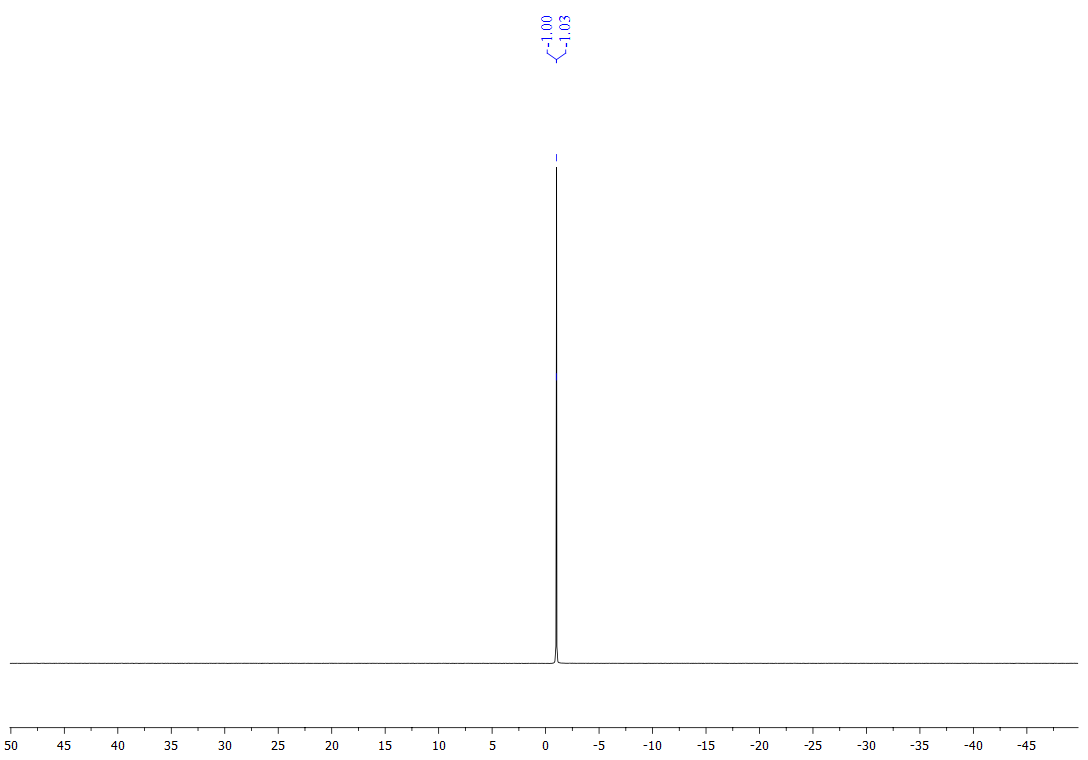


**Figure S5.2.5** ^1^H NMR (400 MHz, (CD_3_)_2_SO, 295 K) spectrum of pristine 1 M lithium bis(perfluorinated pinacolato)borate, Li[B(pp)_2_]·DME, electrolyte in EC:EMC (3:7 v/v).

**
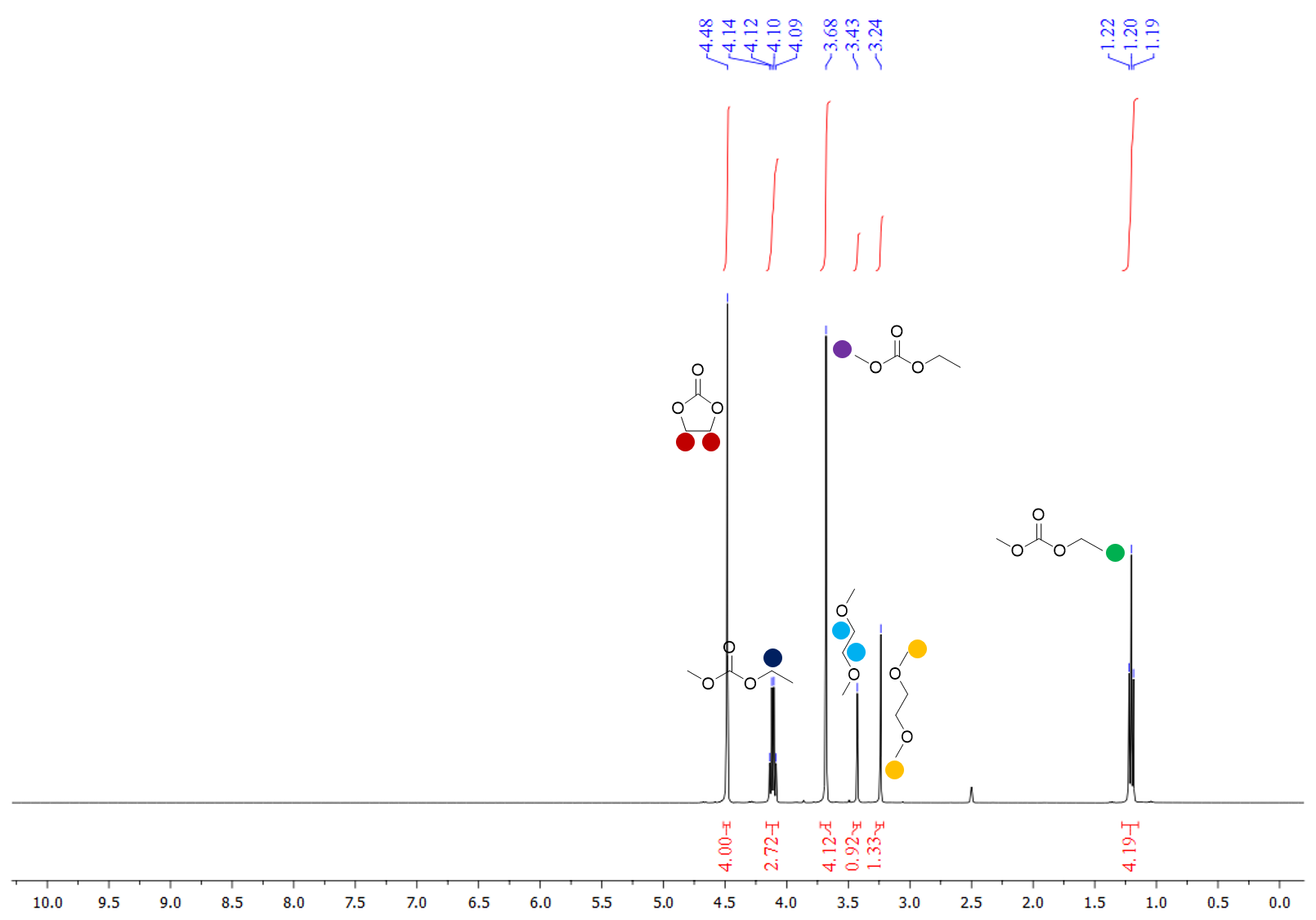
**

**Figure S5.2.6** ^11^B NMR (128 MHz, (CD_3_)_2_SO, 295 K) spectrum of pristine 1 M lithium bis(perfluorinated pinacolato)borate, Li[B(pp)_2_]·DME, electrolyte in EC:EMC (3:7 v/v).

**
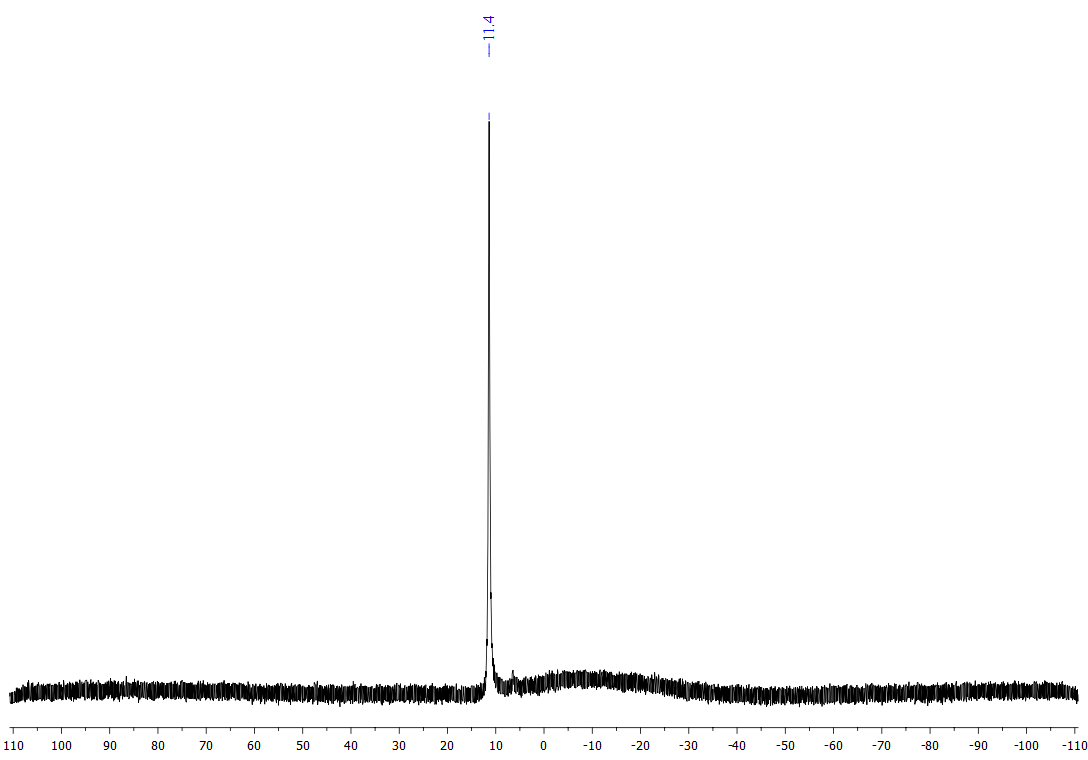
**

**Figure S5.2.7** ^19^F NMR (376 MHz, (CD_3_)_2_SO, 295 K) spectrum of pristine 1 M lithium bis(perfluorinated pinacolato)borate, Li[B(pp)_2_]·DME, electrolyte in EC:EMC (3:7 v/v).

**
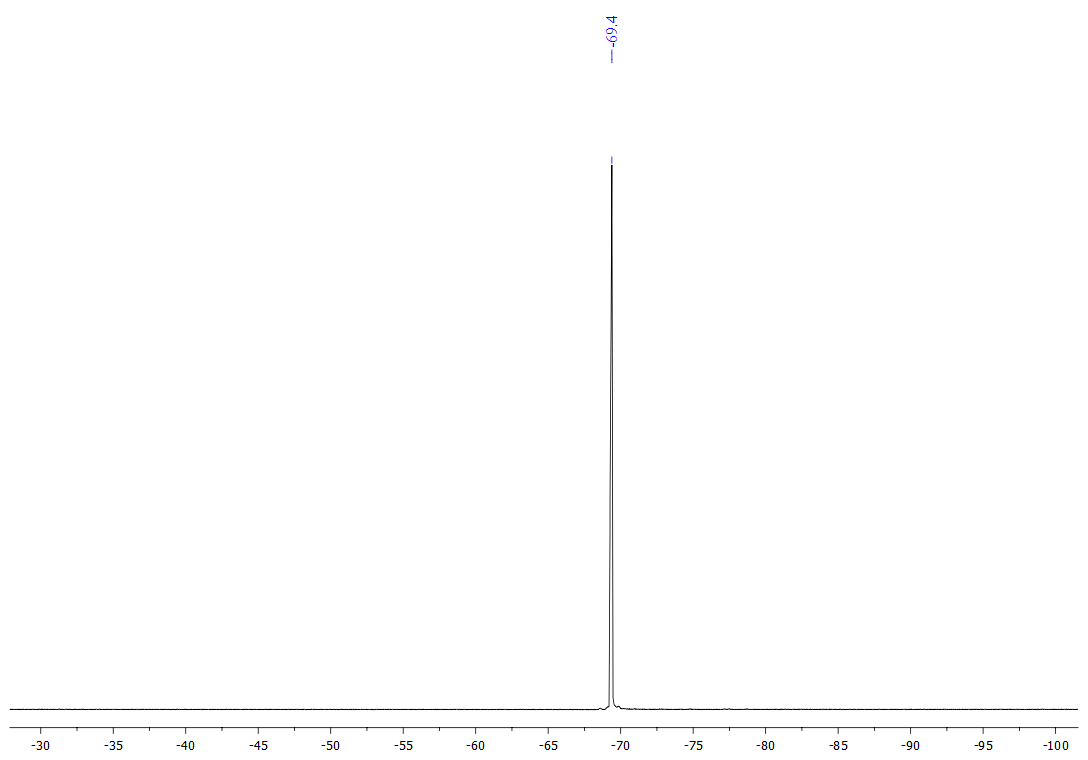
**

**Figure S5.2.8** ^7^Li NMR (155 MHz, (CD_3_)_2_SO, 295 K) spectrum of pristine 1 M lithium bis(perfluorinated pinacolato)borate, Li[B(pp)_2_]·DME, electrolyte in EC:EMC (3:7 v/v).

**
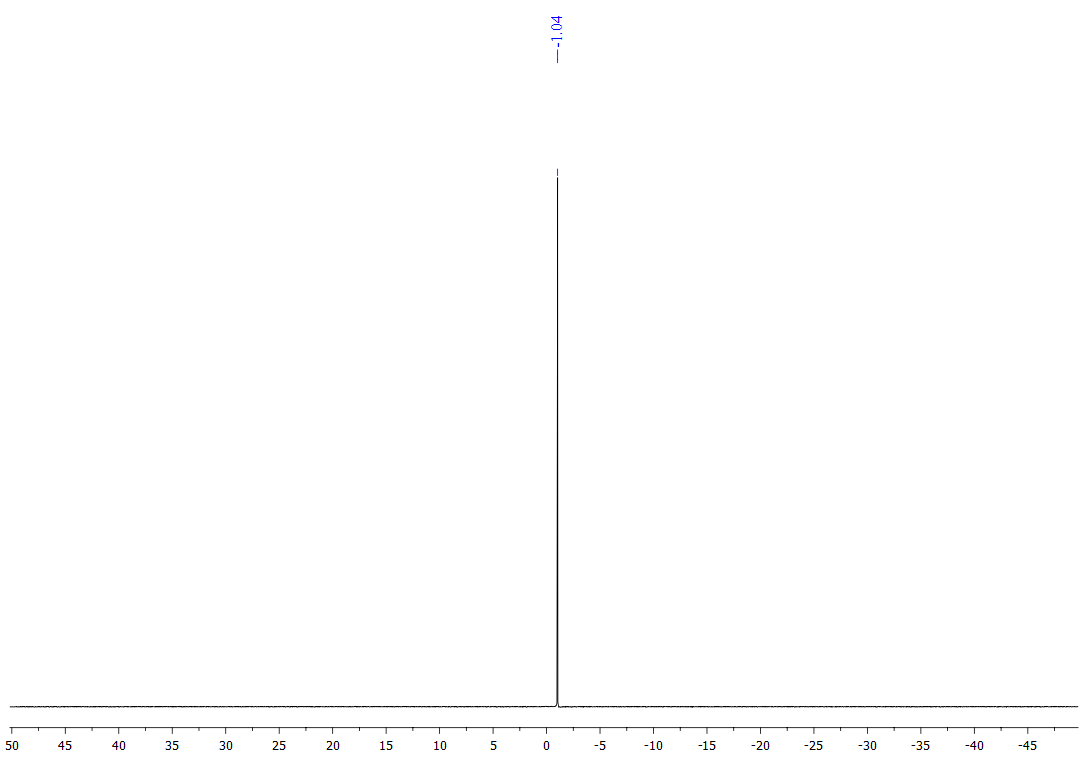
**

**Figure S5.2.9** ^1^H NMR (400 MHz, (CD_3_)_2_SO, 295 K) spectrum of pristine 0.2 M lithium bis(perfluorinated pinacolato)borate, Li[B(pp)_2_], electrolyte in EC:EMC (3:7 v/v).

**
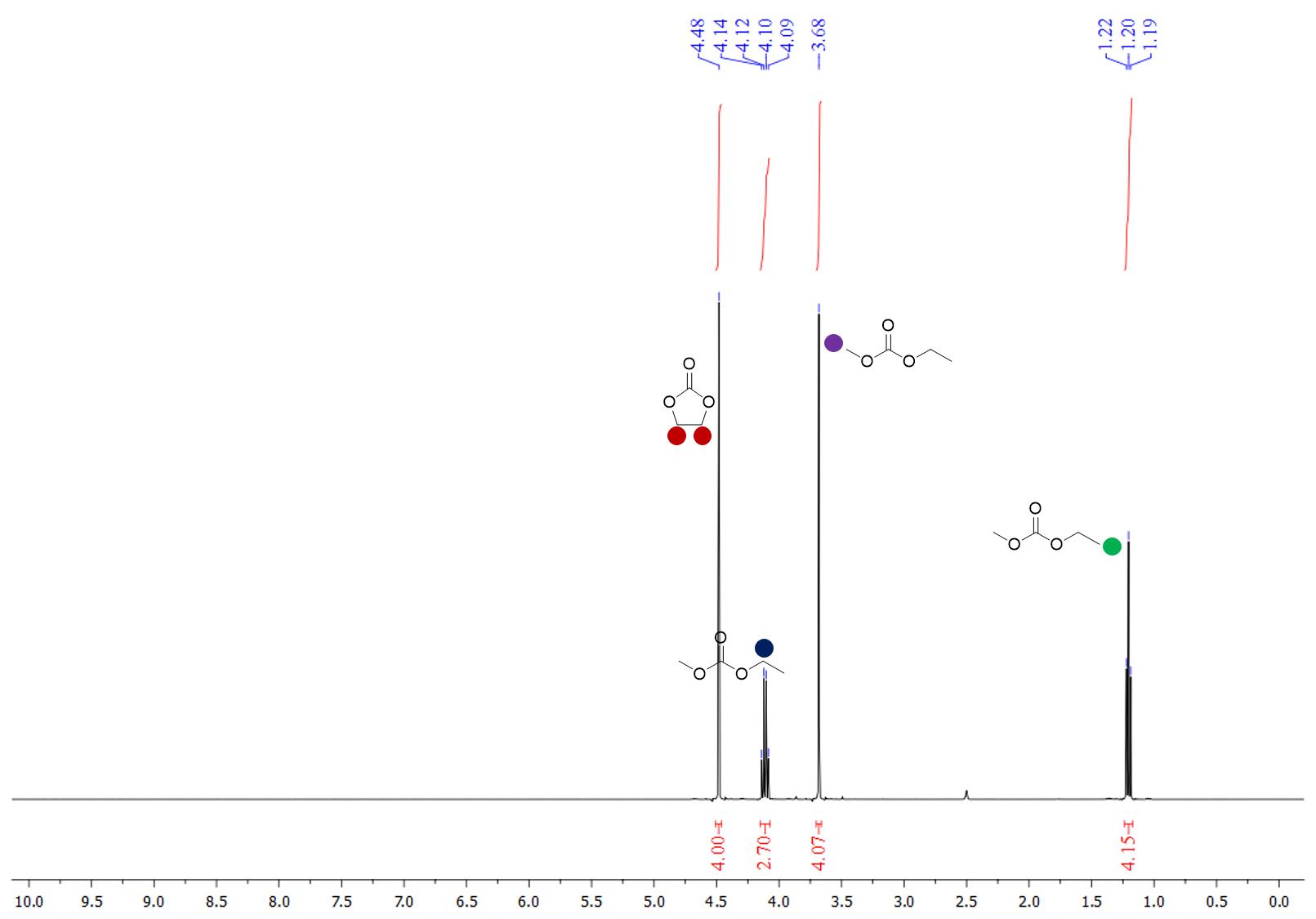
**

**Figure S5.2.10** ^11^B NMR (128 MHz, (CD_3_)_2_SO, 295 K) spectrum of pristine 0.2 M lithium bis(perfluorinated pinacolato)borate, Li[B(pp)_2_], electrolyte in EC:EMC (3:7 v/v).


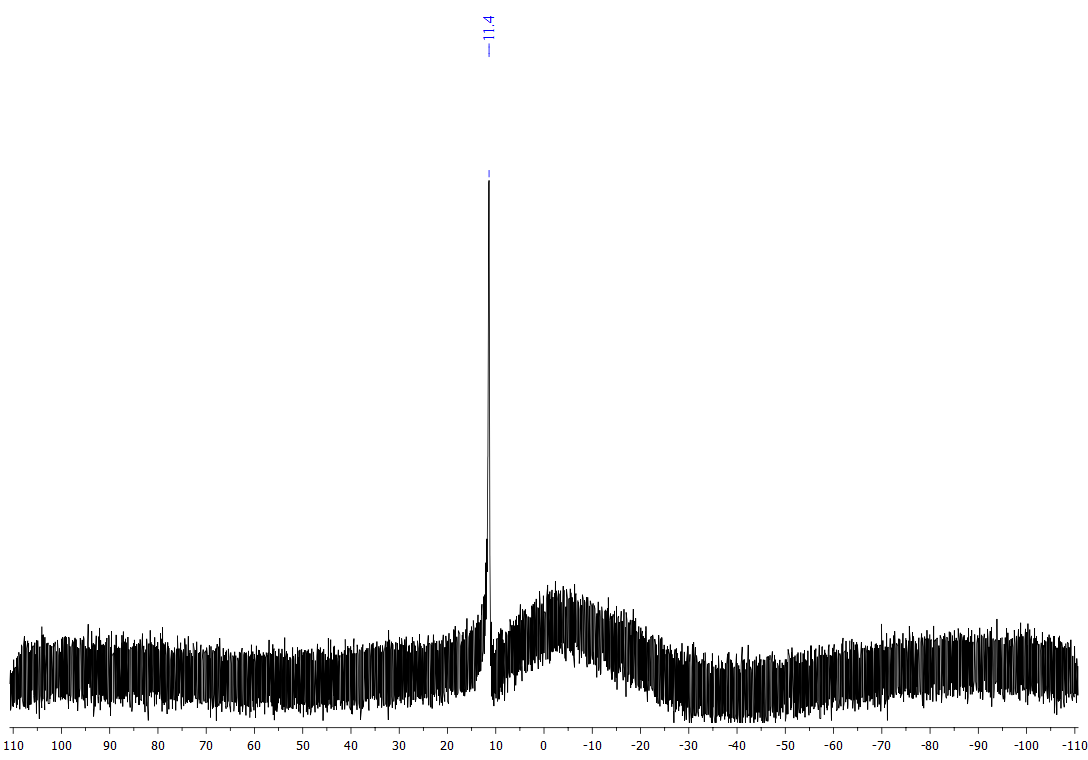


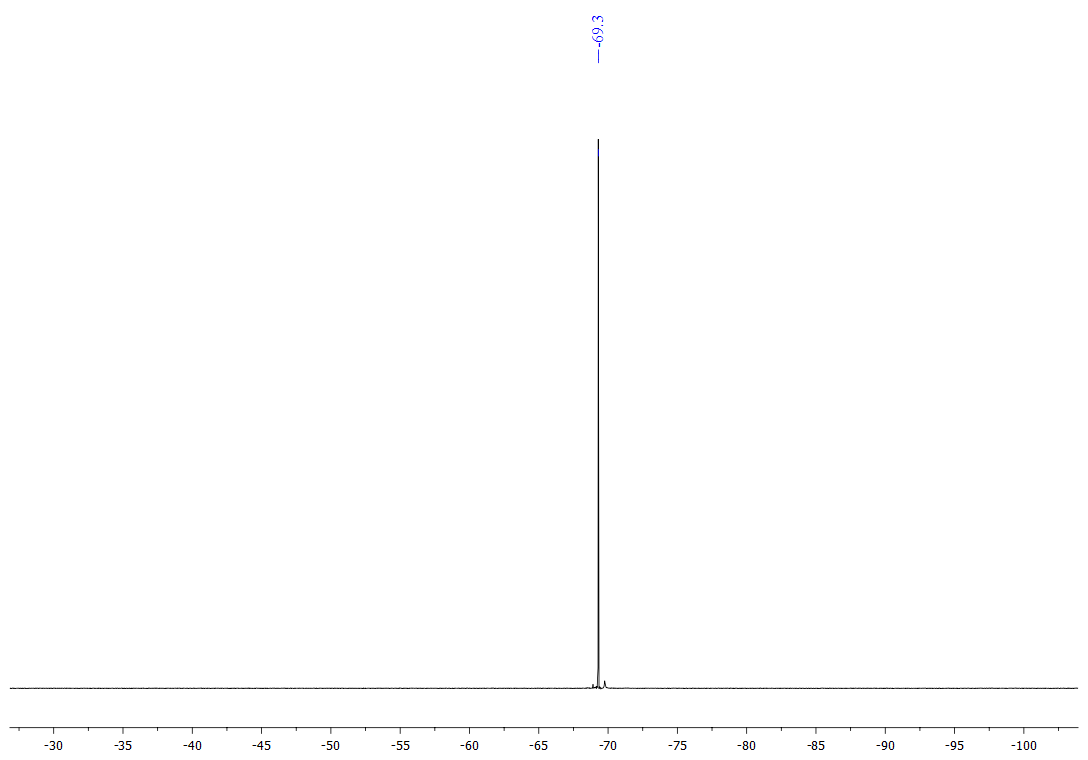
**Figure S5.2.11** ^19^F NMR (376 MHz, (CD_3_)_2_SO, 295 K) spectrum of pristine 0.2 M lithium bis(perfluorinated pinacolato)borate, Li[B(pp)_2_], electrolyte in EC:EMC (3:7 v/v).

^^**Figure S5.2.12** ^7^Li NMR (155 MHz, (CD_3_)_2_SO, 295 K) spectrum of pristine 0.2 M lithium bis(perfluorinated pinacolato)borate, Li[B(pp)_2_], electrolyte in EC:EMC (3:7 v/v).

^
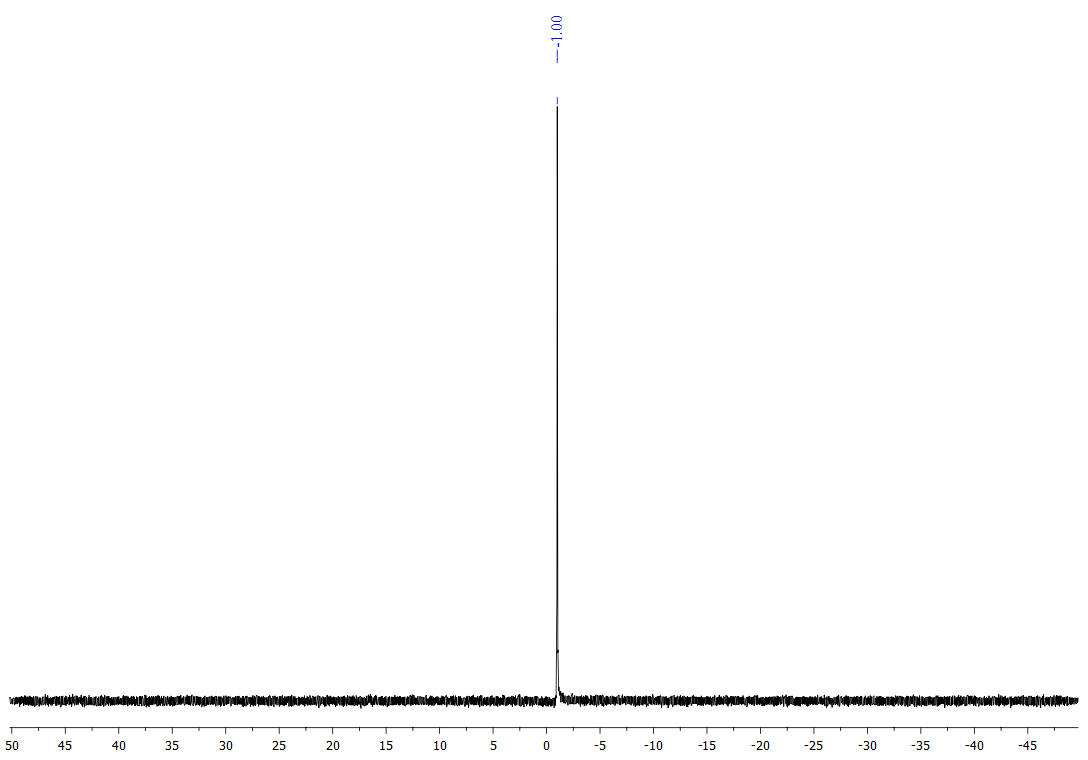
^

**S5.3 NMR spectra of post lithium-ion cycling electrolyte at rate C/3**


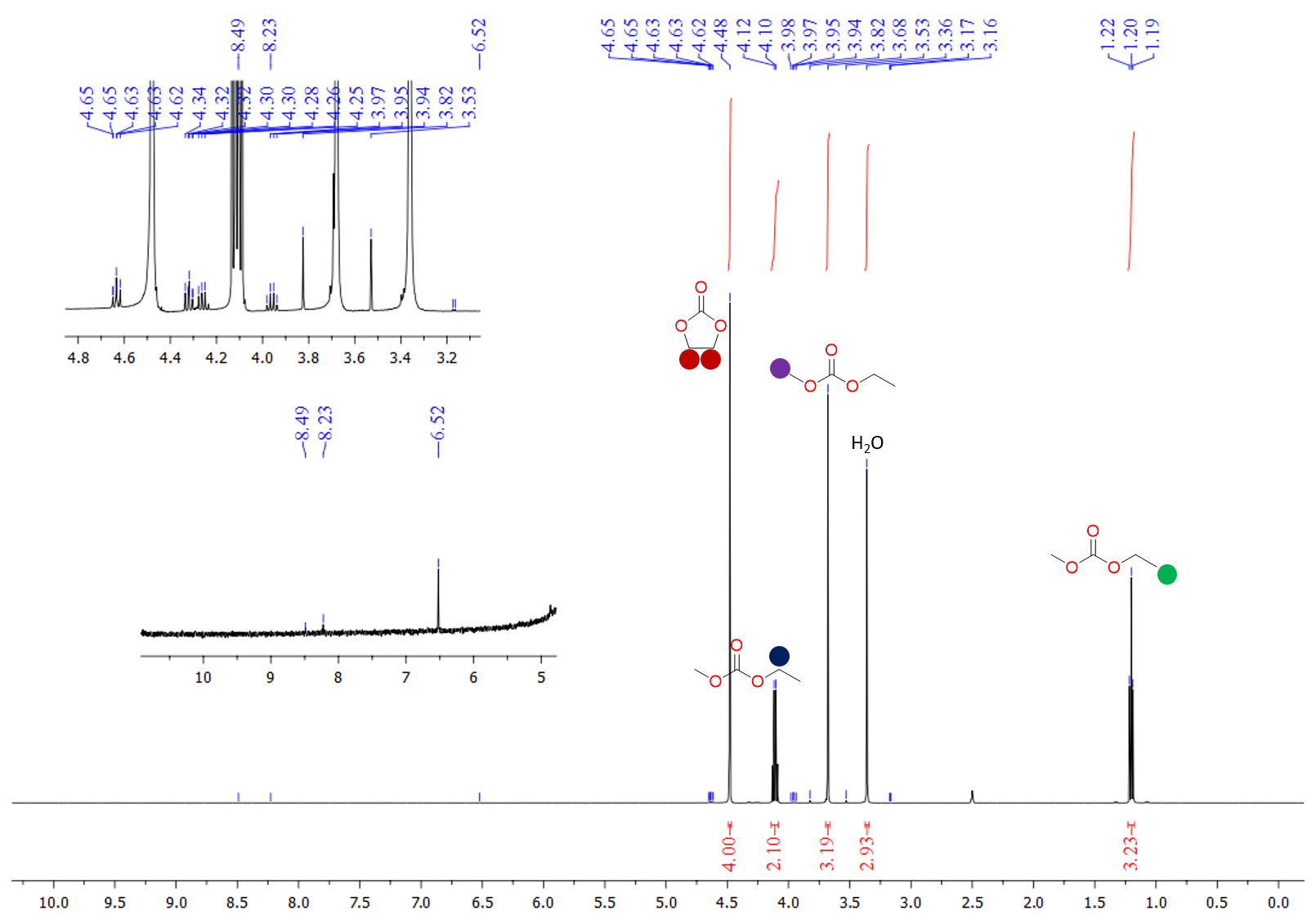
**Figure S5.3.1** ^1^H NMR (500 MHz, (CD_3_)_2_SO, 295 K) spectrum of 1 M lithium hexafluorophosphate electrolyte in EC:EMC (3:7 v/v) (LP57) after 40 cycles at rate C/3.

**Figure S5.3.2** ^31^P NMR (162 MHz, (CD_3_)_2_SO, 295 K) spectrum of 1 M lithium hexafluorophosphate electrolyte in EC:EMC (3:7 v/v) (LP57) after 40 cycles at rate C/3.


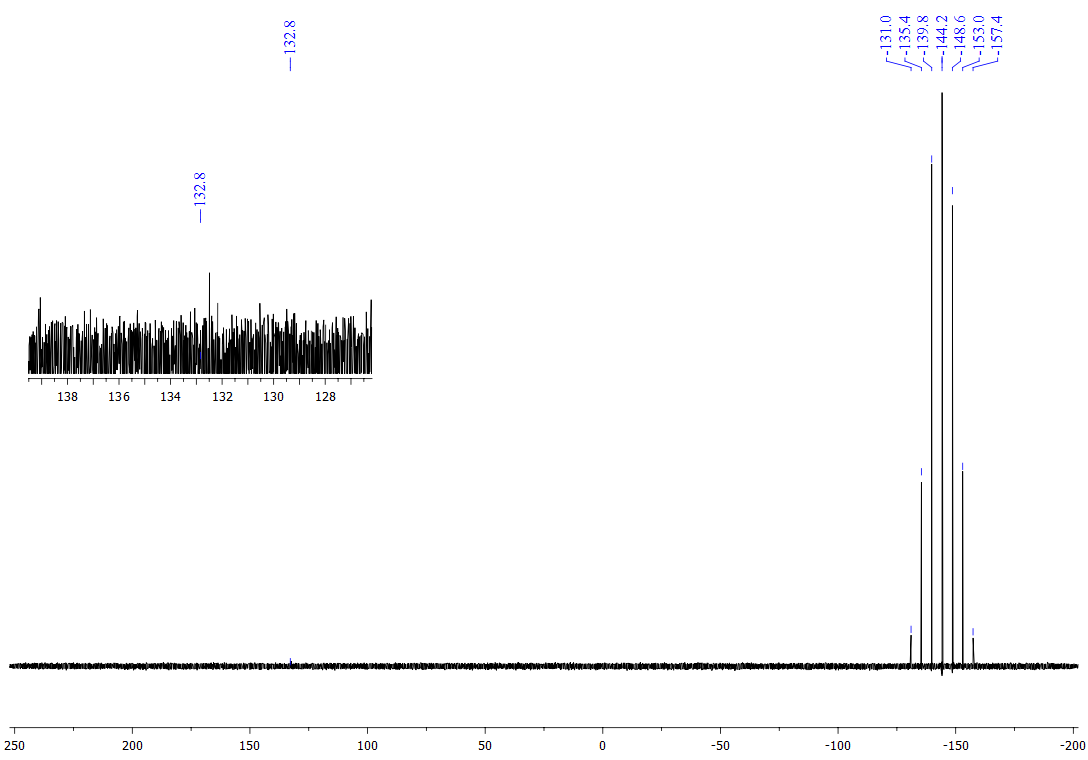


**Figure S5.3.3** ^19^F NMR (471 MHz, (CD_3_)_2_SO, 295 K) spectrum of 1 M lithium hexafluorophosphate electrolyte in EC:EMC (3:7 v/v) (LP57) after 40 cycles at rate C/3.


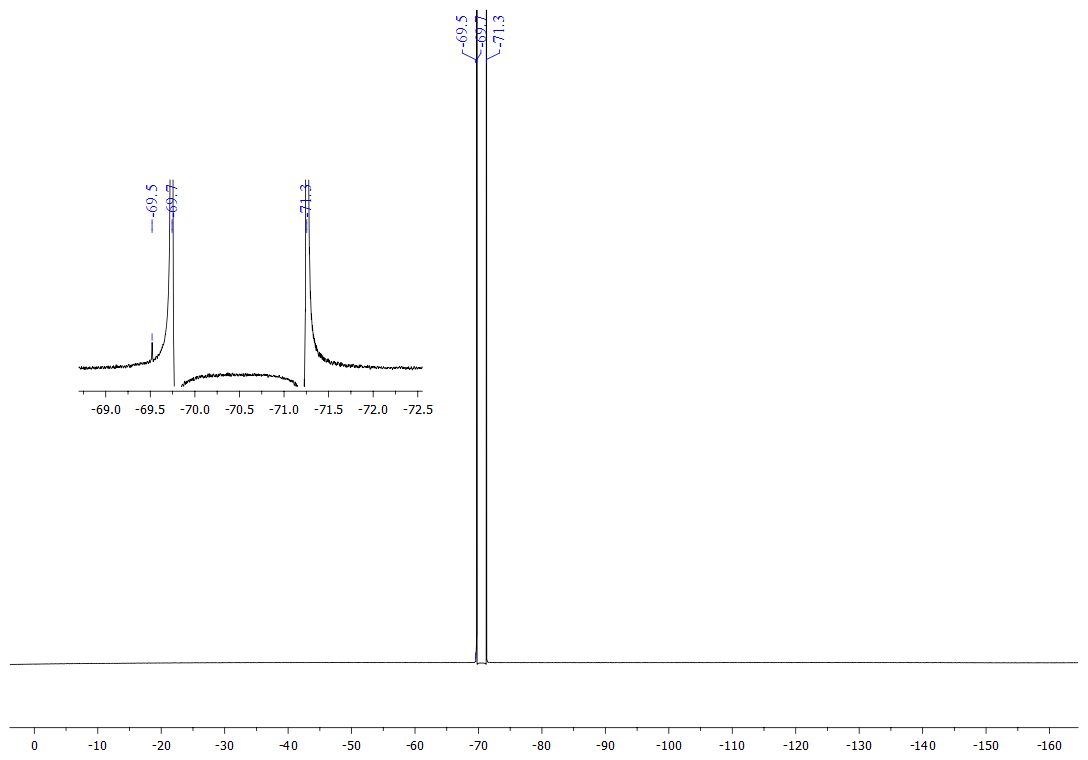


**Figure S5.3.4** ^7^Li NMR (155 MHz, (CD_3_)_2_SO, 295 K) spectrum of 1 M lithium hexafluorophosphate electrolyte in EC:EMC (3:7 v/v) (LP57) after 40 cycles at rate C/3.


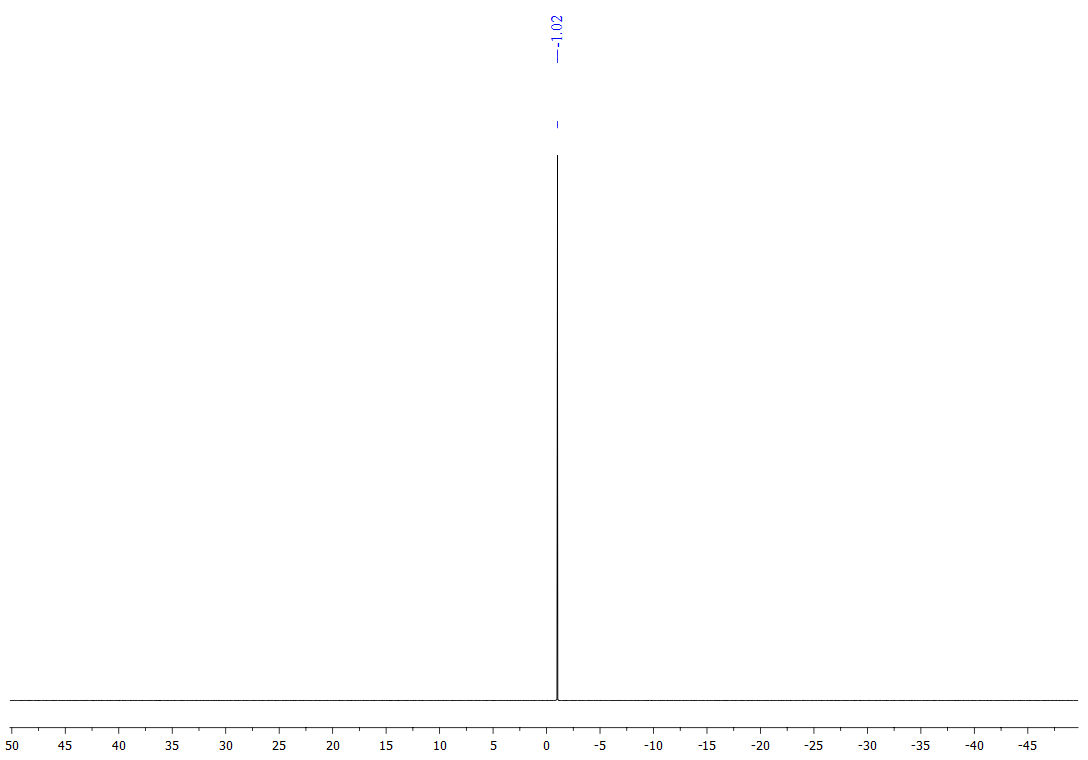


**
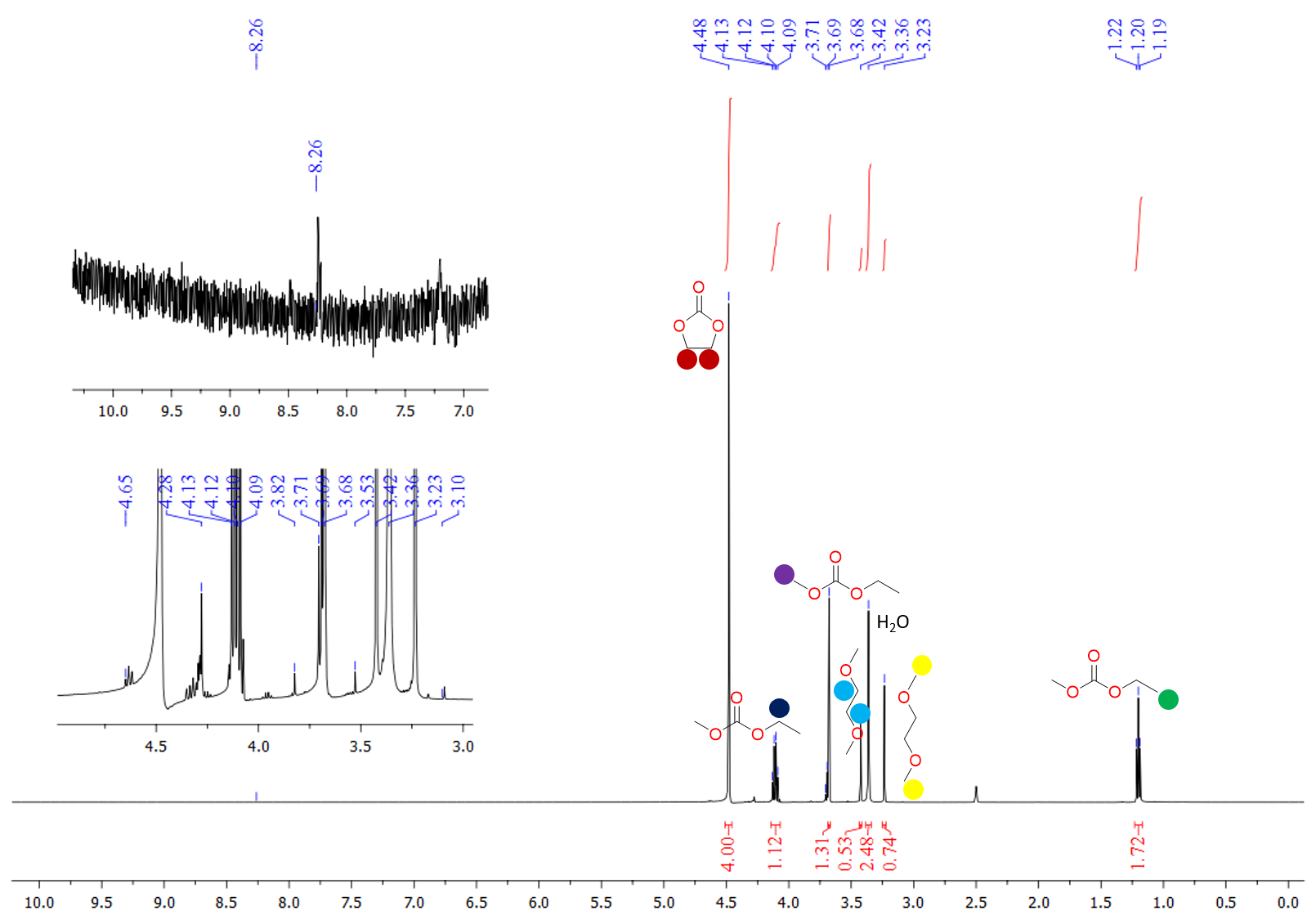
Figure S5.3.5** ^1^H NMR (500 MHz, (CD_3_)_2_SO, 295 K) spectrum of 1 M lithium bis(perfluorinated pinacolato)borate, Li[B(pp)_2_]·DME, electrolyte in EC:EMC (3:7 v/v) after 45 cycles at rate C/3.

**Figure S5.3.6** ^11^B NMR (160 MHz, (CD_3_)_2_SO, 295 K) spectrum of 1 M lithium bis(perfluorinated pinacolato)borate, Li[B(pp)_2_]·DME, electrolyte in EC:EMC (3:7 v/v) after 45 cycles at rate C/3.

**
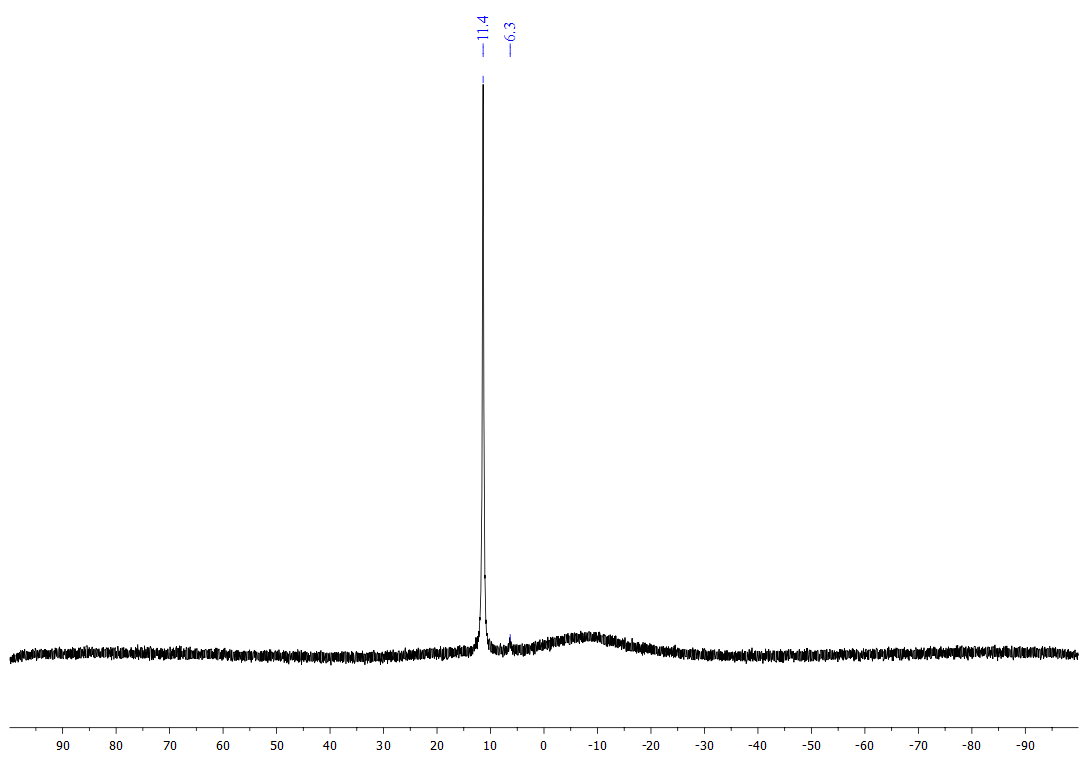
**

**Figure S5.3.7** ^19^F NMR (471 MHz, (CD_3_)_2_SO, 295 K) spectrum of 1 M lithium bis(perfluorinated pinacolato)borate, Li[B(pp)_2_]·DME, electrolyte in EC:EMC (3:7 v/v) after 45 cycles at rate C/3.

**
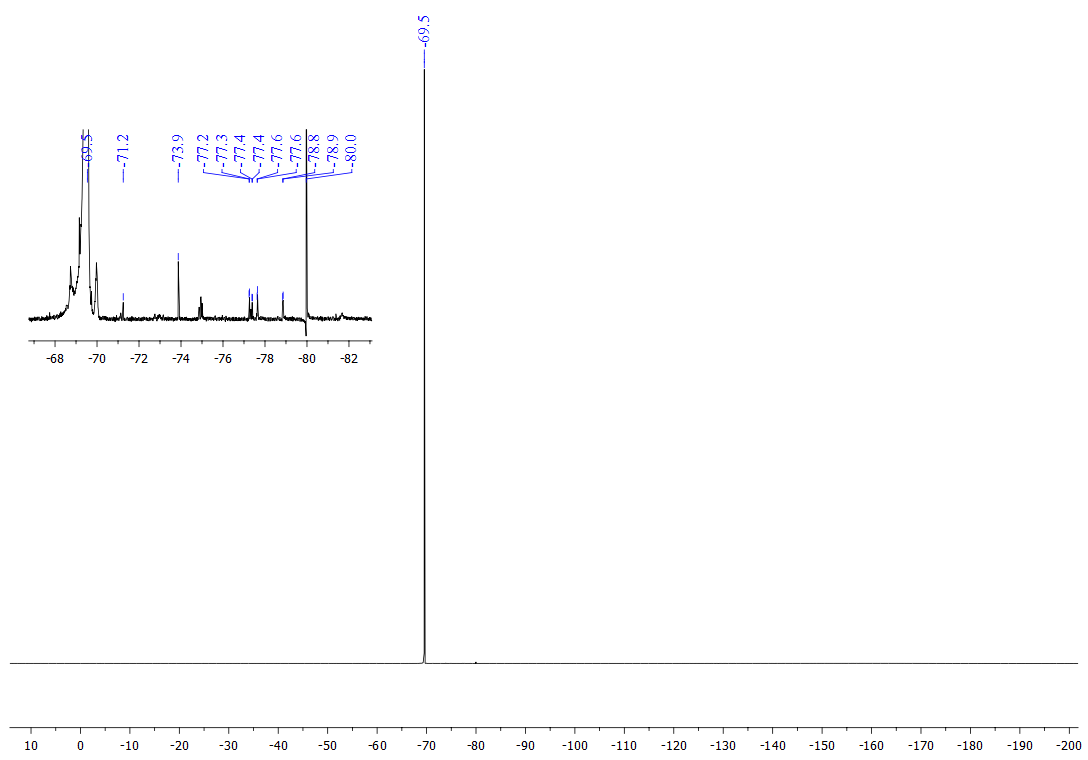
**

**Figure S5.3.8** ^7^Li NMR (155 MHz, (CD_3_)_2_SO, 295 K) spectrum of 1 M lithium bis(perfluorinated pinacolato)borate, Li[B(pp)_2_]·DME, electrolyte in EC:EMC (3:7 v/v) after 45 cycles at rate C/3.

**
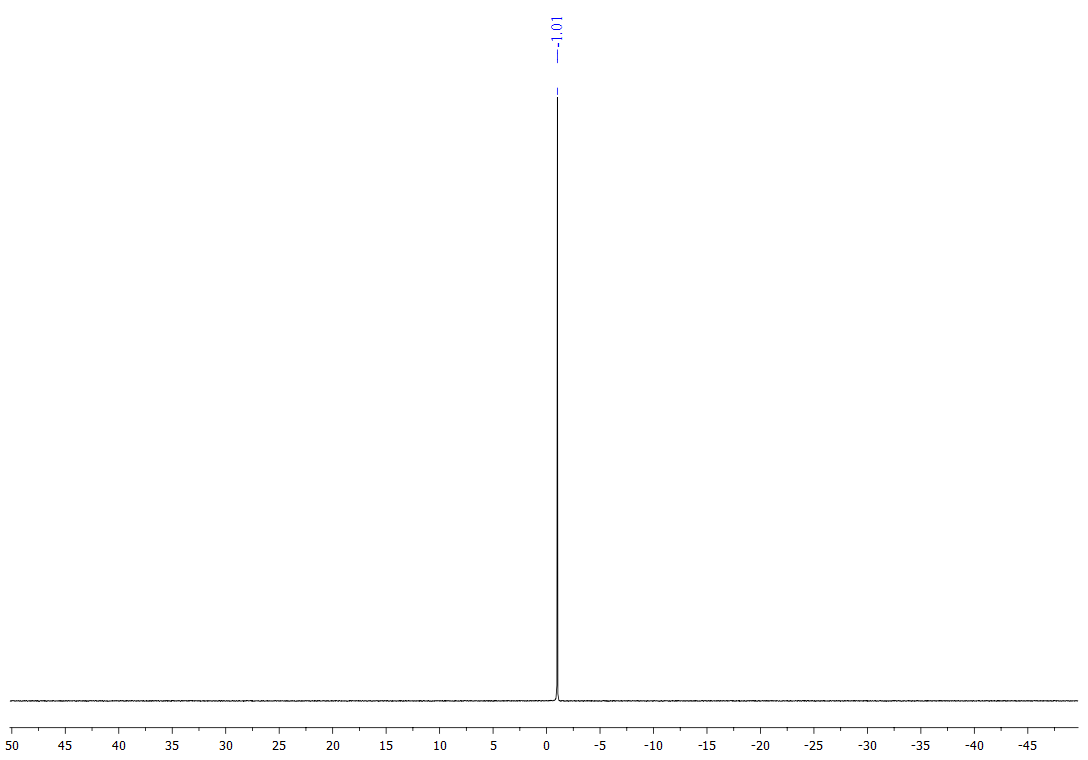
**

**Figure S5.3.9** ^1^H NMR (500 MHz, (CD_3_)_2_SO, 295 K) spectrum of 0.2 M lithium bis(perfluorinated pinacolato)borate, Li[B(pp)_2_], electrolyte in EC:EMC (3:7 v/v) after 45 cycles at rate C/3.


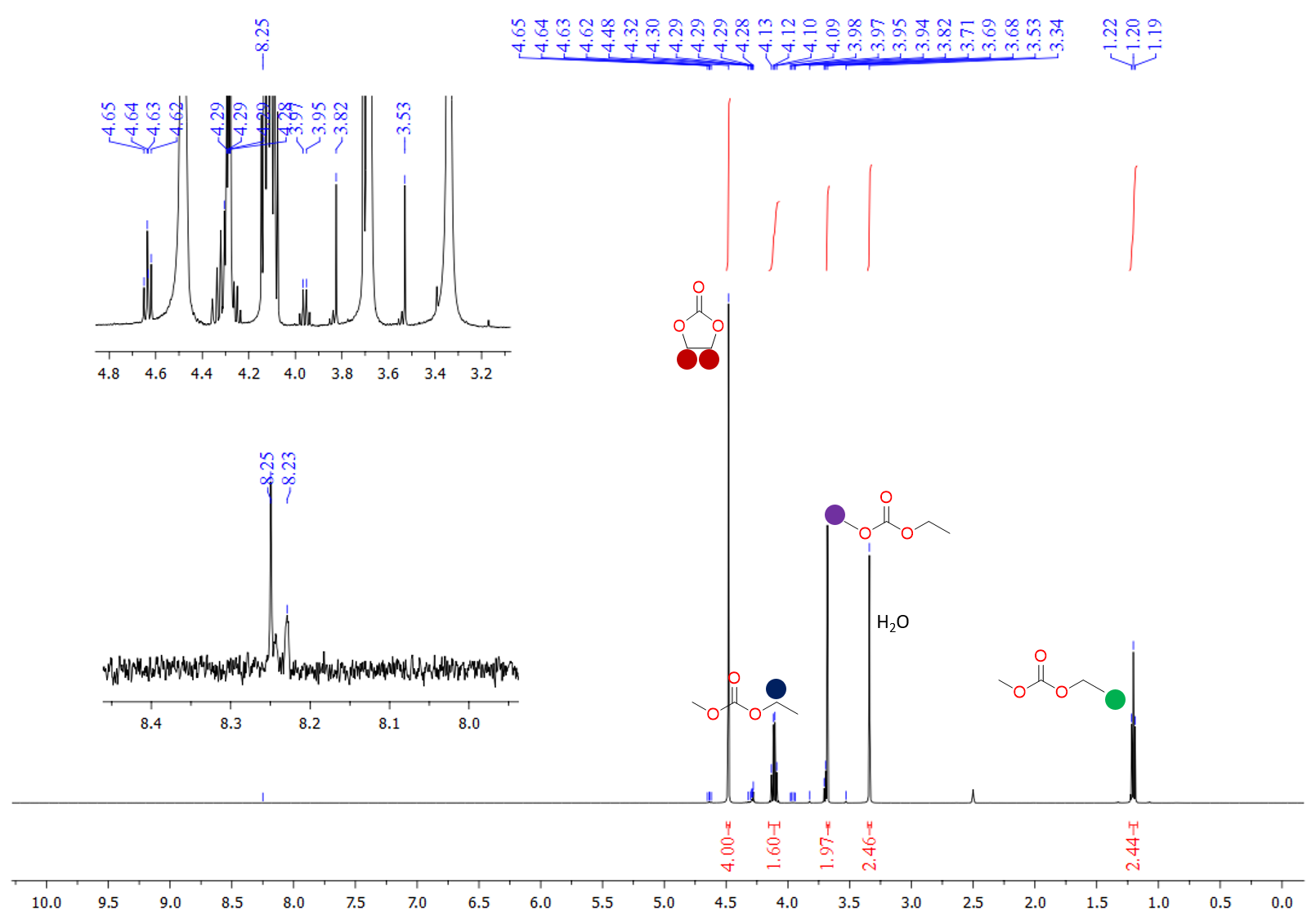


**Figure S5.3.10** ^11^B NMR (128 MHz, (CD_3_)_2_SO, 295 K) spectrum of 0.2 M lithium bis(perfluorinated pinacolato)borate, Li[B(pp)_2_], electrolyte in EC:EMC (3:7 v/v) after 45 cycles at rate C/3.


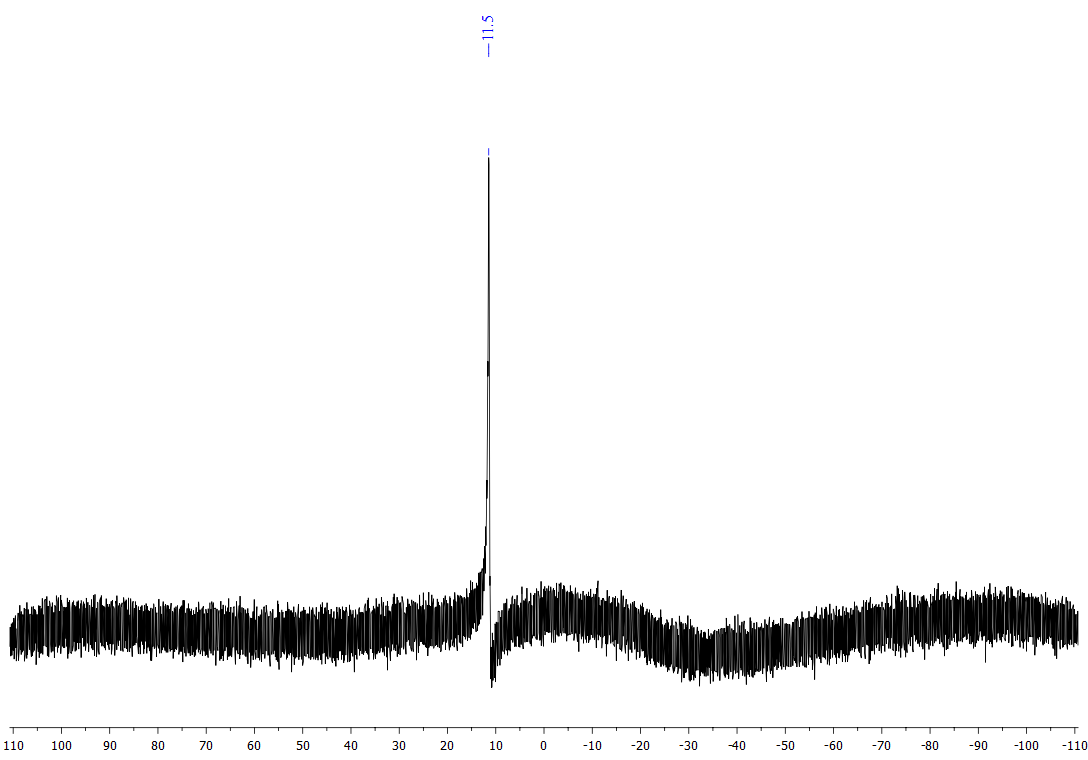


**Figure S5.3.11** ^19^F NMR (471 MHz, (CD_3_)_2_SO, 295 K) spectrum of 0.2 M lithium bis(perfluorinated pinacolato)borate, Li[B(pp)_2_], electrolyte in EC:EMC (3:7 v/v) after 45 cycles at rate C/3.


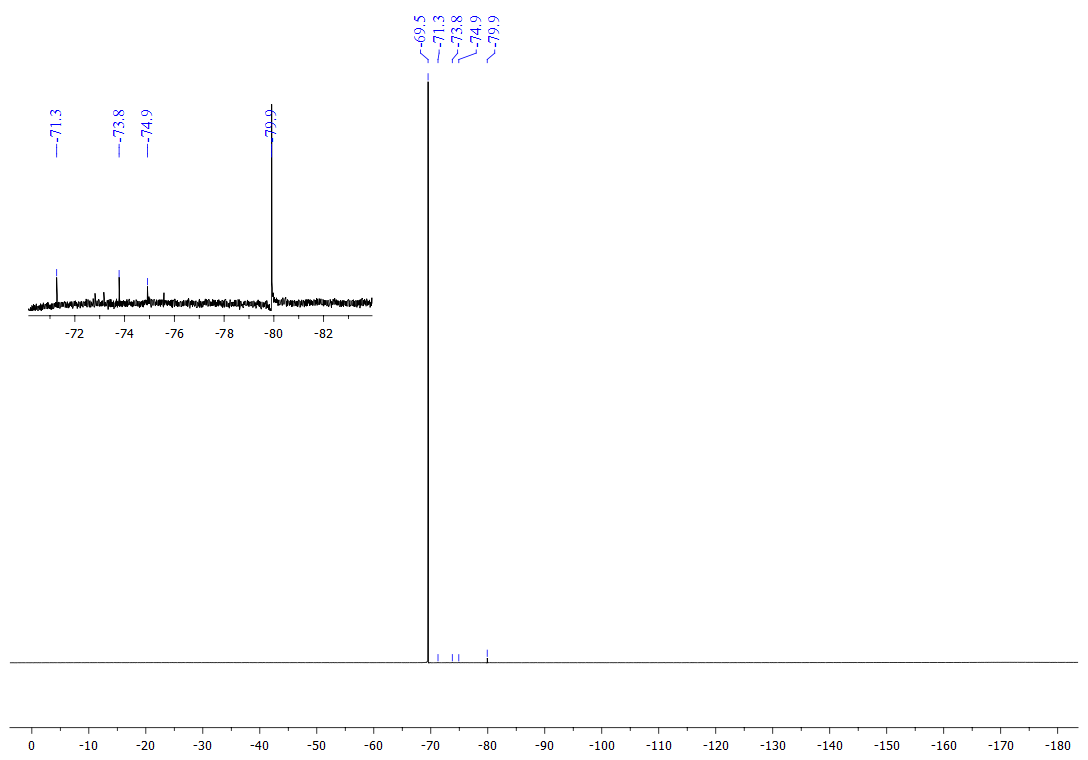


**Figure S5.3.12** ^7^Li NMR (155 MHz, (CD_3_)_2_SO, 295 K) spectrum of 0.2 M lithium bis(perfluorinated pinacolato)borate, Li[B(pp)_2_], electrolyte in EC:EMC (3:7 v/v) after 45 cycles at rate C/3.

**
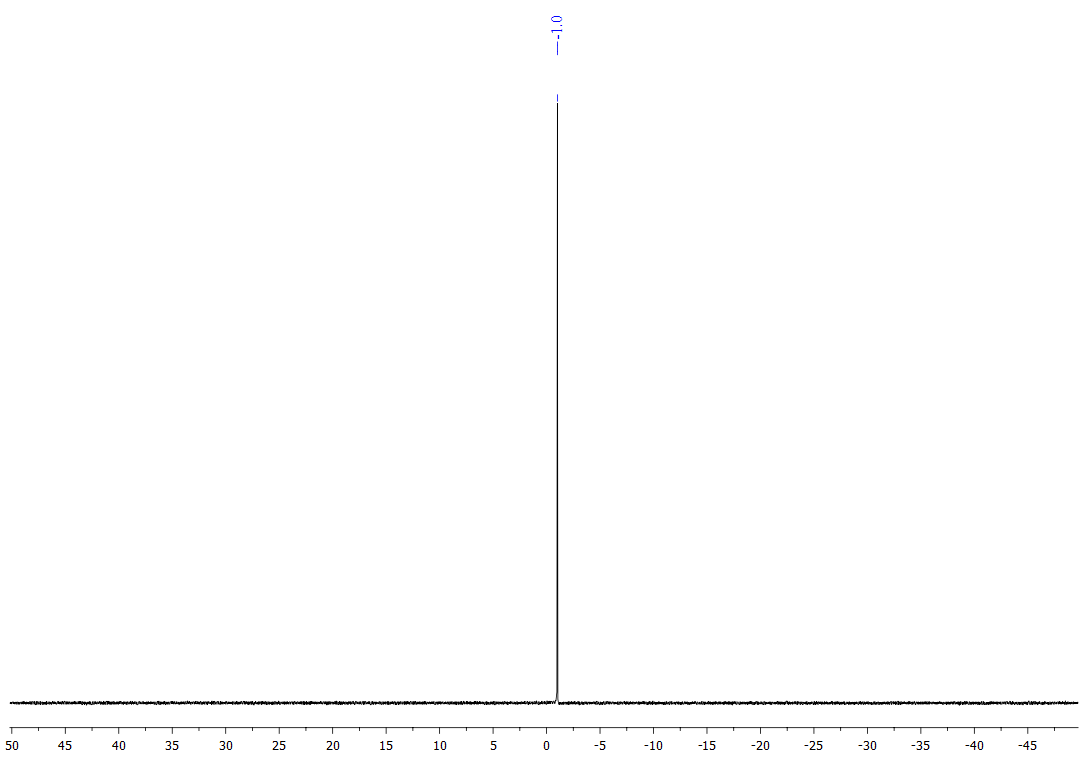
**

**S5.4 NMR spectra of air exposed lithium borate salts**

**
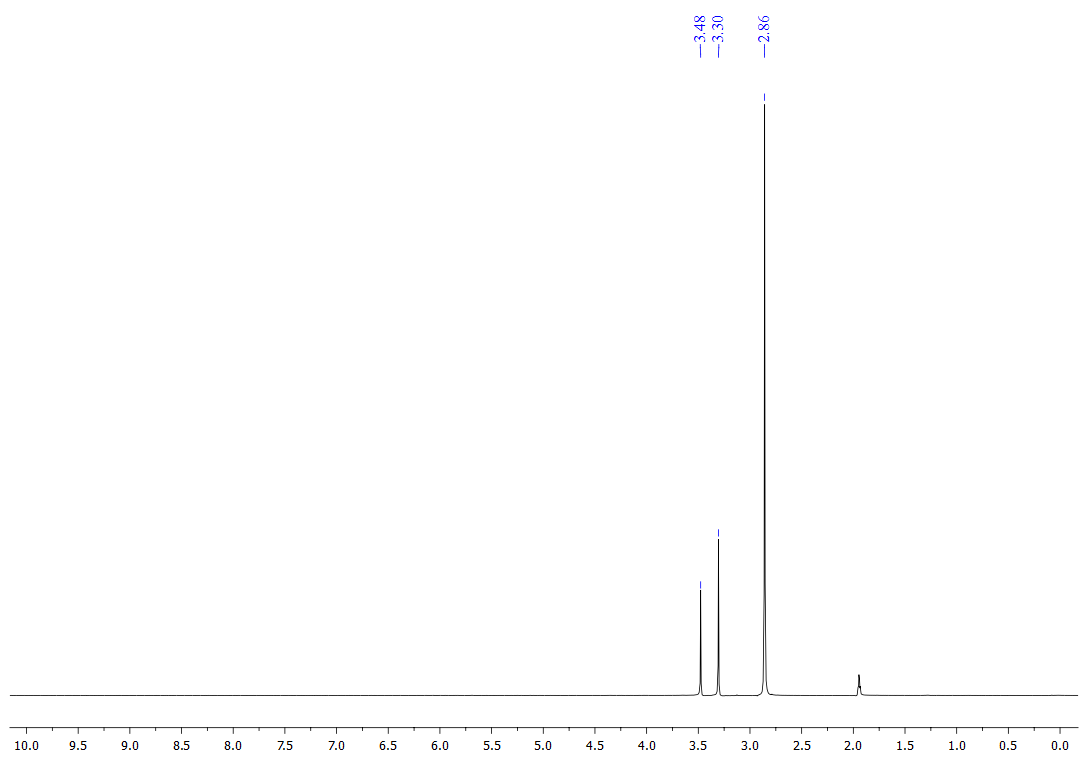
Figure S5.4.1** ^1^H NMR (400 MHz, CD_3_CN, 295 K) spectrum of lithium bis(perfluorinated pinacolato)borate, Li[B(pp)_2_]·DME after 5 weeks air exposure.

**
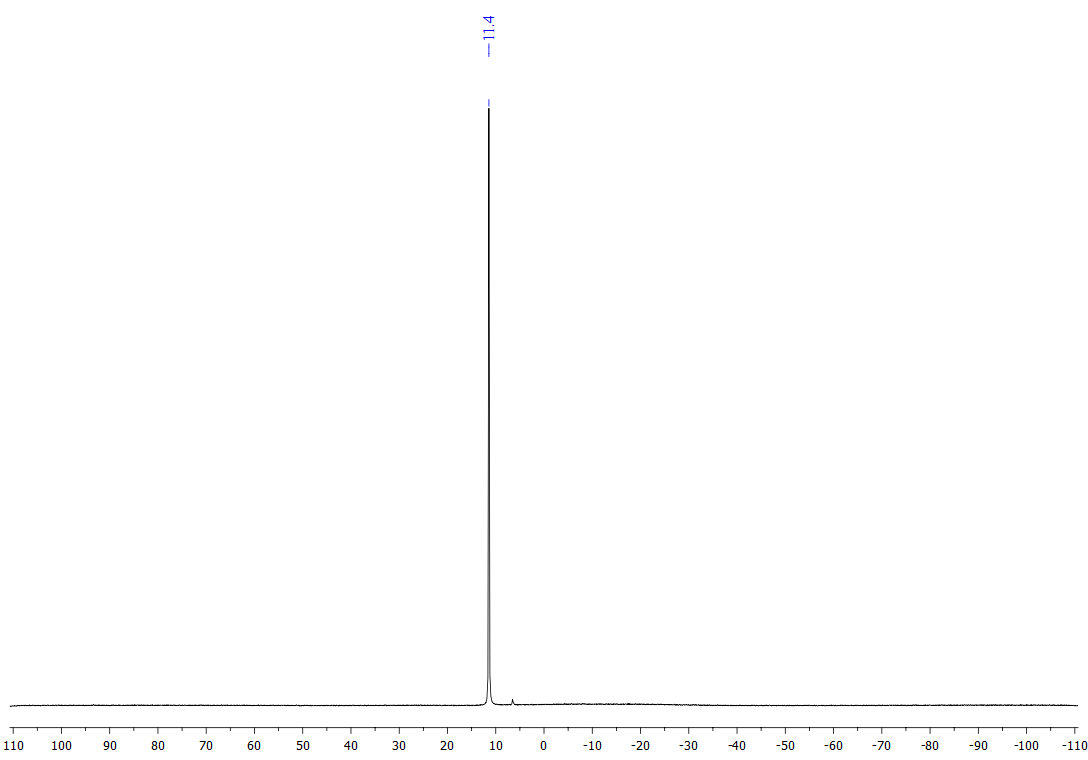
Figure S5.4.2** ^11^B NMR (128 MHz, CD_3_CN, 295 K) spectrum of lithium bis(perfluorinated pinacolato)borate, Li[B(pp)_2_]·DME after 5 weeks air exposure.

**
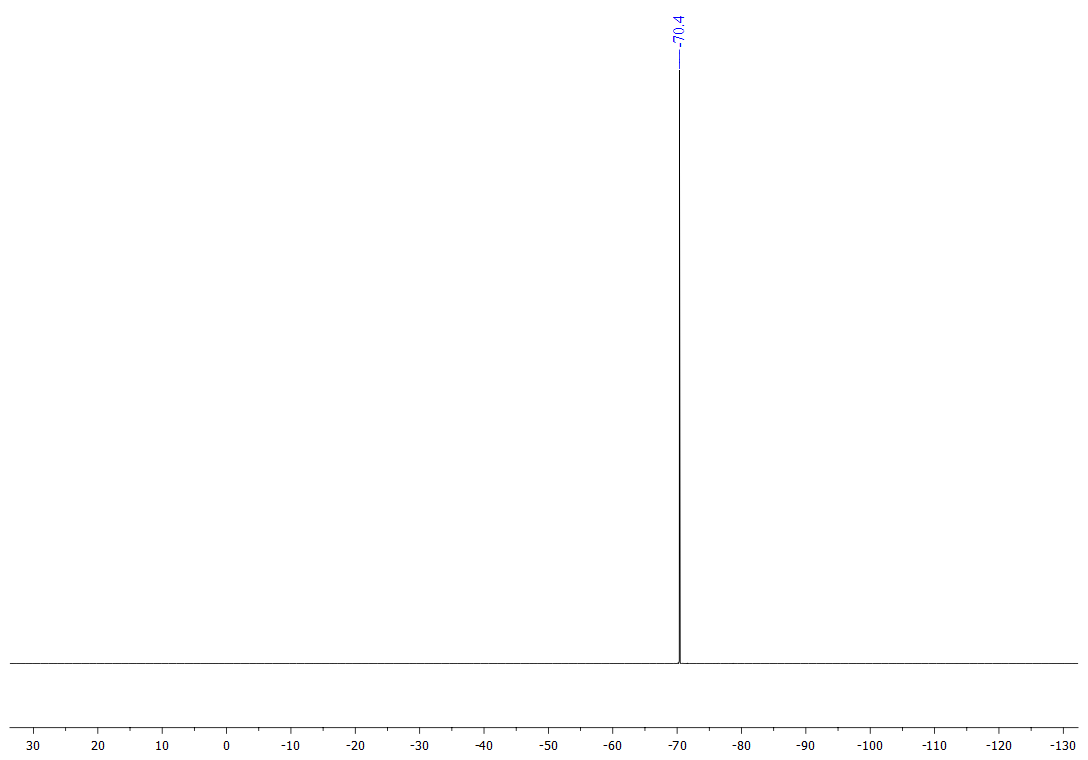
Figure S5.4.3** ^19^F NMR (376 MHz, CD_3_CN, 295 K) spectrum of lithium bis(perfluorinated pinacolato)borate, Li[B(pp)_2_]·DME after 5 weeks air exposure.

**
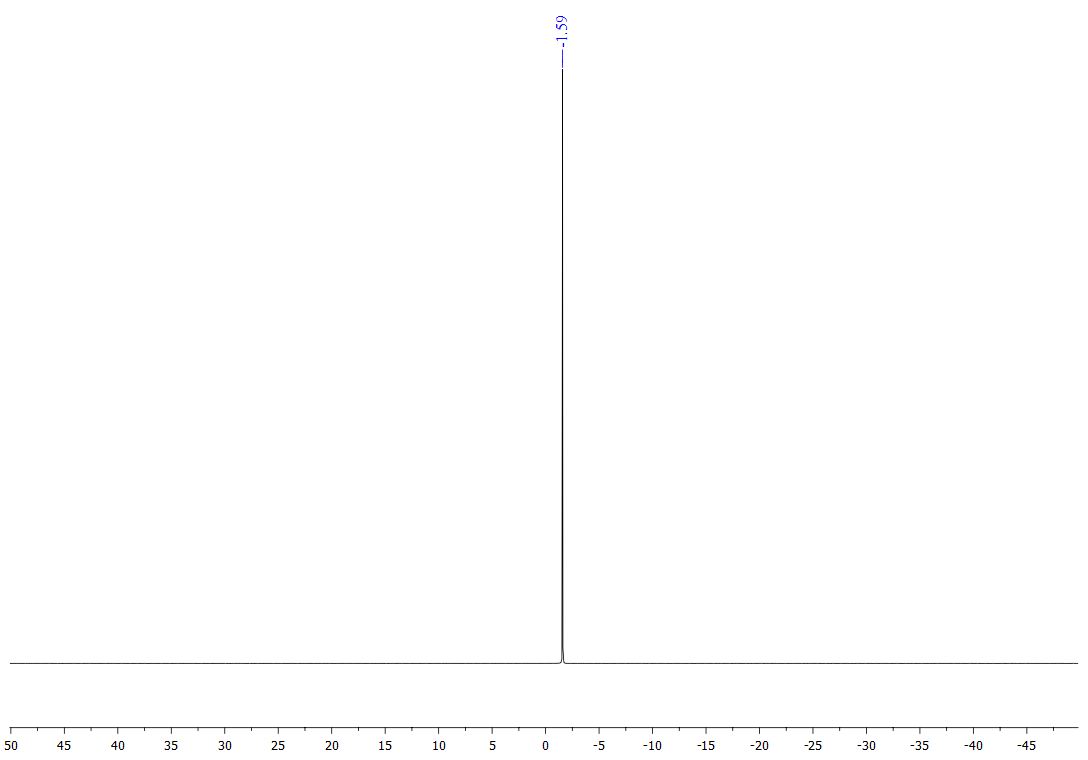
Figure S5.4.4** ^7^Li NMR (155 MHz, CD_3_CN, 295 K) spectrum of lithium bis(perfluorinated pinacolato)borate, Li[B(pp)_2_]·DME after 5 weeks air exposure.

**
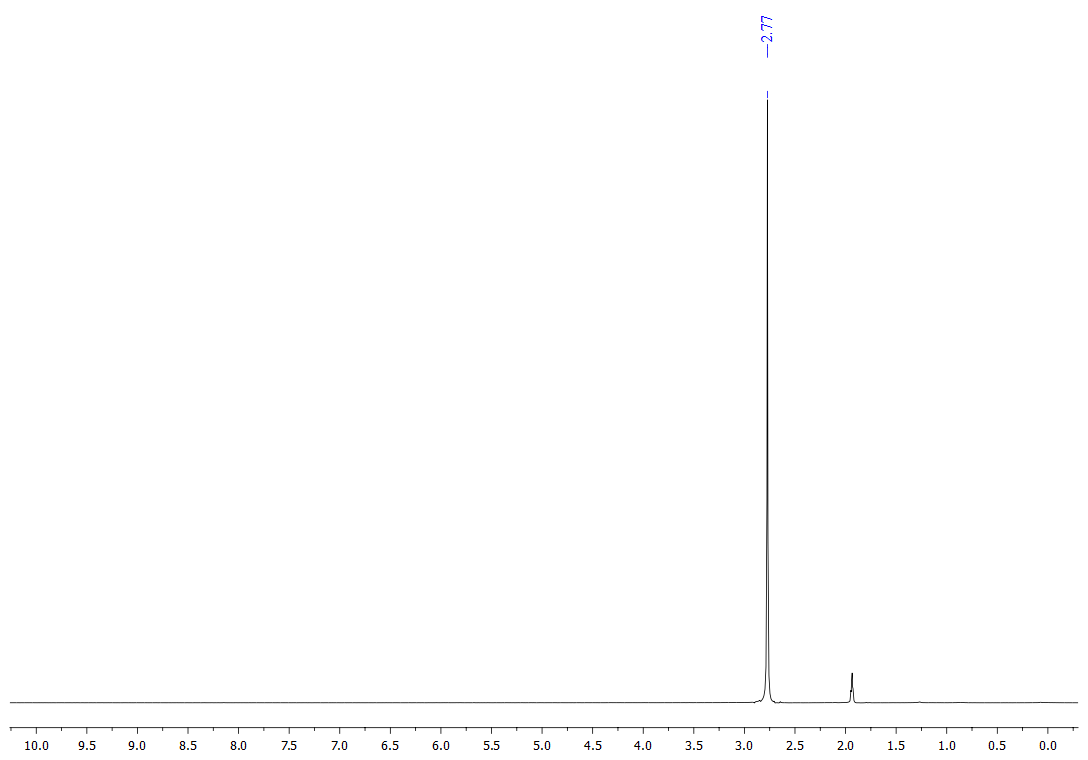
Figure S5.4.5** ^1^H NMR (400 MHz, CD_3_CN, 295 K) spectrum of unsolvated lithium bis(perfluorinated pinacolato)borate, Li[B(pp)_2_] after 5 weeks air exposure.

**
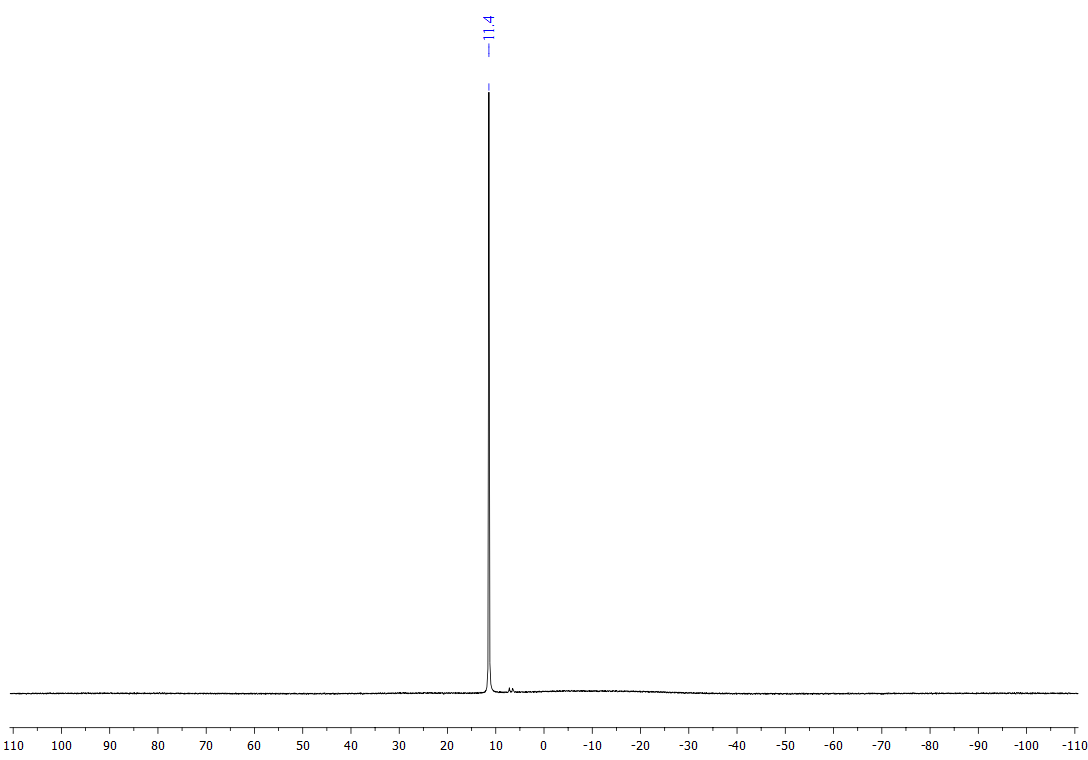
Figure S5.4.6** ^11^B NMR (128 MHz, CD_3_CN, 295 K) spectrum of unsolvated lithium bis(perfluorinated pinacolato)borate, Li[B(pp)_2_] after 5 weeks air exposure.


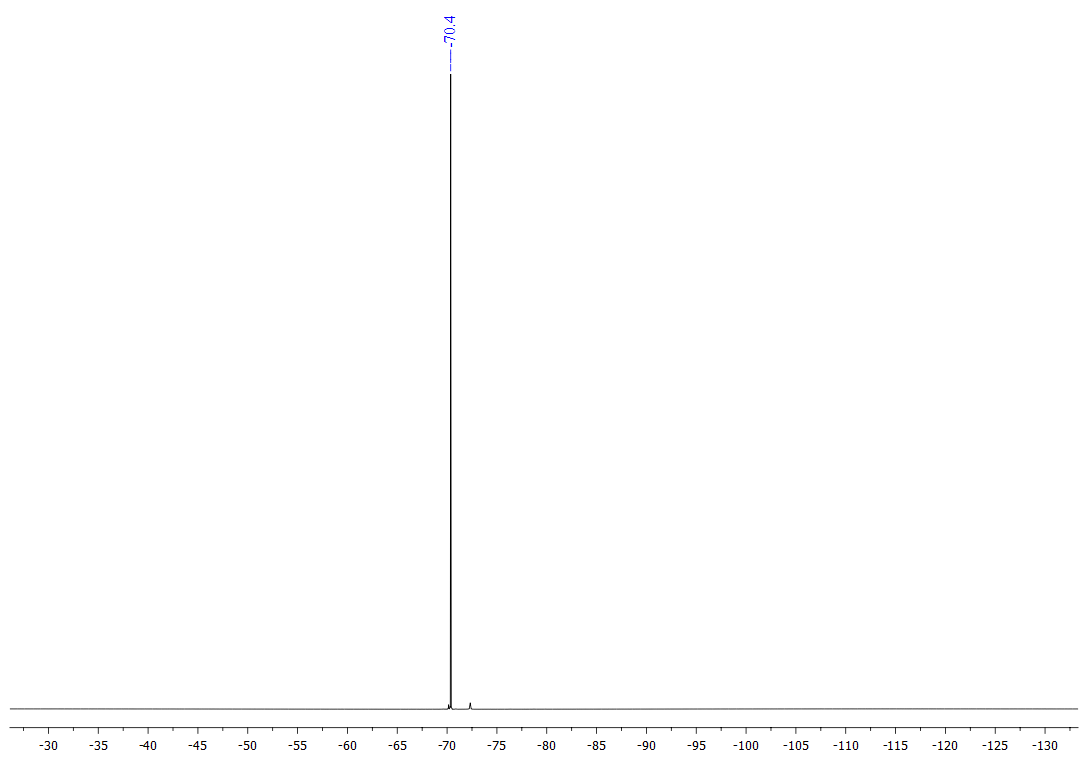
**Figure S5.4.7** ^19^F NMR (376 MHz, CD_3_CN, 295 K) spectrum of unsolvated lithium bis(perfluorinated pinacolato)borate, Li[B(pp)_2_] after 5 weeks air exposure.


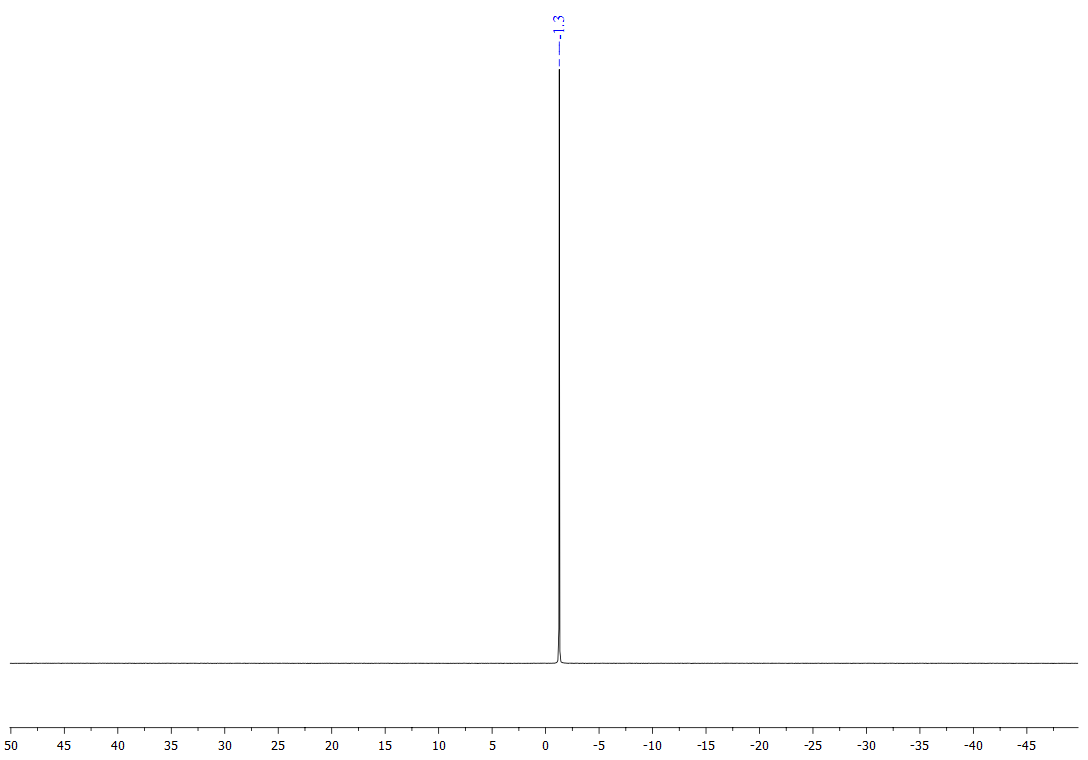
**Figure S5.4.8** ^7^Li NMR (155 MHz, CD_3_CN, 295 K) spectrum of unsolvated lithium bis(perfluorinated pinacolato)borate, Li[B(pp)_2_] after 5 weeks air exposure.

**S5.5 NMR spectra of air exposed lithium hexafluorophosphate**

**Figure S5.5.1** ^1^H NMR (400 MHz, (CD_3_)_2_SO, 295 K) spectrum of lithium hexafluorophosphate, LiPF_6_ after 1 day air exposure.


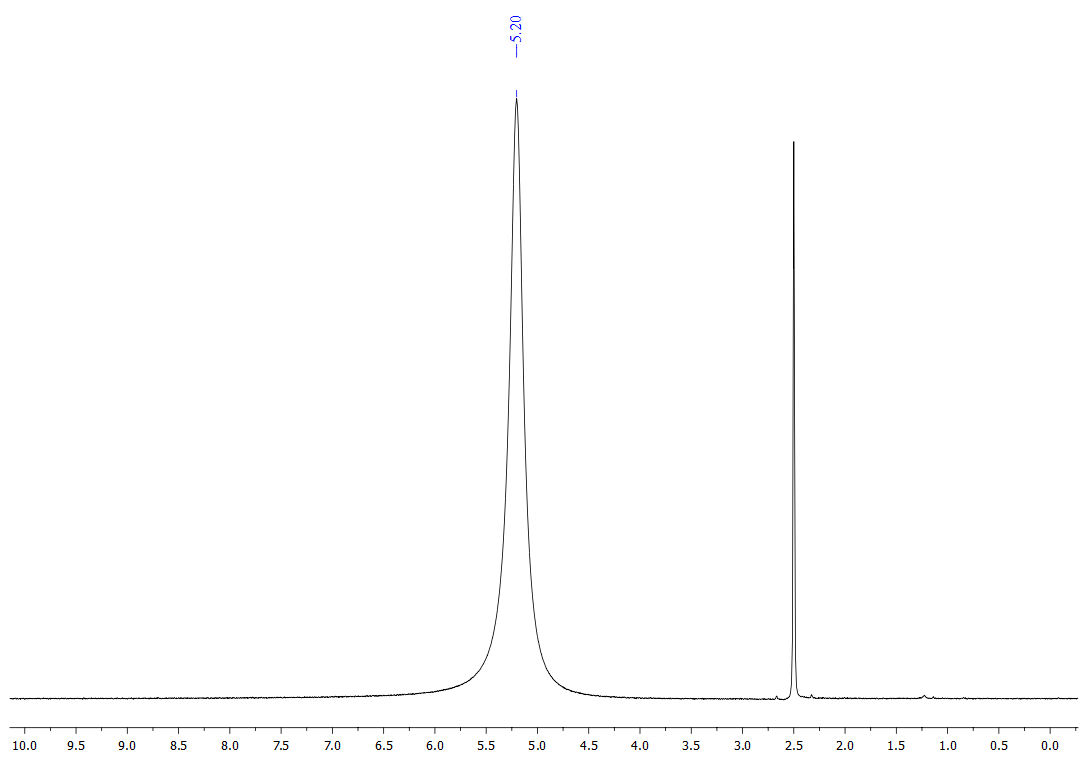


**Figure S5.5.2** ^31^P NMR (162 MHz, (CD_3_)_2_SO, 295 K) spectrum of lithium hexafluorophosphate, LiPF_6_ after 1 day air exposure.


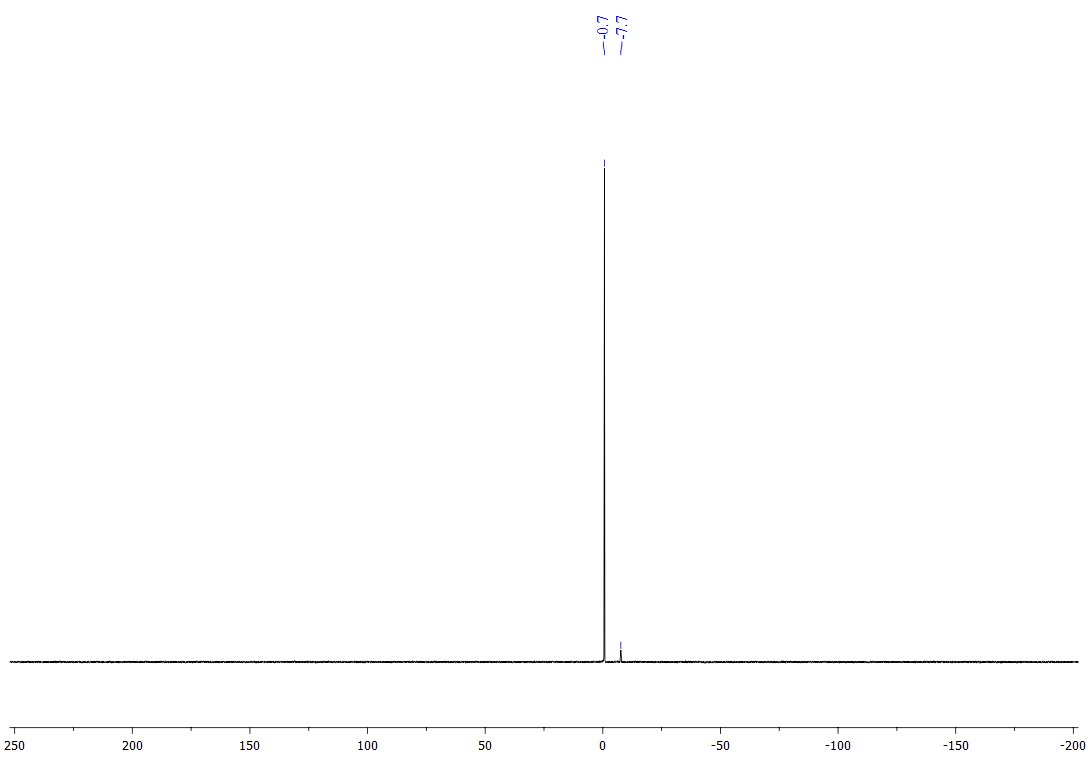

**Figure S5.5.3** ^7^Li NMR (155 MHz, (CD_3_)_2_SO, 295 K) spectrum of lithium hexafluorophosphate, LiPF_6_ after 1 day air exposure.


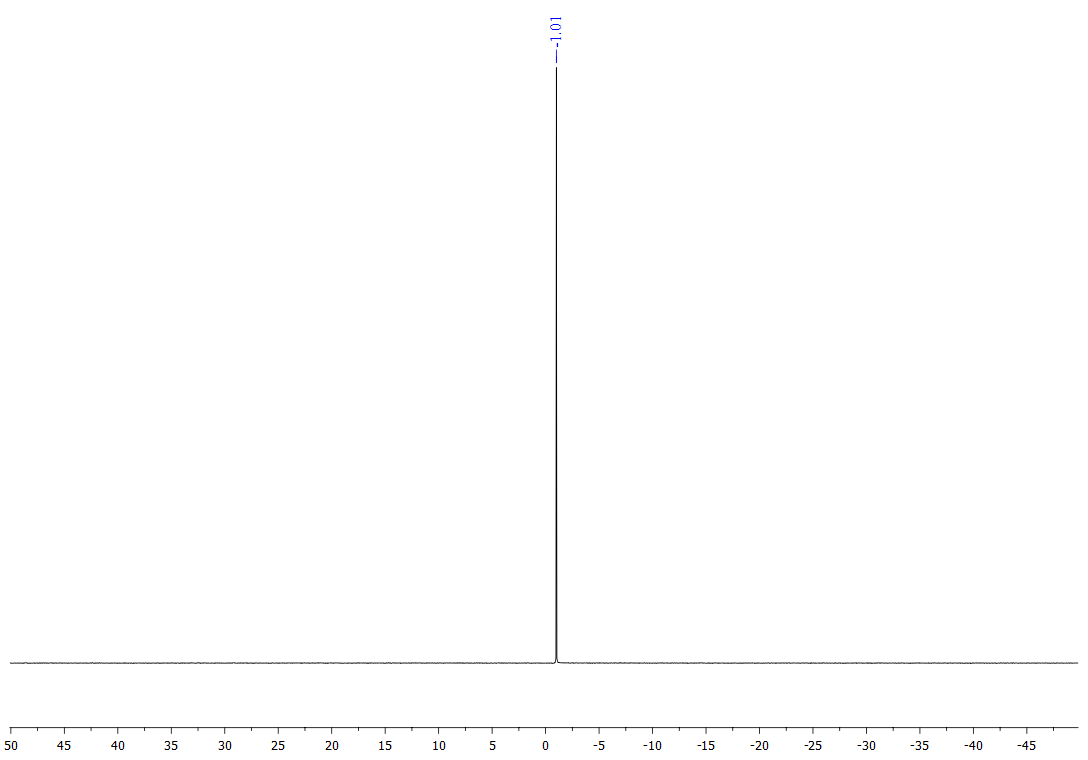


# **S6 Photo of NMR solutions of Li-ion cycled electrolyte.**


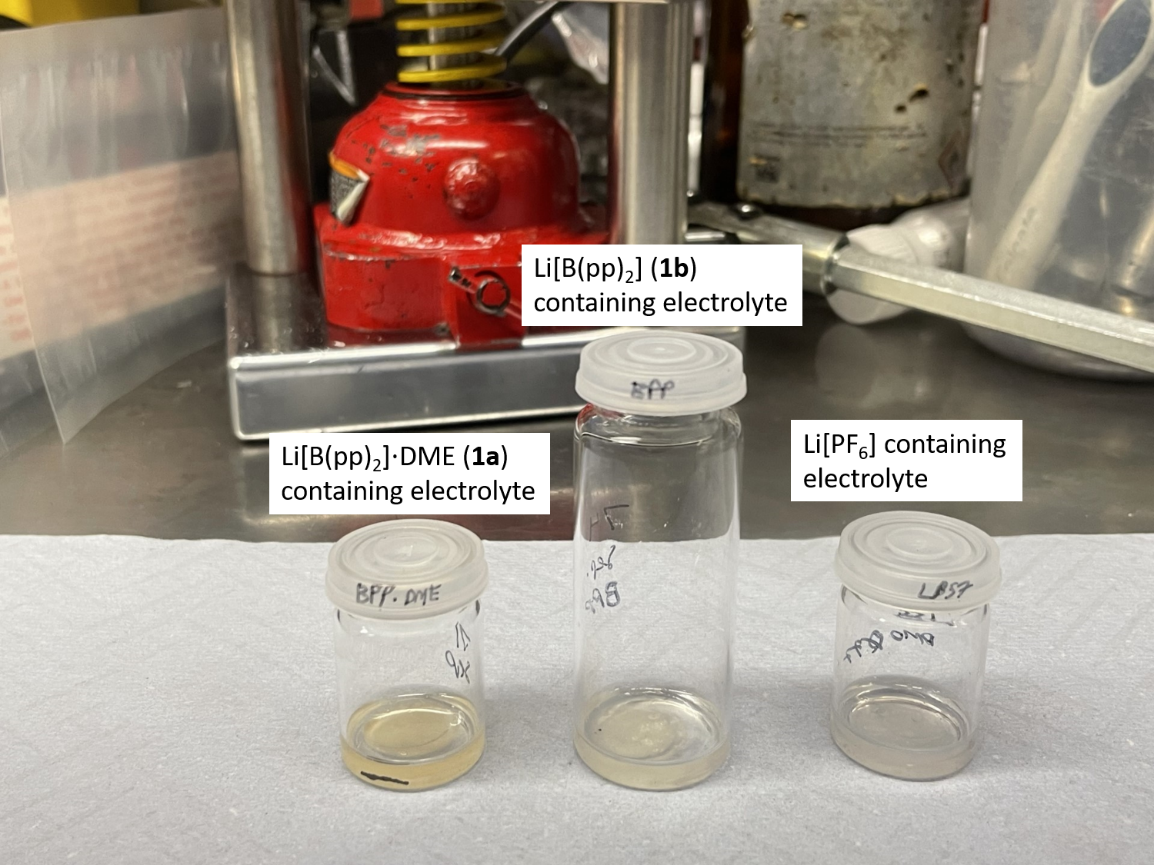


**Figure S6.1** Photo of electrolytes from separator soaked in DMSO-*d*_6_.
